# Is ChatGPT accurate and reliable in answering questions regarding head and neck cancer?

**DOI:** 10.3389/fonc.2023.1256459

**Published:** 2023-12-01

**Authors:** Oğuz Kuşcu, A. Erim Pamuk, Nilda Sütay Süslü, Sefik Hosal

**Affiliations:** ^1^Department of Otorhinolaryngology, School of Medicine, Hacettepe University, Ankara, Türkiye; ^2^Private Practitioner, Ankara, Türkiye; ^3^Department of Otorhinolaryngology, School of Medicine, Atılım University, Ankara, Türkiye

**Keywords:** ChatGPT 4, head and neck (H&N) cancer, head and neck, artificial intelligence, chatbot, information literacy, natural language processing, machine learning

## Abstract

**Background and objective:**

Chat Generative Pre-trained Transformer (ChatGPT) is an artificial intelligence (AI)-based language processing model using deep learning to create human-like text dialogue. It has been a popular source of information covering vast number of topics including medicine. Patient education in head and neck cancer (HNC) is crucial to enhance the understanding of patients about their medical condition, diagnosis, and treatment options. Therefore, this study aims to examine the accuracy and reliability of ChatGPT in answering questions regarding HNC.

**Methods:**

154 head and neck cancer-related questions were compiled from sources including professional societies, institutions, patient support groups, and social media. These questions were categorized into topics like basic knowledge, diagnosis, treatment, recovery, operative risks, complications, follow-up, and cancer prevention. ChatGPT was queried with each question, and two experienced head and neck surgeons assessed each response independently for accuracy and reproducibility. Responses were rated on a scale: (1) comprehensive/correct, (2) incomplete/partially correct, (3) a mix of accurate and inaccurate/misleading, and (4) completely inaccurate/irrelevant. Discrepancies in grading were resolved by a third reviewer. Reproducibility was evaluated by repeating questions and analyzing grading consistency.

**Results:**

ChatGPT yielded “comprehensive/correct” responses to 133/154 (86.4%) of the questions whereas, rates of “incomplete/partially correct” and “mixed with accurate and inaccurate data/misleading” responses were 11% and 2.6%, respectively. There were no “completely inaccurate/irrelevant” responses. According to category, the model provided “comprehensive/correct” answers to 80.6% of questions regarding “basic knowledge”, 92.6% related to “diagnosis”, 88.9% related to “treatment”, 80% related to “recovery – operative risks – complications – follow-up”, 100% related to “cancer prevention” and 92.9% related to “other”. There was not any significant difference between the categories regarding the grades of ChatGPT responses (p=0.88). The rate of reproducibility was 94.1% (145 of 154 questions).

**Conclusion:**

ChatGPT generated substantially accurate and reproducible information to diverse medical queries related to HNC. Despite its limitations, it can be a useful source of information for both patients and medical professionals. With further developments in the model, ChatGPT can also play a crucial role in clinical decision support to provide the clinicians with up-to-date information.

## 1 Introduction

As humanity embarks on a new epoch marked by significant advancements in artificial intelligence (AI), the integration of AI into the realm of bioinformatics offers vast potential for healthcare improvement. The Chat Generative Pre-trained Transformer (ChatGPT) is a recent AI model, designed to generate human-like conversational dialogue in response to textual input by predicting answers from a vast database of publicly undisclosed resources including websites, books, and articles up to 2021 (1–3). It is fine-tuned for conversational tasks through reinforcement learning from human feedback, enhancing accuracy and coherence (4). Since its initial public introduction, ChatGPT has rapidly gained popularity, largely attributed to its proficiency in handling a broad array of tasks via an intuitive user interface. While the amalgamation of ChatGPT into medicine has been met with mixed reactions, with some researchers lauding its potential to bolster clinical tasks and others raising concerns over its medical writing, safety, and legal issues (5–7). Recent literature has documented studies on its application across various medical fields (8–10).

Head and neck cancer (HNC) ranks as the seventh most common form of malignancy globally, with an annual incidence of approximately 660,000 new cases (11, 12). Alarmingly, the overall incidence of HNC continues to rise, projected to increase annually by 30% by the year 2030 (11, 12). This uptick is seen across both developed and developing nations (13). In the context of an increasingly digital communication landscape, patients, inclusive of those diagnosed with HNC, have begun to supplement their reliance on medical professionals with medical information gleaned from search engines and AI chatbots like ChatGPT. However, the reliability and accuracy of the information provided by ChatGPT, particularly in answering patient inquiries related to specific medical conditions like HNC, remains to be fully validated. The performance of ChatGPT is also of significance when considering its potential utility to medical professionals. Consequently, this study aims to investigate the accuracy and reliability of ChatGPT’s responses to questions pertaining to HNC.

## 2 Methods

Institutional review board approval was not sought for this prospective study due to the absence of patient-level data. Authors have no affiliation or involvement with OpenAI Inc., the developer of ChatGPT.

### 2.1 Data acquisition

**Figure 1:**
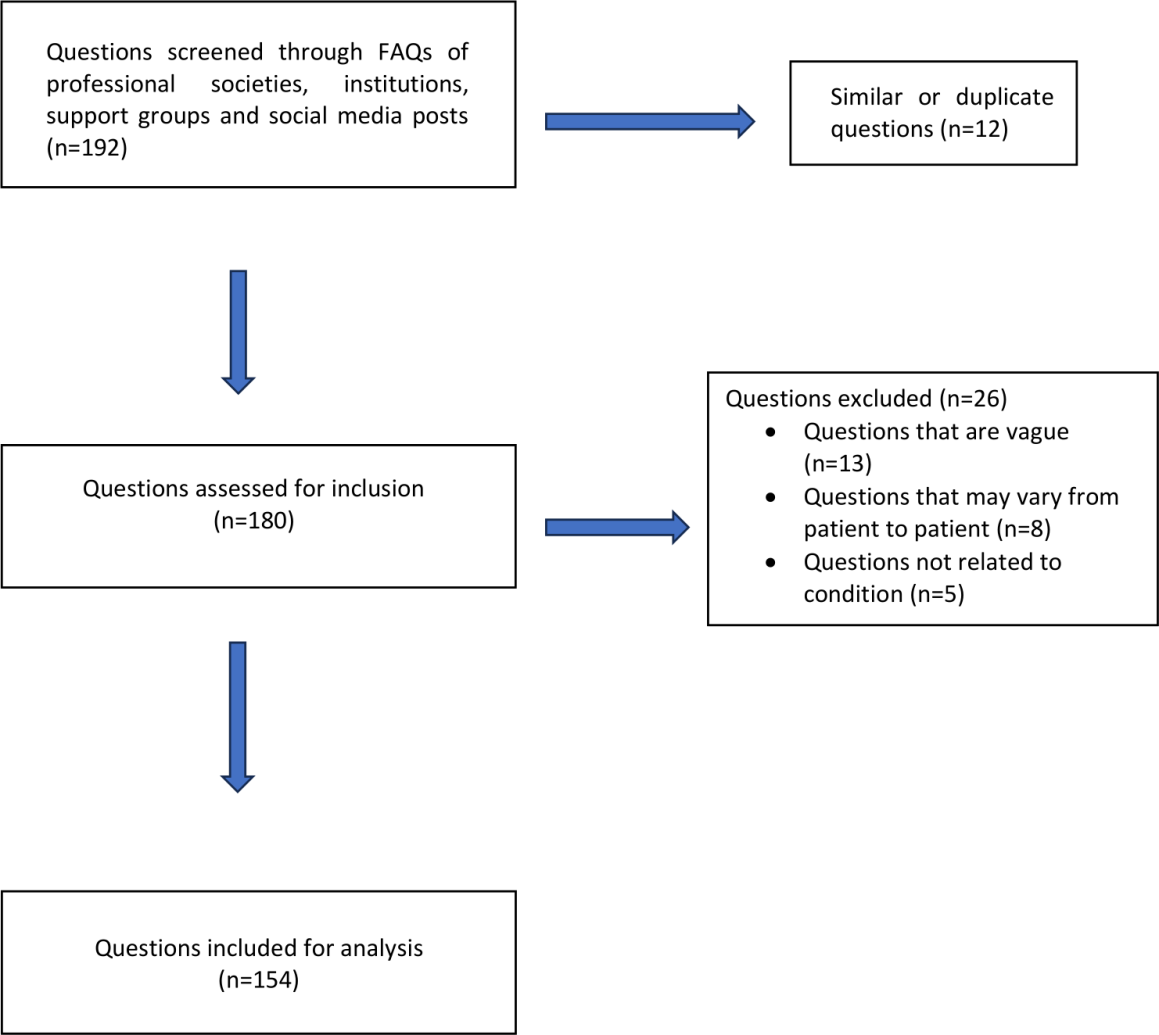
Flow chart of head and neck cancer-related question selection.

The data set of questions was compiled from queries frequently posted by professional institutions and societies such as the American Head & Neck Society (AHNS), National Cancer Institute, and the Medline Plus Medical Encyclopedia. In the interest of inclusivity and patient representation, questions sourced from patient support groups and social media posts were incorporated. These questions underwent a rigorous screening process by three authors to evaluate their eligibility for inclusion in the study. The selected questions pertained specifically to HNC. Exclusion criteria were implemented to disqualify questions with similar meanings, questions that were subjective or could vary between patients (e.g., “What is the likelihood of my larynx cancer recurring?”), vague inquiries (e.g., “How will tongue cancer affect my body?”), and non-medical questions related to the condition (e.g., “What are the head and neck cancer online support groups?”) (**Figure 1**).

In total, 154 questions were enlisted to solicit responses from ChatGPT. The questions were systematically categorized into distinct groups based on their corresponding subjects: (1) basic knowledge, (2) diagnosis, (3) treatment, (4) recovery, operative risks, complications and follow-up (5) cancer prevention, and (6) other. Grammatical adjustments were made to certain questions to ensure clarity and precision.

The selection was predominantly patient-oriented, seeking to evaluate the AI model’s ability to provide information to potential patient inquiries (e.g., “Is head and neck cancer contagious?” or “Can I swim after undergoing a total laryngectomy?”). Additionally, we included questions featuring technical medical terminology to simulate scenarios that patients might encounter during their medical journey (e.g., “What does lymphovascular invasion in my throat cancer surgical pathology report mean?” or “My lip resection specimen for lip cancer report indicates 0.5 cm surgical margins. Is this adequate for tumor control?”). Complex questions were also included to assess the model’s capacity to assist medical professionals (e.g., “Is a tracheotomy always mandatory before a cordotomy for bilateral vocal fold paralysis due to radiotherapy?” Or “Is a sentinel node biopsy applicable in a T3N1M0 tongue carcinoma case?”).

### 2.2 Inquiries and response generation

In this study, we used ChatGPT Plus (based on GPT-4, March 13, 2023 version), an advanced subscription-based version of ChatGPT that was released in November 2022. The questions were submitted on June 13, 2023. Each question was entered independently using the “New Chat” function. In alignment with previous studies, each question was entered twice to assess the reproducibility of ChatGPT’s responses (9, 10). ChatGPT was not provided with any feedback. The current version of ChatGPT Plus has a limit of 50 messages every three hours. All the questions asked of ChatGPT were in English. (**Supplementary File 1**).

### 2.3 Grading system

Two experienced head and neck surgeons, currently active in academic practice, independently reviewed and graded the responses to questions for accuracy and reproducibility. The reviewers evaluated the accuracy of responses according to the following scale

#### 2.3.1 Comprehensive/Correct

Data is accurate and comprehensive; a head and neck surgeon would add no further information if asked by a patient.

#### 2.3.2 Incomplete/Partially Correct

The provided data is correct; however, additional pertinent information could be provided if asked by a patient.

#### 2.3.3 Mixed

Contains accurate and inaccurate data, potentially misleading.

#### 2.3.4 Completely Inaccurate/Irrelevant

The data provided is completely inaccurate or irrelevant.

Reproducibility was assessed based on the consistency of the two responses to each individual question. If the responses were similar, only the first response from ChatGPT was graded. In cases where the responses differed, both were independently graded by the reviewers. If the grades differed, the responses were deemed non-reproducible. Any discrepancies in the accuracy and reproducibility of responses between the two reviewers were independently reviewed and resolved by a third reviewer, a highly experienced academic head and neck surgeon, who was blinded to the initial reviews. Reproducibility was evaluated for each category of questions and compared between categories.

### 2.4 Statistical analysis

The proportions of each grade among the responses were calculated and reported as percentages. Categorical variables were examined using the χ2 test and Fisher’s exact test. The kappa statistic was used to measure inter-rater agreement, revealing a substantial agreement between Reviewers 1 and 2 (Kappa value 0.657, p<0.001). All analyses were performed using IBM SPSS v.25.0 (IBM Corp.).

## 3 Results

A total of 154 inquiries pertaining to HNC were directed to ChatGPT. The model provided “comprehensive/correct” responses to 133 out of 154 (86.4%) questions. Meanwhile, the rates of “incomplete/partially correct” and “mixed with accurate and inaccurate data/misleading” responses were noted to be 11% and 2.6%, respectively. It is significant to note that there were no instances of “completely inaccurate/irrelevant” responses.

Questions were categorized as follows: basic knowledge (36 questions, 23.4%), diagnosis (27 questions, 17.5%), treatment (27 questions, 17.5%), recovery - operative risks – complications – follow-up (40 questions, 26%), cancer prevention (10 questions, 6.5%) and other (14 questions, 9.1%).

**Figure 2:**
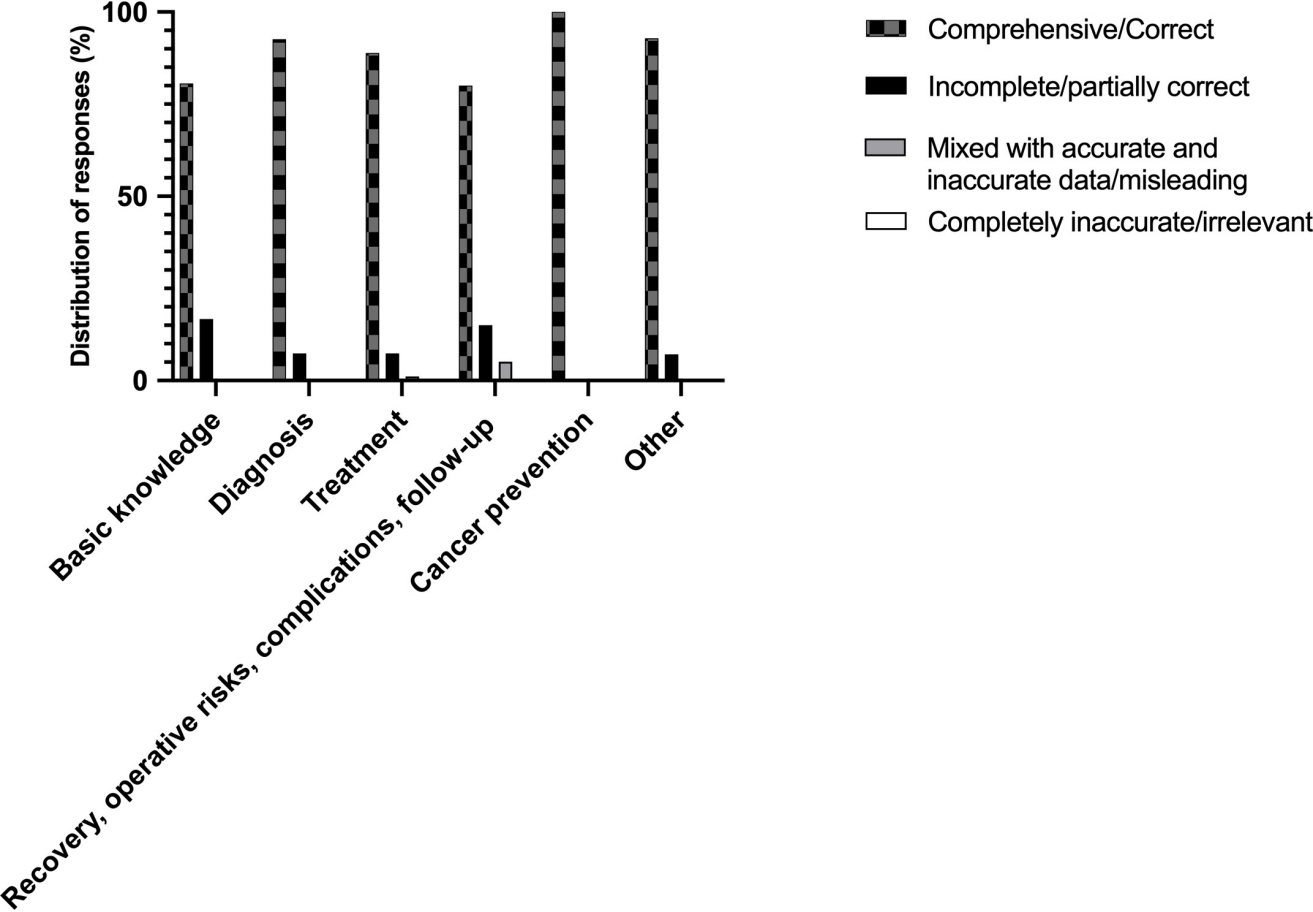
Graphical representation of grades by ChatGPT according to category of the questions.

**Table 1** shows the distribution of grades generated by ChatGPT with regard to category of inquiry. There was no response detected in under “mixed with accurate and inaccurate data/misleading” description in the diagnosis, cancer prevention, and other categories. The highest rate of “comprehensive/correct” responses (100%) was recorded in the inquiries regarding cancer prevention. A graphical representation of these findings is depicted in **Figure 2**. Notably, there were no significant differences between the categories regarding the grades of ChatGPT responses (p=0.88).

**Table 1:** Distribution of the responses from ChatGPT according to inquiry category.

	No. of questions (%)
Basic knowledge (n=36)	
Comprehensive/correct	29 (80.6)
Incomplete/partially correct	6 (16.7)
Mixed with accurate and inaccurate data/misleading	1 (2.8)
Completely inaccurate/irrelevant	–
Diagnosis (n=27)	
Comprehensive/correct	25 (92.6)
Incomplete/partially correct	2 (7.4)
Mixed with accurate and inaccurate data/misleading	–
Completely inaccurate/irrelevant	–
Treatment (n=27)	
Comprehensive/correct	24 (88.9)
Incomplete/partially correct	2 (7.4)
Mixed with accurate and inaccurate data/misleading	1 (3.7)
Completely inaccurate/irrelevant	–
Recovery - operative risks – complications – follow-up (n=40)	
Comprehensive/correct	32 (80)
Incomplete/partially correct	6 (15)
Mixed with accurate and inaccurate data/misleading	2 (5)
Completely inaccurate/irrelevant	–
Cancer prevention (n=10)	
Comprehensive/correct	10 (100)
Incomplete/partially correct	–
Mixed with accurate and inaccurate data/misleading	–
Completely inaccurate/irrelevant	–
Other (n=14)	
Comprehensive/correct	13 (92.9)
Incomplete/partially correct	1 (7.1)
Mixed with accurate and inaccurate data/misleading	–
Completely inaccurate/irrelevant	–

**Figure 3:**
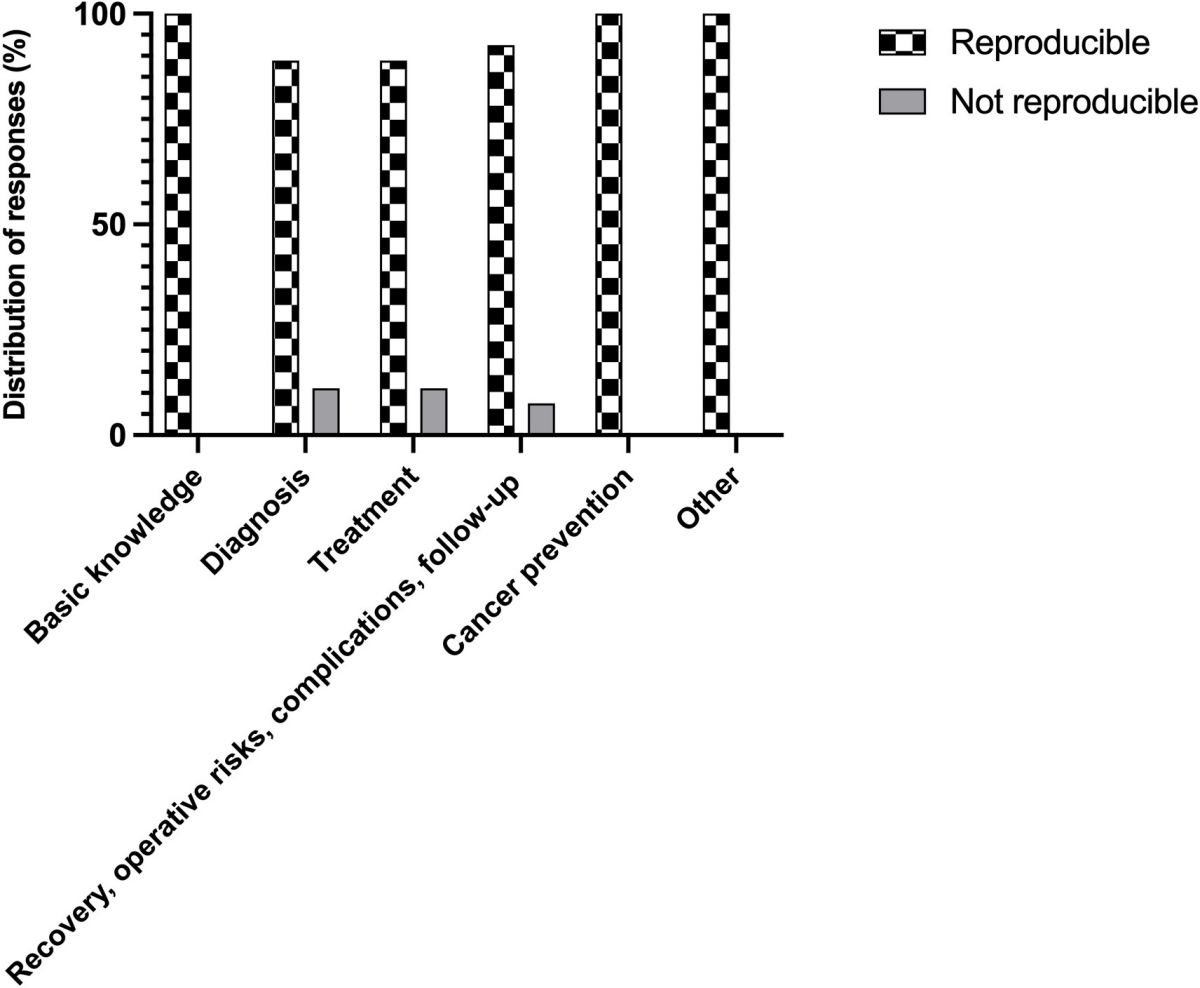
Reproducibility of the responses with regard to category of the questions.

Overall, the reproducibility rate of the model was 94.1% (145 questions). Reproducibility was 100% for the categories of basic knowledge, cancer prevention, and other. However, this rate decreased to 88.9% for diagnosis, 88.9% for treatment, and 92.5% for recovery – operative risks – complications – follow-up categories (as illustrated in **Figure 3**). No significant differences were observed between the categories concerning reproducibility (p= 0.309).

## 4 Discussion

AI is increasingly being utilized in various fields, including healthcare, where it has a promising role in improving patient education or providing medical assistance to healthcare professionals. Various AI models and machine learning algorithms have been developed recently which outperformed the existing conventional methods regarding diagnosis and survival prediction in HNC. Constantino et al. reported a machine learning model which has a high specifity and negative predictive value for predicting lymph node metastasis in salivary gland cancer (14). Fatapour et al. developed an effective machine learning model to successfully predict oral cancer recurrence whereas, Choi et al. reported a successful AI model to predict the survival in patients with laryngeal cancer (15, 16). Introduction of ChatGPT in November 2022 provided public access to vast amounts of information on numerous topics, including complex medical conditions such as HNC. In this study, we investigated the accuracy and reliability of ChatGPT regarding commonly asked patient questions related to HNC. Our questions also included some detailed technical inquiries to simulate the conditions under which a medical professional might seek assistance in the clinical decision-making process.

Due to its recent emergence, there are limited studies regarding the role of ChatGPT in the field of otolaryngology. Park et al. tested the ChatGPT’s ability to discuss its own potential role, ethical considerations and limitations in clinical otolaryngology (17). They concluded that the model has great potential to assist the clinicians in their decision making process and provide tailored care to each patient (17). However, due to the model’s potential for error and highly dependence on pretrained available data, they also emphasized the importance of understanding the limitations and using it cautiously with a priority on patient safety. In their comparative research letter, Ayoub et al. evaluated and contrasted the efficacy of ChatGPT and Google Search as resources for postoperative patient instructions following pediatric otolaryngology procedures (18). Their results showed that ChatGPT had lower scores than Google Search and institution-specific instructions in terms of understandability, actionability and procedure-specific content. Nevertheless, given its adaptability to various literacy levels and its capacity to provide direct, comprehensively articulated, and detailed responses, ChatGPT could prove advantageous for both patients and clinicians, especially in situations where alternate sources of information are constrained (18). There are also empirical studies suggesting that ChatGPT shows promise as a tool in the clinical decision-making process, particularly for patients who are being considered for sialendoscopy (19). Moreover, research conducted by Hoch et al. demonstrated that ChatGPT may potentially serve as a supplementary instrument for the preparation of otolaryngology board certification examinations (20).

Patients who receive a cancer diagnosis often present with heightened emotional sensitivity and anxiety, particularly concerning their disease prognosis and survival rates. Furthermore, the intricacies of surgical procedures and/or chemoradiotherapy, including their associated risks and benefits, are frequently not well-understood by a significant proportion of these patients. Consequently, the delivery of comprehensive and digestible information becomes crucial in mitigating any supplementary stress they might experience. Traditional clinic consultations may not provide sufficient time to extensively educate patients about their condition and treatment options. Additionally, the occasional inaccessibility of healthcare professionals can further complicate patient education. ChatGPT has substantially elevated this online educational process, synergizing artificial intelligence capabilities with an accessible, publicly available, and free platform. However, the performance of this tool necessitates rigorous evaluation. In light of this, Johnson et al. conducted a comprehensive analysis of ChatGPT’s proficiency in responding to queries posted on the “Common Cancer Myths and Misconceptions” webpage, juxtaposing its responses with those provided by the National Cancer Institute (NCI) to the same queries (21). The findings indicated that the answers provided by the NCI had an overall accuracy rate of 100%, while the responses from ChatGPT to questions 1 through 13 exhibited an accuracy rate of 96.9% (k=–0.03, SE 0.08) (21). There was no statistically significant discrepancy in terms of word count or readability between the NCI and ChatGPT responses. Therefore, it can be asserted that ChatGPT furnished accurate information regarding prevalent cancer myths and misconceptions.

Our findings indicate that the majority of responses from ChatGPT were accurate, with 86.4% of receiving a “comprehensive/correct” rating on our grading scale. Importantly, none of the responses were classified as “completely inaccurate/irrelevant”. Furthermore, the model demonstrated high reproducibility across all topics, and performed commendably without any significant differences between them. Our study may provide an early evidence base demonstrating the immense potential of this innovative platform in the field of AI-driven medical information, specifically concerning HNC. With additional validation, ChatGPT or similar tools could serve as invaluable resources for rapid medical information retrieval in high-speed clinical settings, thereby enhancing efficiency and aiding clinicians in their complex decision-making processes. However, we are only in the early stages of the era of AI-provided medical services, and it is currently not advisable to rely solely on the existing version of ChatGPT as the only source of medical information. With the advent of newer versions that are appropriately trained by medical experts using the most current medical literature, accurate medical information could be rapidly disseminated to both patients and medical professionals.

To the best of our knowledge, this is the first study investigating the application of ChatGPT in the field of HNC as of July 2023. The questions incorporated in our study were sourced from reputable institutions and societies, as well as patient support groups. The assessment of accuracy and reproducibility was conducted by independent evaluators. However, it is necessary to acknowledge the limitations inherent in both the ChatGPT model and our study. Pertaining to ChatGPT, the current version’s knowledge cutoff is up to September 2021. Omission of data from the past two years could potentially impact the precision of the responses. Furthermore, the reliability of ChatGPT is fundamentally contingent upon the quality of the training data. Hence, given the undisclosed sources of the model, it remains questionable whether the training was based on the most reliable and accurate medical literature. Thirdly, the latest version of ChatGPT, which exhibits superior performance compared to the publicly available version, is accessible only via paid subscription, potentially constraining the public’s access to more accurate knowledge.

In relation to our study, we utilized a grading method similar to those employed in previous studies (9, 10). However, alternate methodologies exist for evaluating the performance of the model (22, 23), and it remains unclear which approach is the most effective in assessing the accuracy of ChatGPT responses. Lastly, the number of questions presented to the model was restricted to those found within the investigated institutions, societies, and patient support groups. Although the total quantity of questions was comparable with other studies, it remains uncertain as to what the optimal number of queries should be in order to effectively evaluate the model, leading to potential arbitrariness (9, 10, 23).

## 5 Conclusion

ChatGPT has proven to generate markedly accurate and reproducible responses to a wide range of medical inquiries pertaining to HNC. Despite its inherent limitations, it may serve as a beneficial source of information for both patients and healthcare professionals. As the model undergoes further refinement, ChatGPT could potentially assume a vital role in clinical decision support, equipping clinicians with current information. It is imperative that future research efforts strive to delineate the risks and benefits of employing this AI model in the context of HNC, as well as in diverse medical domains.

## Data availability statement

The raw data supporting the conclusions of this article will be made available by the authors, without undue reservation.

## Author contributions

OK: Conceptualization, Project administration, Resources, Supervision, Validation, Visualization, Writing – review & editing. AP: Conceptualization, Data curation, Formal Analysis, Investigation, Methodology, Writing – original draft, Writing – review & editing. NS: Project administration, Supervision, Validation, Visualization, Writing – review & editing. SH: Investigation, Project administration, Resources, Supervision, Validation, Visualization, Writing – review & editing.
