# Supplementary material for: Is ChatGPT accurate and reliable in answering questions regarding head and neck cancer?
Source: Front Oncol. 2023 Dec 1;13:1256459. doi: 10.3389/fonc.2023.1256459 (PMC10722294; doi:10.3389/fonc.2023.1256459)
Supplement: Supplementary file 1 [file Table_1.docx]

Supplementary Table 1- Grading of the responses generated by ChatGPT for questions regarding head and neck cancer

| Questions | Response 1 | Response 2 | Reviewer 1 grades  1-Comprehensive/Correct  2-Incomplete/partially correct  3- Mixed with accurate and inaccurate data/ misleading  4-Completely inaccurate/ irrelevant | Reviewer 2 grades  1- Comprehensive/Correct  2-Incomplete/partially correct  3- Mixed with accurate and inaccurate data/misleading  4-Completely inaccurate/ irrelevant | Reviewer 3 grades for the responses with discrepancies |
| --- | --- | --- | --- | --- | --- |
| ***BASIC KNOWLEDGE*** |  |  |  |  |  |
| 1. What is HPV-related throat cancer? | HPV-related throat cancer, also known as oropharyngeal cancer, refers to a type of cancer that develops in the back of the throat, including the base of the tongue and the tonsils, as a result of infection with certain strains of the human papillomavirus (HPV). HPV is a common sexually transmitted infection, and while most HPV infections clear up on their own without causing any symptoms or health problems, persistent infection with certain high-risk HPV types can lead to the development of various cancers, including throat cancer.  The predominant HPV type associated with throat cancer is HPV-16, although other high-risk types like HPV-18, 31, 33, and 35 can also be implicated. It's important to note that not everyone who contracts HPV will develop throat cancer, as most infections do not progress to cancer. However, long-lasting HPV infections can increase the risk of developing certain cancers over time.  HPV-related throat cancer typically affects both men and women in their middle age. The symptoms of throat cancer can include a persistent sore throat, difficulty swallowing, ear pain, hoarseness, a lump in the neck, and unexplained weight loss. However, these symptoms can also be caused by other conditions, so it's important to consult a healthcare professional for an accurate diagnosis.  Treatment for HPV-related throat cancer may involve a combination of therapies such as surgery, radiation therapy, and chemotherapy. The prognosis for HPV-related throat cancer is generally better than that of throat cancer caused by other factors such as smoking and alcohol consumption. This is because HPV-related throat cancer tends to respond well to treatment and has a higher survival rate.  Prevention is crucial in reducing the risk of HPV-related throat cancer. Vaccination against HPV is recommended for both males and females, typically before they become sexually active. The HPV vaccine provides protection against the high-risk HPV types most commonly associated with cervical, anal, and throat cancers.  If you suspect you may have HPV-related throat cancer or have concerns about HPV infection, it is important to consult with a healthcare professional who can provide a proper evaluation, diagnosis, and appropriate treatment options. | Comparable | 1 | 1 | Not applicable |
| 2. Can i get nasopharynx cancer from a virus? | Yes, certain types of viruses can increase the risk of developing nasopharyngeal cancer.  The most significant one is the Epstein-Barr virus (EBV), which is a type of herpes virus. It's very common and most people get infected with this virus at some point in their lives, usually in childhood. In most cases, it does not cause any symptoms or just causes a mild illness. However, in certain circumstances, it can lead to more serious conditions, including certain types of cancer, such as nasopharyngeal cancer.  It's important to note that while EBV infection can increase the risk, not everyone who has the virus will develop nasopharyngeal cancer. The development of cancer is usually the result of a combination of factors, including genetics, environmental factors, and lifestyle choices, such as smoking and alcohol use.  Having EBV infection alone is not enough to cause nasopharyngeal cancer. It's not completely understood why some people with EBV go on to develop cancer while others do not, but it's likely related to their genetic makeup and other environmental factors.  If you're concerned about your risk, it's important to talk to your doctor. They can provide you with more information and suggest ways to reduce your risk. | Comparable | 1 | 1 | Not applicable |
| 3. What is oropharyngeal cancer? | Oropharyngeal cancer is a type of cancer that originates in the oropharynx, the middle part of the throat which includes the base of the tongue, the tonsils, the soft palate, and the walls of the pharynx.  The primary types of cells where this cancer can originate include squamous cells that line the oropharynx, and minor salivary gland cells. However, squamous cell carcinomas are more common.  Risk factors for oropharyngeal cancer include tobacco use (including smokeless tobacco), heavy alcohol consumption, poor oral hygiene, a diet lacking fruits and vegetables, and infection with the human papillomavirus (HPV), particularly HPV type 16.  Symptoms of oropharyngeal cancer may include persistent sore throat, earaches, hoarseness, unexplained weight loss, difficulty swallowing or breathing, and a lump or sore in the throat or mouth that doesn't heal.  Diagnosis is often made through a combination of physical examination, imaging studies, and biopsy of suspicious tissues. Treatment depends on the stage of the cancer and typically involves a combination of surgery, radiation therapy, and chemotherapy.  Like all cancers, early detection of oropharyngeal cancer can significantly improve the prognosis. Therefore, it's important for people with persistent symptoms, especially those who have risk factors, to seek medical attention. | Comparable | 1 | 1 | Not applicable |
| 4. What is metastatic squamous cell cancer of the neck? | Metastatic squamous cell neck cancer is a form of cancer that originates in squamous cells, which are flat, scale-like cells that compose the outermost layer of skin, the epidermis, and some internal tissues. However, the term "metastatic squamous cell neck cancer" usually refers to squamous cell carcinomas that have originated from other sites such as the head and neck regions and spread (or metastasized) to lymph nodes in the neck.  Typically, when someone is diagnosed with this type of cancer, the primary site, such as the oral cavity, pharynx (throat), or larynx (voice box), is identified and treated. In some cases, however, the cancer may be found in the neck lymph nodes first, and the primary site cannot be identified even after extensive testing. This situation is referred to as metastatic squamous cell carcinoma of the neck with occult primary, or "cancer of unknown primary (CUP)".  Treatment options depend on several factors, such as the location and size of the primary tumor (if it can be found), the number and size of the neck lymph nodes involved, the patient's overall health, and others. Treatments may include surgery, radiation therapy, chemotherapy, targeted therapy, immunotherapy, or a combination of these. Prognosis varies widely, depending on the same factors.  As of my knowledge cutoff in September 2021, much research is being done to improve the understanding, diagnosis, and treatment of this disease, so if you or a loved one is dealing with this condition, be sure to consult with a healthcare provider or cancer specialist who is up-to-date with the latest developments. | Comparable | 1 | 1 | Not applicable |
| 5. Do all types of head and neck cancer exhibit the same symptoms? | No, not all types of head and neck cancer exhibit the same symptoms. The specific symptoms of head and neck cancers can vary significantly depending on the location and stage of the tumor. Head and neck cancers include a variety of conditions affecting areas such as the mouth, nose, throat, larynx (voice box), salivary glands, and lymph nodes in the neck.  Here are some possible symptoms based on the type of head and neck cancer:   1. Oral (mouth) cancer: Symptoms can include sores that don't heal, red or white patches on the gums or tongue, unexplained bleeding in the mouth, ongoing pain or discomfort, difficulty or discomfort when swallowing, changes in speech, or a lump in the neck. 2. Pharyngeal (throat) cancer: Symptoms might include trouble swallowing, a sore throat that does not go away, persistent cough, weight loss, voice changes, ear pain, or a lump in the back of the mouth, throat or neck. 3. Laryngeal (voice box) cancer: Symptoms could be a persistent cough, changes in your voice such as hoarseness or not speaking clearly, difficulty swallowing, ear pain, trouble breathing, or a lump in the neck. 4. Nasal cavity and sinus cancer: Symptoms may include blocked sinuses that do not clear, chronic sinus infections that do not respond to treatment with antibiotics, nosebleeds, frequent headaches, swelling or other trouble with the eyes, pain in the upper teeth, or problems with dentures. 5. Salivary gland cancer: Symptoms may include a lump or swelling in your mouth, cheek, jaw, or neck, numbness in part of your face, muscle weakness on one side of your face, persistent pain in the area of a salivary gland, or trouble swallowing or opening your mouth widely. 6. Neck cancer (typically squamous cell carcinoma or lymphoma): This can present with a lump or mass in the neck, which may grow rapidly. Symptoms may also include pain, skin changes, or difficulty swallowing.   These are just some general symptoms and not everyone with head and neck cancer will experience all of these. If you or someone else has any of these symptoms, especially if they last for a while or are getting worse, it's important to see a healthcare provider to find out what the problem is and, if necessary, begin treatment as soon as possible. Please remember that many of these symptoms can also be caused by conditions that are not cancer, such as an infection. Only a healthcare provider can make a diagnosis. | Comparable | 1 | 1 | Not applicable |
| 6. What is the difference between nasopharyngeal and hypopharyngeal cancer? | Nasopharyngeal cancer and hypopharyngeal cancer are two distinct types of head and neck cancers that develop in different regions of the throat. Here's a brief overview of each:   1. Nasopharyngeal Cancer: Nasopharyngeal cancer originates in the nasopharynx, which is the upper part of the throat located behind the nose and above the soft palate. The nasopharynx is the region where the nasal passages and the throat meet. This type of cancer is relatively rare in most parts of the world but is more prevalent in certain geographic areas, such as Southeast Asia.   Risk factors for nasopharyngeal cancer include a family history of the disease, infection with the Epstein-Barr virus (EBV), exposure to certain environmental factors, and consumption of certain preserved foods. Symptoms may include a persistent sore throat, difficulty swallowing, nasal congestion, blood in the saliva, neck lumps, and hearing problems.   1. Hypopharyngeal Cancer: Hypopharyngeal cancer, also known as laryngopharyngeal cancer, develops in the hypopharynx, which is the lower part of the throat, just above the esophagus and windpipe. This type of cancer is often associated with tobacco and alcohol use, as well as other risk factors like poor nutrition and exposure to certain chemicals or asbestos.   Symptoms of hypopharyngeal cancer may include a persistent sore throat, difficulty swallowing, voice changes (such as hoarseness), weight loss, ear pain, and enlarged lymph nodes in the neck.  While both types of cancer involve the pharynx, they occur in different regions and can have distinct risk factors. It's important to note that the treatment and prognosis for each cancer can vary, so it's crucial to consult with a medical professional for accurate diagnosis, staging, and appropriate treatment options | Comparable | 1 | 1 | Not applicable |
| 7.What types of cancer are categorized as head and neck cancers? | Head and neck cancers typically begin in the squamous cells that line the moist, mucosal surfaces inside the head and neck. These cancers can occur in several places:   1. Oral cavity: This includes your lips, the front two-thirds of your tongue, your gums, the lining inside your cheeks and lips, the bottom of your mouth under the tongue, the hard top of your mouth, and the small area behind the wisdom teeth. 2. Pharynx: The pharynx (throat) is a hollow tube about 5 inches long that starts behind the nose and leads to the esophagus. It has three parts: the nasopharynx (the upper part of the pharynx, behind the nose); the oropharynx (the middle part of the pharynx, including the soft palate, the base of the tongue, and the tonsils); the hypopharynx (the lower part of the pharynx). 3. Larynx: The larynx, also called the voice box, is a short passageway formed by cartilage just below the pharynx in the neck. The larynx contains the vocal cords. 4. Paranasal sinuses and nasal cavity: The paranasal sinuses are small hollow spaces in the bones of the head surrounding the nose. The nasal cavity is the hollow space inside the nose. 5. Salivary glands: These glands produce saliva, the fluid that keeps mucosal surfaces in the mouth and throat moist.   Each of these areas can develop different types of tumors, both benign (non-cancerous) and malignant (cancerous). Most head and neck cancers are squamous cell carcinomas, which are malignancies that arise in the lining of the mouth, nose and throat.  Risk factors for developing head and neck cancer include tobacco use, heavy alcohol use, infection with certain types of human papillomavirus (HPV), sun exposure, certain workplace exposures, and poor oral hygiene.  Symptoms often include a persistent sore throat, hoarseness, weight loss, and difficulty swallowing or breathing. However, these can also be signs of less serious conditions. If you have these symptoms, especially if they last for a long time or get worse, see a healthcare provider to find out if they are caused by cancer or by some other problem. Early detection and treatment can significantly improve the outcome. | Comparable | 2 | 2 | Not applicable |
| 8.What are the primary causes of head and neck cancers? | Head and neck cancers include a wide range of tumors that can develop in several areas of the head and neck, such as the oral cavity, throat (pharynx), voice box (larynx), nose and sinuses, and salivary glands. The exact causes of these cancers are not completely understood, but several risk factors have been identified:   1. Tobacco use: This is the most important risk factor for head and neck cancers, particularly those in the oral cavity, oropharynx, larynx, and hypopharynx. Both smoking cigarettes and using smokeless tobacco (like snuff or chewing tobacco) significantly increase risk. 2. Alcohol consumption: Frequent and heavy consumption of alcohol is a major risk factor, especially for cancers of the oral cavity, pharynx, and larynx. The risk is significantly higher for those who both smoke and drink heavily. 3. Human papillomavirus (HPV) infection: Certain types of HPV, particularly HPV type 16, have been linked to a subset of head and neck cancers, most notably oropharyngeal cancers. 4. Epstein-Barr virus (EBV) infection: EBV is associated with certain types of nasopharyngeal cancer and some types of salivary gland cancers. 5. Exposure to certain workplace hazards: Regular exposure to wood dust, asbestos, certain synthetic fibers, and certain heavy metals like nickel and chromium can increase the risk of some types of head and neck cancer. 6. Poor oral hygiene and missing teeth: These may be risk factors for oral and oropharyngeal cancers. 7. Diet: A diet low in fruits and vegetables, and potentially a diet high in processed foods or red meats, could increase the risk of head and neck cancers. 8. Genetic syndromes: People with certain inherited genetic conditions, like Fanconi anemia or dyskeratosis congenita, have a higher risk of developing head and neck cancer. 9. Radiation exposure: Exposure to radiation, particularly in the head and neck area, increases the risk of developing head and neck cancer. 10. Betel quid and gutka: In some parts of the world, especially South Asia, the habit of chewing betel quid and gutka is a major cause of oral and oropharyngeal cancer.   Remember, having one or more risk factors doesn't necessarily mean that a person will develop head and neck cancer. Many people with these risk factors never develop the disease, and many who do develop it may have few or no known risk factors. | Comparable | 1 | 1 | Not applicable |
| 9.How common are head and neck cancers? | Head and neck cancers represent a group of cancers that typically start in the squamous cells lining the moist, mucosal surfaces inside the head and neck, such as inside the mouth, nose, and throat. As of my knowledge cutoff in September 2021, head and neck cancers accounted for about 4% of all cancers in the United States.  However, these statistics can vary worldwide, with a higher prevalence in certain regions due to lifestyle factors and risk factors, such as tobacco and alcohol use, certain types of human papillomavirus (HPV), and consumption of preserved or salted foods.  It's important to note that the prevalence of head and neck cancers, like any type of cancer, can change over time due to changes in lifestyle factors, improvements in detection and diagnosis, and other influences.  If you need the most recent statistics or more specific information, you should check with authoritative sources like the American Cancer Society, World Health Organization, or the National Cancer Institute, as they regularly update their data.  Moreover, always seek professional medical advice for health-related concerns. | Comparable | 1 | 1 | Not applicable |
| 10. Can frequent sinus infections be an indicator of paranasal sinus sancer? | Sinus infections, also known as sinusitis, are quite common and generally not linked to paranasal sinus cancer. However, persistent symptoms that do not respond to treatment may warrant further investigation.  Paranasal sinus cancer is quite rare. Signs and symptoms of this cancer can include:   1. Persistent sinus congestion and sinusitis that doesn't improve with treatment 2. Nasal obstruction or persistent nasal stuffiness 3. Frequent nosebleeds 4. Decreased sense of smell 5. Pain in the face, upper teeth or ears 6. Persistent sore throat or difficulty swallowing 7. Swelling or other trouble with the eyes, such as double vision 8. Persistent or recurrent headaches   Keep in mind that these symptoms can also be due to non-cancerous conditions such as sinusitis, nasal polyps, or other less serious issues. The important factor is persistence - if these symptoms do not respond to normal treatments, or if they persist for a long time, then further investigation is warranted.  It's always best to consult with a healthcare provider if you have any concerns about your health. They can guide you on the best course of action based on your individual situation and symptoms. | Comparable | 1 | 1 | Not applicable |
| 11. To which regions does metastatic throat cancer most commonly spread? | Metastatic throat cancer, like many cancers, typically spreads first to the closest or most easily accessible areas. This usually includes:   1. Lymph nodes: The lymphatic system is often the first place where throat cancer spreads, because it is a network of organs and tissues that help rid the body of toxins, waste, and other unwanted materials. The spread of cancer to lymph nodes is called lymph node involvement or lymphatic spread. 2. Lungs: The lungs are one of the most common sites for metastasis for most types of cancer, including throat cancer. This is in part because the body's circulatory system, which the cancer cells can use to travel, passes through the lungs. 3. Bones: Especially in advanced cases, throat cancer may spread to bones. This is more common with certain types of cancer than others. 4. Liver: The liver is also a common site of metastasis for many types of cancer, including throat cancer. 5. Brain: Although it is less common, throat cancer can spread to the brain, especially in advanced cases.   Note that the specific pattern of spread can depend on the exact type and stage of the throat cancer, among other factors. These are general trends and not hard-and-fast rules. For a more accurate prognosis and information, it's always best to consult with an oncologist or other medical professional. | Comparable | 2 | 2 | Not applicable |
| 12.What is head and neck cancer of unknown primary site? | Head and neck cancer of unknown primary site, also referred to as CUP (Cancer of Unknown Primary), is a condition in which malignant (cancer) cells are found in the head and neck, but the place the cancer first began (the primary site) cannot be determined.  The cells have the characteristics of head and neck cancer cells, but even after extensive testing and evaluation, the doctors can't locate where the cancer originated.  This is unusual because cancer cells usually resemble the cells of the tissue in which the cancer began. For example, if breast cancer spreads to the lungs, the cancer cells in the lungs look like breast cancer cells, not like lung cancer cells.  When cancer of unknown primary site is diagnosed, it has already metastasized (spread) from the place where it started to another part of the body. The place where the cancer started is called the primary site, and the cancer is usually named after the primary site, no matter where else in the body it spreads. When the primary site cannot be found, it is called a cancer of unknown primary site.  The prognosis and treatment for head and neck cancer of unknown primary site can be quite different from other more typical head and neck cancers, and may depend on many factors, including the patient's overall health, the specific location and extent of the disease, and the characteristics of the cancer cells themselves. Treatment may include radiation therapy, chemotherapy, surgery, or a combination of these. | Comparable | 2 | 2 | Not applicable |
| 13.Is it possible to cure head and neck cancer? | Head and neck cancers, which include cancers of the mouth, throat, nasal cavity, sinuses, salivary glands, and lymph nodes in the neck, are potentially curable, especially if they are detected early. The success rate of treatment varies greatly and depends on a number of factors including the type, location, and stage of the cancer, as well as the patient’s overall health.  Treatment options for head and neck cancers include surgery, radiation therapy, chemotherapy, targeted therapy, or a combination of these. The choice of treatment depends on the stage of the cancer, its location, the patient’s overall health, and the potential side effects of the treatment. The goal of treatment can be curative (to completely eliminate the cancer), control (to slow down or stop the growth or spread of the cancer), or palliative (to relieve symptoms and improve quality of life).  In some cases, early-stage head and neck cancers can be treated successfully with surgery or radiation therapy alone. Advanced-stage cancers, however, might require a combination of treatments. The overall 5-year survival rate for all types of head and neck cancer is around 60-65%, but this rate can be much higher or lower depending on the specific type and stage of cancer, among other factors.  Follow-up care is crucial for any cancer survivor, as it allows doctors to keep track of the patient’s recovery, manage any side effects of treatment, and Profess for any signs of recurrence. Lifestyle changes, such as quitting smoking or alcohol, can also significantly reduce the risk of recurrence.  Please note that while this information is based on current understandings of head and neck cancers, actual treatments and prognosis may vary based on advancements in medical technology and an individual’s unique case. Profess most accurate information, it is best to consult with a medical Professional. | Comparable | 1 | 1 | Not applicable |
| 14.What is HPV p16 positive head and neck cancer? | Head and neck cancer refers to a group of cancers that start in the mouth, nose, throat, larynx, sinuses, or salivary glands. The most common type of head and neck cancer is squamous cell carcinoma, which begins in the squamous cells that line the moist, mucosal surfaces inside the head and neck.  One of the risk factors for developing head and neck cancer is infection with human papillomavirus (HPV), a common sexually transmitted infection. Certain strains of HPV, particularly HPV type 16, are associated with a higher risk of certain types of cancer, including oropharyngeal cancer, a type of head and neck cancer that primarily affects the tonsils and the base of the tongue.  When a patient is diagnosed with HPV-related head and neck cancer, their tumor is often tested for the presence of p16, a protein that's overexpressed in cells that have been transformed by HPV. p16 is used as a surrogate marker for HPV infection. So, when a patient's cancer is "p16 positive," it's likely that their cancer was caused by an HPV infection.  The importance of this distinction is that HPV-related head and neck cancers tend to respond better to treatment and have better survival rates than those that are not related to HPV. Patients with HPV-positive oropharyngeal cancer, despite often being diagnosed at an advanced stage, tend to have a better prognosis than patients with HPV-negative cancer. This has led to ongoing research to see if treatment can be de-intensified for these patients, to reduce the long-term side effects of treatment without compromising its effectiveness. As of my knowledge cutoff in September 2021, this research was ongoing, and patients should discuss their treatment options with their healthcare team. | Comparable | 1 | 1 | Not applicable |
| 15.What is the precise difference between T3 and T4 larynx cancer? | In the context of laryngeal cancer, the T in the TNM (Tumor, Node, Metastasis) staging system refers to the size and/or extent of the primary tumor. According to the American Joint Committee on Cancer (AJCC) 8th edition, which is the latest as of my last training data in September 2021, the T3 and T4 stages for laryngeal cancer are generally defined as follows:   - T3: Tumor is limited to larynx with vocal cord fixation (the vocal cord can't move as it should), and/or invades the paraglottic space, and/or with minor thyroid cartilage erosion (erosion in the inner cortex). - T4: This stage is subdivided into T4a and T4b.   - T4a (moderately advanced local disease): Tumor invades through the thyroid cartilage and/or invades tissues beyond the larynx such as the trachea, soft tissues of neck, thyroid, and/or esophagus.   - T4b (very advanced local disease): Tumor invades prevertebral space, encases carotid artery, or involves mediastinal structures.   As mentioned previously, these definitions might change in future editions of the AJCC Cancer Staging Manual due to advances in understanding and treatment of the disease. For the most accurate staging information, it's always advisable to refer to the most current manual or consult with a healthcare provider.  The T stage is combined with the N stage (extent of spread to lymph nodes) and M stage (presence of distant metastasis) to determine the overall stage of the cancer. This overall stage, in conjunction with other factors such as the type and grade of the cancer and the patient's overall health, helps guide treatment decisions and prognosis. | Comparable | 2 | 2 | Not applicable |
| 16.I have a 5-mm squamous cell tumor in the pyriform sinus with no local or distant spread. What is the average survival rate? | I'm an AI and I can provide general information based on existing knowledge up until September 2021. Survival rates can depend on many factors, such as the stage of the cancer at the time of diagnosis, the individual's overall health, the specific characteristics of the cancer, and how well the cancer responds to treatment.  Cancer is typically staged from 0 to 4, with Stage 0 being in situ (localized within the original location) and Stage 4 being advanced with distant spread (metastasis). A 5-mm squamous cell carcinoma in the pyriform sinus without any local or distant spread would likely be considered in the early stages.  For head and neck cancers, which include cancers of the hypopharynx where the pyriform sinus is located, the 5-year survival rate for localized (Stage I and some Stage II) disease is generally around 83%. However, this figure is not specific to squamous cell carcinoma of the pyriform sinus, as it includes all types of head and neck cancers.  For a more accurate prognosis, please consult your healthcare provider. They have the most complete understanding of your personal health history and current situation, and can provide you with the most precise information. | Comparable | 1 | 1 | Not applicable |
| 17. What is the precise difference between T2 and T3 tongue cancer? | In the TNM staging system for oral cavity cancers, including cancers of the tongue, the "T" stands for "Tumor," and the number indicates the size and extent of the primary tumor. The specific criteria can vary between different types of cancer, but generally:   - T2: In the context of oral tongue cancer, a T2 tumor is one that is more than 2 cm but not more than 4 cm at its greatest dimension. - T3: A T3 oral tongue cancer is typically a tumor that is more than 4 cm in greatest dimension.   These criteria come from the American Joint Committee on Cancer (AJCC) 8th edition, which was the latest edition as of my training data cut-off in September 2021. This staging system is subject to updates and revisions, so the exact definitions might change in future editions of the manual. Always refer to the most current manual or consult with a medical professional for the most accurate staging information.  It's also important to note that TNM staging is just one part of cancer classification. The size of the tumor (T), the extent of spread to the lymph nodes (N), and the presence of distant metastasis (M) are all considered together to determine the overall stage of the cancer (from stage I to stage IV). Other factors, such as the histological type and grade of the cancer, the patient's overall health and preferences, and other considerations, also play a role in planning treatment and predicting outcomes. | Comparable | 2 | 2 | Not applicable |
| 18. Is tracheotomy always mandatory before cordotomy for bilateral vocal fold paralysis due to radiotherapy? | Bilateral vocal fold paralysis (BVFP) is a condition where both vocal cords are immobile and often trapped in a midline or nearly midline position, blocking the airway and making it difficult to breathe. It can be caused by a number of different factors, including nerve damage due to surgery, trauma, or in your case, radiotherapy.  Cordotomy, also known as vocal cordotomy or cordectomy, is a surgical procedure performed to improve breathing by removing a part or all of the vocal cord(s). This is typically considered for patients with BVFP if other therapies (like voice therapy or non-surgical interventions) haven't worked or are not suitable.  Whether a tracheotomy (surgical opening in the trachea or windpipe) is needed prior to cordotomy will largely depend on the patient's condition and the surgeon's assessment. Tracheotomy can provide an alternative breathing route, bypassing the blocked upper airway, which could be beneficial during recovery from cordotomy.  However, the necessity of tracheotomy is not always mandatory. Tracheotomy can carry its own risks and complications, and in some cases, doctors may perform cordotomy without prior tracheotomy, particularly if the patient's airway obstruction isn't severe or if other mitigating strategies can be used. Furthermore, technological advances in anesthesiology and surgical methods may allow some patients to undergo cordotomy without a prior tracheotomy.  Ultimately, the decision is very case-specific, taking into consideration the patient's overall health, the severity of the airway obstruction, the risks and benefits of each procedure, and the surgeon's experience and comfort with each procedure. It's always recommended to have a detailed discussion with your healthcare team to understand the most suitable approach. | Comparable | 1 | 1 | Not applicable |
| 19. Is head and neck cancer more common in males or females? | Head and neck cancers have traditionally been more common in males than females. This is due to higher rates of risk factors such as tobacco and alcohol use in men, which significantly contribute to the development of these cancers. However, human papillomavirus (HPV) related oropharyngeal cancers are becoming increasingly common in both men and women.  It is important to keep in mind that this generalization is based on data up to my knowledge cut-off in September 2021. The prevalence of head and neck cancers could vary depending on changes in risk factors, such as smoking rates, alcohol consumption, and HPV infection, in different populations over time.  Always remember to regularly check up-to-date sources for the most current information, and consult with healthcare professionals for medical advice. | Comparable | 1 | 1 | Not applicable |
| 20. What is the current rate of HPV positivity in oropharynx cancer? | As an AI, I don't have real-time or post-September 2021 data access to provide the current rate of HPV positivity in oropharynx cancer. However, as of my last update, a significant proportion of oropharyngeal cancers were associated with HPV. This figure is widely variable, but studies often indicate that around 60-70% of oropharyngeal cancers in the United States are linked to HPV, specifically HPV type 16.  This trend has been increasing over the past several decades, particularly in men, which has led to changes in the epidemiology of head and neck cancers.  I'd recommend checking the latest statistics from cancer registries or health organizations like the American Cancer Society, the Centers for Disease Control and Prevention (CDC), or the World Health Organization (WHO) for the most current and accurate information. | Comparable | 1 | 1 | Not applicable |
| 21. Does a family history of oral, throat, or tongue cancer increase my risk for head and neck cancer? | Yes, a family history of oral, throat, or tongue cancer can increase your risk for head and neck cancer. However, this does not mean that if a close relative had one of these cancers that you will necessarily develop it, just that your risk may be higher than for those without such a family history.  Several factors contribute to the risk of developing head and neck cancers:   1. Tobacco and Alcohol Use: The most significant risk factors for head and neck cancers are tobacco and alcohol use. These cancers are often linked to tobacco use, including cigarettes, cigars, pipes, and smokeless tobacco. High alcohol consumption, particularly in conjunction with tobacco use, also significantly increases risk. 2. Certain Infections: Human papillomavirus (HPV) infection, particularly HPV16, is a risk factor for some types of head and neck cancers, especially oropharyngeal cancers. Infections with Epstein-Barr virus (EBV), which causes mononucleosis, are also linked with certain types of head and neck cancers. 3. Age and Gender: Head and neck cancers are more common in older adults and in men. 4. Occupational Exposure: Certain industries, like woodworking and metalworking, can increase a person's risk due to exposure to certain chemicals or dusts. 5. Poor Nutrition: A diet low in vitamins A and B can increase the risk of head and neck cancers. 6. Genetics: Having a family history of head and neck cancer can increase your risk. Certain inherited genetic syndromes can also increase the risk of head and neck cancers, like Fanconi anemia and dyskeratosis congenita.   It's important to note that having a risk factor does not mean you will get the disease, and not having a risk factor doesn't mean you won't get it. However, if you're concerned about your risk of head and neck cancers due to your family history, you should discuss this with your doctor. They may recommend more frequent screenings or other preventative measures based on your personal and family medical history. | Comparable | 1 | 1 | Not applicable |
| 22. Which ethnic groups are at higher risk for head and neck cancer? | As of my knowledge cutoff in September 2021, several studies have indicated that race and ethnicity may influence the risk of head and neck cancers. Here are some general trends observed:   1. African American population: Research indicates that African American men are at a higher risk for developing head and neck cancer, particularly cancers of the larynx. Additionally, they often have worse outcomes, which may be related to factors like later-stage diagnosis, differential access to care, and potential biological differences in tumor behavior. 2. White population: White men have a higher rate of oral cavity and oropharyngeal cancers, largely attributed to higher rates of alcohol and tobacco use, and increasing rates of human papillomavirus (HPV)-related oropharyngeal cancer. 3. Asian population: Certain types of head and neck cancers, such as nasopharyngeal cancer, are more common in people from East Asia (including Southern China and Taiwan) and North Africa. This is potentially linked to genetic factors, dietary factors (like consumption of certain preserved foods), and Epstein-Barr virus infection. 4. South Asian population: In India and other South Asian countries, high rates of oral and oropharyngeal cancers are observed, largely due to widespread use of chewable tobacco and betel quid.   Remember, these are broad generalizations and may not apply to every individual within these racial and ethnic groups. Other factors like lifestyle habits, socio-economic status, and access to healthcare play significant roles in cancer risk and outcomes. It's also important to note that the understanding of cancer disparities is continually evolving, and newer research may provide additional insights.  For the most accurate and up-to-date information, it is best to consult with healthcare providers or trusted health information sources. | Comparable | 1 | 1 | Not applicable |
| 23. Does exposure to secondhand smoke increase the risk for head and neck cancer? | Yes, exposure to secondhand smoke can increase the risk of various types of cancer, including head and neck cancer. Secondhand smoke contains many of the same harmful chemicals that smokers inhale. These chemicals can damage cells in the body, leading to cancer development.  Research has shown that non-smokers exposed to secondhand smoke at home or work have a higher risk of developing head and neck cancer. The risk can increase depending on the duration and intensity of exposure to secondhand smoke. Other factors, such as alcohol consumption, can also increase this risk.  Remember, the best way to prevent exposure to secondhand smoke is by enforcing smoke-free environments in homes, workplaces, and public places. It's also crucial to get regular medical check-ups to detect any potential health problems early. | Comparable | 1 | 2 | 1 |
| 24. Can barbecue grilling lead to head and neck cancer? | As of my knowledge cutoff in September 2021, no specific study has established a direct link between barbecue grilling and head and neck cancer. However, a body of research has suggested that eating charred, well-done meat cooked at high temperatures, such as what can occur with barbecue grilling, may be associated with an increased risk of certain types of cancer, including colorectal, pancreatic, and prostate cancers. This is believed to be due to the formation of potentially carcinogenic compounds known as heterocyclic amines (HCAs) and polycyclic aromatic hydrocarbons (PAHs) in meat cooked at high temperatures.  However, it's important to note that a multitude of factors contribute to cancer risk, including genetic predisposition, lifestyle habits (such as tobacco and alcohol use), and exposure to certain types of infections (like HPV for head and neck cancers).  The overall risk related to eating grilled meat is considered to be relatively small, and it can be further reduced by taking some precautions like marinating the meat, avoiding charring or burning the meat, flipping it frequently, and not consuming the blackened parts. These methods can help minimize the formation of HCAs and PAHs.  If you're worried about cancer risks associated with grilling, you should discuss your concerns with a healthcare provider who can provide the most current and personalized advice based on the latest research and your individual health situation.  Remember that this information is accurate as of 2021, and new research could provide updated information on the potential risks. Always look for the latest and most credible sources when researching health topics. | Comparable | 1 | 1 | Not applicable |
| 25. Can consuming hot drinks cause head and neck cancer? | There has been some controversy and confusion about this topic, and it's important to clarify that it is not the hot drink itself that is thought to be potentially harmful, but the temperature at which it is consumed.  The World Health Organization's International Agency for Research on Cancer (IARC) has stated that drinking beverages at a very high temperature, above 65 degrees Celsius (149 degrees Fahrenheit), may possibly lead to esophageal cancer, which is a type of cancer located in the neck region.  The reasoning is that chronic injury to the throat due to consuming very hot beverages could lead to inflammation and long-term damage that could eventually increase the risk of developing cancer.  However, it's important to note that this risk is generally considered relatively small compared to other risk factors for esophageal cancer such as heavy alcohol consumption, smoking, obesity, and certain dietary factors.  Also, most people don't usually consume their beverages at such high temperatures. For example, coffee is typically served at temperatures below 65 degrees Celsius (149 degrees Fahrenheit).  So, while there might be a small risk associated with consuming very hot drinks, it is only one of many potential factors and not the most significant one. If you enjoy hot beverages, just make sure they are not scalding hot.  As always, for personal medical advice, it's best to consult with a healthcare provider. | Comparable | 2 | 1 | 1 |
| 26. I have white patches in my mouth. Does this put me at risk for oral cancer? | White patches in the mouth, known as leukoplakia, can be caused by a variety of factors, including tobacco use, alcohol consumption, and irritation from dental appliances. While leukoplakia can sometimes be a precursor to oral cancer, most instances of leukoplakia do not lead to cancer.  There are some cases, though, where leukoplakia may be more concerning. If the patches are hard, cannot be scraped off, are combined with red areas (known as erythroplakia), or are associated with a change in the texture or feeling in the mouth, there may be a higher risk of cancer.  However, it's important to remember that many oral conditions can cause white patches in the mouth, including thrush (a fungal infection), lichen planus (a type of inflammatory response), and some other less common conditions.  It's very important that you consult a healthcare professional if you notice these or any other changes in your mouth, even if they don't cause pain. A healthcare professional can examine the white patches and potentially perform other diagnostic tests, like a biopsy, to determine the cause and appropriate treatment.  This advice is intended to provide general information about potential causes of white patches in the mouth, and should not replace consultation with a healthcare provider. It is important to seek professional advice for any health concern. | Comparable | 1 | 1 | Not applicable |
| 27. I have been noticing blood in my saliva. Does this put me at risk for oral cancer? | Blood in your saliva could be due to many reasons, and while it could potentially be a symptom of oral cancer, it can also be associated with many other conditions that are far less severe, such as gum disease, a minor trauma to your mouth or throat, or a simple nosebleed that has drained into your throat.  Symptoms of oral cancer might include:   - A sore that doesn't heal - Loose teeth - Tongue pain - Jaw pain or stiffness - Difficulty or discomfort swallowing - Painful chewing - A change in your voice - Dramatic weight loss   Having just one of these symptoms, such as blood in your saliva, doesn't necessarily mean you have oral cancer. Many of these symptoms could be due to other conditions.  However, if you're noticing blood in your saliva consistently or you're experiencing other symptoms, it's important to seek medical attention promptly. It's always better to get checked out to ensure nothing serious is going on. It's also a good reminder of the importance of regular dental checkups, since dentists can often spot potential issues early, when they're easiest to treat. | Comparable | 1 | 2 | 2 |
| 28. What is the exact difference between N2 and N3 neck stage in oral cavity cancer? | Oral cavity cancer is typically staged using the American Joint Committee on Cancer (AJCC) TNM system. As of the 8th Edition, which was current as of my knowledge cutoff in September 2021, the definitions for N2 and N3 stages for oral cavity cancer are:   - N2 is subdivided into N2a, N2b, and N2c:   - N2a: Metastasis in a single ipsilateral (same side as the primary tumor) lymph node, more than 3 cm but not more than 6 cm in greatest dimension.   - N2b: Metastasis in multiple ipsilateral lymph nodes, none more than 6 cm in greatest dimension.   - N2c: Metastasis in bilateral (on both sides of the body) or contralateral (on the opposite side as the primary tumor) lymph nodes, none more than 6 cm in greatest dimension. - N3 also has two subdivisions:   - N3a: Metastasis in a lymph node larger than 6 cm in greatest dimension.   - N3b: Tumor has spread to a lymph node that is on, above, or below the collarbone.   This staging can influence the treatment strategy, which may include surgery, radiation therapy, and chemotherapy.  Again, please note that my information is based on data available up to September 2021. It's crucial to consult a medical professional or the most up-to-date resources for the most current and accurate staging definitions. | The staging of neck lymph node involvement in oral cavity cancer is an important factor in determining prognosis and guiding treatment. According to the American Joint Committee on Cancer (AJCC) 8th Edition Staging System (as of my knowledge cut-off in September 2021), the N stages of oral cavity cancer are as follows:   - N0: No regional lymph node metastasis. - N1: Metastasis in a single ipsilateral lymph node, 3 cm or less in greatest dimension. - N2: This is divided into three categories:   - N2a: Metastasis in a single ipsilateral lymph node, more than 3 cm but not more than 6 cm in greatest dimension.   - N2b: Metastasis in multiple ipsilateral lymph nodes, none more than 6 cm in greatest dimension.   - N2c: Metastasis in bilateral or contralateral lymph nodes, none more than 6 cm in greatest dimension. - N3: This is divided into two categories:   - N3a: Metastasis in a lymph node more than 6 cm in greatest dimension.   - N3b: Metastasis in any lymph node(s) along the lower jugular chain or supraclavicular fossa.   The main differences between N2 and N3 stages, therefore, are related to the size of the involved lymph nodes and their location. The N2 stage reflects involvement of a single or multiple nodes, none more than 6 cm, whereas the N3 stage indicates the involvement of a lymph node larger than 6 cm (N3a) or nodes located along the lower jugular chain or supraclavicular fossa (N3b).  Please note, guidelines may be updated, and management approaches may vary among institutions and physicians. Thus, it's always crucial to consult with the treating oncologist for the most current and individualized information. | 3,3 | 2,2 | 3,3 |
| 29. Can a surgery or biopsy for head and neck cancer cause the cancer to spread throughout the body? | There's a common myth that surgery can cause cancer to spread in the body. It is important to clarify that the risk of cancer spreading due to surgery is extremely low. When surgeons operate to remove a tumor, they take several precautions to prevent the spread of cancer cells.  Firstly, they attempt to remove the entire tumor, along with a margin of normal tissue surrounding it, to ensure no cancer cells are left behind. The removed tissue is then sent to a pathology lab to confirm that the margins are clear of cancer cells.  Secondly, in the case of head and neck cancers, if there's a risk of cancer cells having spread to nearby lymph nodes, surgeons might also perform a procedure called a lymph node dissection to remove those lymph nodes. This is done to prevent the further spread of cancer.  Finally, surgeons use methods to minimize the chance of cancer cells breaking away during surgery. For example, they may use special techniques to contain the tumor and its surrounding tissue.  However, it's worth noting that if a cancer is aggressive or has already spread at the time of surgery, there may be microscopic cells in other parts of the body that surgery won't remove. This is why surgery is often combined with other treatments like radiation therapy or chemotherapy, which can kill these scattered cancer cells.  As for biopsies, while it theoretically could happen, the risk of spreading cancer due to a biopsy is also extremely low. Biopsies are done using a very fine needle, and the risk of dislodging a cancer cell that then travels through the bloodstream to another part of the body (a process known as metastasis) is very minimal. Biopsies are crucial for diagnosing cancer and determining its stage and grade.  In summary, while no procedure is without risks, the risk of surgery or a biopsy causing cancer to spread is low. These are standard, necessary procedures in diagnosing and treating cancer, and their benefits far outweigh the risks in most cases.  You should always discuss these concerns with your medical team who can give you advice based on your specific situation and the most recent scientific evidence. They can explain the risks and benefits of your treatment options so that you can make an informed decision about your care. | Comparable | 1 | 1 | Not applicable |
| 30. I experience rapid swelling below my jaw when chewing, which disappears gradually over time. Could this be a sign of cancer? | Rapid swelling under your jaw while chewing could be a symptom of various conditions, but it's not necessarily a sign of cancer. One common cause of this symptom could be salivary gland disorders, such as sialadenitis (infection of a salivary gland), salivary stones (sialolithiasis), or Sjogren's syndrome, which affects the salivary and tear glands.  In salivary gland disorders, you might feel pain and swelling in the salivary glands (around your mouth and in parts of your face) when you start to eat. The pain can gradually diminish as you continue to eat.  Although the symptom you described isn't a definitive sign of cancer, it's important to seek medical advice if you notice changes like this. There are types of cancer that can affect the salivary glands, but these are relatively rare. Other signs of salivary gland cancer can include a lump or swelling on or near your jaw or in your neck or mouth, numbness in part of your face, or muscle weakness on one side of your face.  Please note that the information provided here should not be used as a substitute for professional medical advice. If you're experiencing this symptom, it's essential to consult with a healthcare professional who can evaluate your condition properly. | Comparable | 1 | 1 | Not applicable |
| 31. I have had an ulcer on my lip for 3 months. What are the indications for a biopsy? | Oral ulcers (sores) that don't heal within about two weeks are considered abnormal and should be evaluated by a healthcare professional. A non-healing ulcer lasting for 3 months is highly unusual and should definitely be checked out.  A biopsy may be considered in the following circumstances:   1. Long Duration: If an oral lesion or ulcer has been present for more than 2-3 weeks without signs of healing, this is a common indication for a biopsy. 2. Change in Characteristics: Any change in color, size, shape, or if the ulcer begins to bleed or cause pain. 3. Previous Oral Diseases or Conditions: People who have a history of oral diseases or conditions, such as oral cancer or pre-cancerous lesions, may need a biopsy if they develop new or recurring ulcers or lesions. 4. Risk Factors: Those who have significant risk factors for oral cancer, such as heavy smoking or alcohol use, might be more likely to need a biopsy for a persistent oral ulcer.   In your case, it's crucial that you seek medical attention as soon as possible. The presence of an ulcer for 3 months is a concerning sign, and it's important to rule out serious conditions, such as oral cancer. Please make an appointment with a healthcare provider who can examine the ulcer and decide on the best course of action. | Comparable | 1 | 1 | Not applicable |
| 32. What does in-situ head and neck carcinoma mean? | In-situ" carcinoma refers to a stage of cancer where the disease is still localized to the layer of cells where it started and hasn't spread to deeper layers or to other parts of the body. So, "in-situ head and neck carcinoma" would be cancer that has originated in the head or neck region (like the oral cavity, pharynx, larynx, paranasal sinuses, nasal cavity, or salivary glands) and is still contained to its place of origin.  There are many types of head and neck carcinomas, including squamous cell carcinomas, adenocarcinomas, and others. In-situ carcinomas, regardless of the type, are usually treated with the aim of complete removal or destruction of the cancerous cells. Treatment can include surgery, radiation therapy, chemotherapy, or a combination of these modalities. The specific treatment approach depends on several factors such as the exact location of the cancer, the type of cancer, and the patient's overall health.  Carcinoma in situ is often highly treatable because it has not yet invaded beyond its original site. However, it can progress into invasive cancer if left untreated. So, early detection and treatment are important.  As always, a doctor or healthcare professional should be consulted for any health-related concerns or questions. They will be able to provide the most accurate and updated information. | Comparable | 1 | 1 | Not applicable |
| 33. Is head and neck cancer contagious? | No, head and neck cancer is not contagious. Cancer is a class of diseases characterized by out-of-control cell growth. It starts when cells in a part of the body start to grow out of control. Cancer is always caused by changes to the DNA within cells.  Cancer is generally caused by a combination of factors, including genetic, environmental, and lifestyle factors, such as smoking or heavy alcohol use, especially for head and neck cancers. Some types of head and neck cancer, particularly those involving the tonsils or base of the tongue, are associated with human papillomavirus (HPV), which is a sexually transmitted infection. However, it's the virus that is contagious, not the cancer itself.  So, while you can contract HPV from someone else, you cannot catch cancer from another person. Instead, you might develop cancer if you are infected with a high-risk type of HPV, and the virus causes changes to your cells that lead them to start growing uncontrollably. But again, this is a long process that often takes years, and not everyone who gets HPV will develop cancer. | Comparable | 1 | 1 | Not applicable |
| 34. My biopsy report shows mild dysplasia on my vocal cord. Does this pose a risk for larynx cancer? | Dysplasia is a term used in pathology to refer to an abnormality in maturation of cells within a tissue. This can result in cells that vary in size, shape, and organization. While this is generally not cancer, dysplasia can be precancerous and could progress to cancer if left untreated.  In the case of the larynx (voice box), mild dysplasia means that the cells of your vocal cords are starting to show changes. These changes can, in some cases, progress to a more severe form of dysplasia or even to laryngeal cancer. However, this is not always the case, and many instances of dysplasia may not progress at all.  The risk of progression from mild dysplasia to cancer is not well-defined as it varies depending on a number of factors including the individual's overall health, immune status, lifestyle factors like smoking and alcohol use, and possibly the presence of certain strains of the human papillomavirus (HPV).  It's very important to have regular follow-ups with your healthcare provider to monitor these changes. They might also recommend certain treatments or lifestyle changes to manage the condition. Always communicate with your doctor about your concerns and make sure you understand their recommendations for management and follow-up. | Comparable | 1 | 1 | Not applicable |
| 35. I have had an aphtous ulcer on my tonsil for 2 months. Does this put me at risk for cancer? | Aphthous ulcers, also known as canker sores, are usually harmless and often resolve on their own within 1-2 weeks. The fact that your ulcer has been present for 2 months is unusual and indicates that it might not be a simple canker sore.  While it's true that some forms of oral cancer can initially present as a non-healing ulcer, other more benign conditions such as viral or bacterial infections, immune-mediated diseases, or chronic trauma could also potentially cause a long-lasting oral ulcer.  It's important that you seek medical attention if you have a sore in your mouth that has not healed within two weeks. An evaluation from a healthcare professional, which might include a physical examination and potentially a biopsy, is the most effective way to rule out serious conditions, including oral cancer. | Comparable | 1 | 2 | 1 |
| 36. Can vaping lead to head and neck cancer? | As of my knowledge cut-off in September 2021, it's not definitively established whether vaping can directly cause head and neck cancers. However, some studies have suggested that vaping might be associated with an increased risk of certain types of cancer, including those of the head and neck, due to the presence of toxic and carcinogenic substances in e-cigarette vapor.  Tobacco smoking is a well-established risk factor for head and neck cancers, but e-cigarettes are a relatively new phenomenon, and more research is needed to definitively determine their long-term health impacts. The safety profile of e-cigarettes is not fully known, and while they are often marketed as a safer alternative to traditional cigarettes, they are not without risks.  Even though e-cigarettes typically contain fewer harmful chemicals than traditional cigarettes, they often still deliver nicotine, which is an addictive substance. Some studies have also found potentially harmful chemicals in e-cigarette vapor, including substances that are known to be toxic or carcinogenic. This includes formaldehyde, acrolein, and tiny particles that can be inhaled deep into the lungs.  Furthermore, the effects of e-cigarettes can vary widely depending on the specific product, how it's used, and the individual user's health status.  So, while it's not definitively established whether vaping can cause head and neck cancers, it's clear that vaping is not risk-free, and it's possible that future research may reveal more about its potential links to these and other health problems. It is always recommended to consult with a healthcare provider for personalized medical advice. | Comparable | 1 | 1 | Not applicable |
| ***DIAGNOSIS*** |  |  |  |  |  |
| 37. What are the most common symptoms of nasopharyngeal cancer? | Nasopharyngeal cancer refers to cancer that occurs in the nasopharynx, the upper part of the throat behind the nose and near the base of the skull.  Common symptoms of nasopharyngeal cancer can include:   1. Nosebleeds: Unexplained, frequent nosebleeds can be a symptom of nasopharyngeal cancer. 2. Nasal congestion or stuffiness: Difficulty breathing through the nose, especially if it's only on one side, can be a symptom. 3. Hearing loss: This can often be in one ear and might be accompanied by a ringing in the ear (tinnitus). 4. Ear infections: Frequent ear infections that don't respond to treatment can be a symptom. 5. Sore throat or difficulty swallowing: You might experience pain or discomfort in the throat, or difficulty swallowing. 6. Headaches: Regular, persistent headaches could be a sign of nasopharyngeal cancer. 7. Double vision or other vision problems: These might be caused by the cancer pressing on nerves. 8. Neck lump: A lump or swelling in the neck due to enlarged lymph nodes is a common symptom. 9. Facial pain or numbness: This can be caused by the cancer spreading to nerves in the face. 10. Change in voice or speaking: A hoarse voice or difficulty speaking could be a symptom. 11. Unexplained weight loss 12. Fatigue or feeling of unwell   These symptoms can also be caused by conditions other than nasopharyngeal cancer. If you have any of these symptoms and they last for more than a few weeks, or they're causing you concern, you should contact a healthcare provider for further evaluation. | Comparable | 1 | 1 | Not applicable |
| 38. Which doctor should I consult if I have a lump on my neck? | A lump on your neck could be a symptom of various conditions, ranging from benign (non-cancerous) like a lipoma or a swollen lymph node due to an infection, to serious conditions like cancer. It is important to get any new or changing lumps checked by a healthcare professional.  Start with a visit to a primary care physician (like a family doctor or internal medicine doctor) who can evaluate the lump. They might refer you to a specialist depending on the characteristics of the lump, your symptoms, and their initial assessment.  Possible specialists could include:   1. An Ear, Nose, and Throat (ENT) specialist, also known as an Otolaryngologist, who can examine lumps related to structures in the head and neck region. 2. A Hematologist, who specializes in conditions related to the blood and lymphatic system. If your lump is a swollen lymph node, a hematologist may be involved in your care. 3. An Endocrinologist, if the lump is suspected to be related to your thyroid gland, as these specialists handle thyroid conditions. 4. A Dermatologist might be consulted if the lump is on the skin or just underneath it and might be a skin condition. 5. A Surgeon or Surgical Oncologist if there is a need for a biopsy or removal of the lump.   Please consult with a healthcare professional promptly if you notice a lump on your neck, especially if it's new, changing, causing pain, associated with other symptoms, or if you're concerned in any way. It's essential to get a proper diagnosis for appropriate treatment. | Comparable | 1 | 1 | Not applicable |
| 39. Can oral cavity and oropharyngeal cancers be detected early? | Yes, oral cavity and oropharyngeal cancers can be found early in some cases, but it requires awareness of the symptoms and regular checkups, particularly for individuals at high risk.  Here are some ways to help find these types of cancer early:   1. **Regular dental checkups**: Dentists can often detect precancerous and cancerous lesions in their early stages during routine dental check-ups. It's generally recommended to visit the dentist at least once every six months. 2. **Self-examinations**: Regular self-examinations can help identify any changes in the mouth and throat, such as new lumps or sores that don't heal within two weeks. 3. **Screening**: Certain types of oral and oropharyngeal cancers can be detected through specific screening tests, though it's worth noting that as of my knowledge cut-off in September 2021, there's no standard or routine screening test for these types of cancer. 4. **Professional examinations**: Health professionals can do a more thorough examination of the mouth and throat as part of a routine check-up, particularly for individuals who use tobacco, drink alcohol heavily, or have other risk factors for these types of cancer.   Risk factors for oral cavity and oropharyngeal cancers include:   - Tobacco use, including smoking and smokeless tobacco - Heavy alcohol use - Infection with certain types of human papillomavirus (HPV) - Chronic inflammation and irritation - Certain genetic syndromes - A diet low in fruits and vegetables   If you're at high risk for these types of cancer or you have any symptoms such as a sore that doesn't heal, a lump or thickening in the cheek, a white or red patch on the gums, tongue, tonsil, or lining of the mouth, trouble swallowing or moving the tongue or jaw, a feeling that something is caught in the throat, or a change in voice, it's important to see a healthcare professional for an examination. | Comparable | 1 | 1 | Not applicable |
| 40. What are the signs and symptoms of oral cavity and oropharyngeal cancer? | Oral cavity and oropharyngeal cancers can present with several signs and symptoms, some of which are quite common and others that are more specific. However, it's important to remember that these symptoms can also be caused by conditions that are less serious than cancer, such as an infection. Here are some potential signs and symptoms:   1. A sore in the mouth that doesn't heal: This is the most common symptom. The sore can be on the lips, gums, or anywhere else inside the mouth. 2. Pain in the mouth: Any persistent mouth pain is a potential concern. 3. A lump or thickening in the cheek 4. A white or red patch on the gums, tongue, tonsil, or lining of the mouth 5. Difficulty swallowing or chewing 6. Difficulty moving the jaw or tongue 7. Numbness of the tongue or other areas of the mouth 8. Swelling of the jaw that causes dentures to fit poorly or become uncomfortable 9. Loose teeth or pain around the teeth or jaw 10. Voice changes 11. A sore throat or feeling that something is caught in the throat that doesn't go away 12. Trouble breathing or speaking 13. Persistent bad breath 14. Weight loss 15. Ear pain 16. A change in the way your teeth or dentures fit together   If you or someone else has any of these symptoms, particularly if they last for two weeks or longer, it's essential to see a healthcare professional. It's important to catch oral cancers as early as possible to improve the chances of successful treatment. | Comparable | 1 | 1 | Not applicable |
| 41. What diagnostic tests are available for oral cavity and oropharyngeal cancers? | Diagnosing oral cavity and oropharyngeal cancers typically involves several steps and tests. If a healthcare provider suspects these types of cancers, they may use the following diagnostic procedures:   1. Physical Examination: This involves an examination of the oral cavity and oropharynx (the back of the mouth and the throat). The doctor checks the mouth and throat for abnormalities, such as sores, white patches, or lumps. They also examine the neck for swollen lymph nodes, which may indicate the spread of cancer. 2. Endoscopy: A doctor may use a special lighted scope (endoscope) to examine hard-to-see areas of the mouth and throat. 3. Biopsy: In this procedure, a small sample of tissue is removed from the suspected area to be examined under a microscope by a pathologist. A biopsy is the most definitive way to diagnose cancer. Depending on the location of the suspected cancer, different types of biopsies might be used, including exfoliative cytology, incisional biopsy, and fine-needle aspiration biopsy. 4. Imaging Tests: These may include X-rays, computed tomography (CT) scans, magnetic resonance imaging (MRI) scans, and positron emission tomography (PET) scans. These tests help determine the size and location of the tumor and whether it has spread to other parts of the body. 5. Laboratory Tests: Lab tests, including blood and urine tests, can help rule out other diseases and also assess a patient's overall health, which can influence treatment options and prognosis. 6. HPV Testing: Some oropharyngeal cancers are associated with human papillomavirus (HPV) infection. Testing for HPV can provide important information about the cause of the cancer and can help guide treatment decisions. 7. Molecular Testing of the Tumor: Some tests can identify specific genes, proteins, and other factors unique to the tumor. Results of these tests can help determine your treatment options.   Remember, these tests are also used to stage the cancer, which helps guide treatment strategies. If you or someone else has symptoms that might indicate oral or oropharyngeal cancer, it's important to see a healthcare provider right away to determine the cause and begin appropriate treatment, if necessary. | Diagnosing oral cavity and oropharyngeal cancers typically involves a series of tests and evaluations. Here are some diagnostic tests commonly used:   1. Physical Examination: This is the first and foremost step where the doctor performs a detailed examination of the oral cavity and the throat. They check for any abnormalities such as lumps, discoloration, or ulcers. The doctor may use a small mirror and lights to visualize areas that are hard to see. 2. Endoscopy: For a closer look at the oral cavity and oropharynx, a doctor might use a thin, flexible tube with a light and a camera at its end, known as an endoscope. This helps the doctor view areas that aren't easily visible during a routine mouth or throat examination. 3. Biopsy: If any suspicious areas are identified during the physical exam or endoscopy, a biopsy may be performed. A sample of tissue is removed and sent to the lab where a pathologist examines it under a microscope to check for cancer cells. 4. Imaging Tests: Tests such as X-rays, Computerized Tomography (CT) scans, Magnetic Resonance Imaging (MRI), or Positron Emission Tomography (PET) scans may be used to determine the extent of the cancer, whether it has spread to nearby tissues or distant parts of the body (metastasized), and to help plan treatment. 5. HPV Testing: Since a large proportion of oropharyngeal cancers are associated with human papillomavirus (HPV), an HPV DNA test can be done on the cancerous tissue to detect the presence of virus. This can influence the prognosis and treatment plan. 6. Barium Swallow: This is a series of X-rays of your esophagus and stomach. You drink a liquid that contains barium (a silver-white metallic compound) to coat these areas. The coating allows these areas to show up more clearly on an X-ray. This can help determine if the cancer has spread to these areas. 7. Panendoscopy: This is a more comprehensive form of endoscopy that allows the doctor to check the throat, windpipe, and esophagus in detail. It's usually done under general anesthesia.   Please note that not all of these tests will be needed for every person, and some might be repeated during the course of diagnosis and treatment. The choice of diagnostic tests depends largely on the individual's symptoms, physical examination findings, and medical history. The doctor would provide guidance on which tests are most suitable for each individual. | 2,1 | 1,1 | 1,2 |
| 42. What are the signs and symptoms of sinonasal cancers? | Sinonasal cancers are rare and occur in the nasal cavity or the sinuses around the nose. They can be of different types such as squamous cell carcinoma, adenocarcinoma, melanoma, and others.  The signs and symptoms can vary depending on the exact location and size of the tumor, and whether it has spread to other parts. Common symptoms may include:   1. Nasal obstruction or persistent nasal congestion and stuffiness, which is often one-sided. 2. Frequent nosebleeds or unusual nasal discharge, which can be bloody. 3. Frequent sinus infections that do not respond to treatment with antibiotics. 4. Decreased sense of smell. 5. Numbness or pain in parts of the face. 6. Swelling or other changes around the eyes, such as double vision, reduced vision, or a bulging eye. 7. Pain or pressure in the forehead or cheeks. 8. Persistent pain in the upper teeth or pain that feels like a toothache. 9. Loose or numb upper teeth. 10. A lump on the face, nose, or inside the mouth. 11. Unexplained weight loss.   If you have persistent symptoms, it's essential to see a healthcare provider. Although these symptoms can also be due to conditions other than cancer, such as a simple infection, sinusitis, or allergies, it's important to rule out serious conditions, including cancer.  Early diagnosis and treatment of sinonasal cancer are crucial as these cancers can be aggressive and can spread to the brain or other parts of the body if not treated early. Diagnostic tests may include a physical exam, endoscopy, biopsy, and imaging studies such as CT, MRI, or PET scans.  Remember, this information is intended to provide a general understanding of sinonasal cancers. It should not be used as a substitute for medical advice. Always consult with a healthcare professional for accurate information. | Comparable | 1 | 1 | Not applicable |
| 43. What are the symptoms of head and neck cancer of unknown primary site? | Cancers of unknown primary (CUP) are cancers that have spread (metastasized) to parts of the body, but the place the cancer began (the primary site) is not known. When it comes to head and neck CUP, symptoms can vary widely because they depend on the specific location where the cancer has metastasized.  Common symptoms may include:   1. Swelling or a lump in the neck: Often, the first sign of head and neck cancer is a lump in the neck. Cancer that starts in the lymph nodes themselves will often present this way. 2. Chronic sore throat: An ongoing sore throat that doesn't get better could be a sign of cancer. 3. Voice changes: Hoarseness or other changes in the voice can occur. 4. Pain: This might be in the neck, throat, or ears. 5. Weight loss: Unexplained weight loss is a common symptom of many types of cancer, including head and neck cancer. 6. Persistent bad breath or foul taste in the mouth: This might be due to the cancer affecting the mouth or throat. 7. Difficulty swallowing: If cancer has affected the throat, swallowing might become difficult. 8. Oral lesions or white patches in the mouth or throat. 9. Earaches or hearing loss: If the cancer is near the ear, it may cause these symptoms. 10. Nasal congestion or persistent sinus infections that do not respond to treatment.   Keep in mind that these symptoms can also be caused by conditions that are not cancer, such as an infection or an injury. If you or someone else has these symptoms, it's important to see a healthcare professional to get the appropriate diagnosis and treatment.  CUPs can be difficult to diagnose because the primary site is not known, so they often require extensive testing and specialized diagnostic methods. | Comparable | 1 | 1 | Not applicable |
| 44. How is head and neck cancer of unknown primary site diagnosed? | Head and neck cancers of unknown primary site are quite challenging to diagnose because the original site or origin of the cancer is not immediately clear. This type of cancer is identified when cancer cells are found in the lymph nodes of the neck, but the primary source of the cancer, typically located elsewhere in the body, is not identifiable using standard diagnostic procedures.  Here are the common steps used to diagnose this condition:   1. Physical Examination: A doctor will check for any lumps or abnormalities in the head and neck region. 2. Imaging Tests: Imaging modalities like CT (Computed Tomography) scans, MRI (Magnetic Resonance Imaging), PET (Positron Emission Tomography) scans or ultrasound may be used to search for the primary tumor. 3. Endoscopy: This involves using a special instrument to visually inspect areas that might be the primary site. This could include a nasopharyngoscopy (for the nasal cavity and throat), laryngoscopy (for the voice box), or bronchoscopy (for the lungs). 4. Biopsy: This involves taking a sample of tissue from the affected lymph nodes and examining it under a microscope to determine the type of cancer cells present. 5. HPV Testing: Many oropharyngeal cancers are associated with human papillomavirus (HPV) infection. Therefore, a test for HPV might be done on the biopsy sample. 6. EBV Testing: Epstein-Barr virus is associated with nasopharyngeal cancer. A test for EBV might be done if nasopharyngeal cancer is suspected. 7. Laboratory Tests: Blood tests and other lab tests may be performed to check for signs of cancer in the body.   Even after all these investigations, in some cases, the primary site of the cancer may remain unknown. The management of such cases is quite complex and requires a multidisciplinary team including medical oncologists, radiation oncologists, surgeons, pathologists, and radiologists. Treatment may involve radiation therapy, chemotherapy, targeted therapy, surgery, or a combination of these based on the patient's general health status, the type | Comparable | 1 | 1 | Not applicable |
| 45. What kind of ear pain might a person with throat cancer experience? | Ear pain associated with throat cancer is typically a referred pain, which means that it's experienced in a location different from where the actual problem is occurring. This is because the nerves in the throat and ear are closely interconnected.  Individuals with throat cancer may experience a persistent earache that does not resolve with typical treatments, such as antibiotics. The pain may be constant or intermittent, and it could range from a dull ache to a sharp, stabbing pain.  However, ear pain is not a definitive symptom of throat cancer. It can be caused by many other conditions like middle ear infections, sinus infections, dental problems, or temporomandibular joint disorders (TMJ).  If someone is experiencing persistent ear pain, especially in conjunction with other symptoms such as persistent sore throat, difficulty swallowing, changes in voice, unexplained weight loss, or the presence of a lump in the throat or neck, they should seek medical advice as soon as possible. This combination of symptoms could indicate a serious condition such as throat cancer, but only a healthcare professional can provide an accurate diagnosis.  As always, early detection and treatment are key for the best outcomes with cancer and other serious illnesses. So, if you have any concerns about these symptoms, please seek medical attention promptly. | Comparable | 1 | 1 | Not applicable |
| 46. What does it indicate if an MRI scan shows inflamed lymph node tissue in the neck? | Inflamed lymph nodes in your neck seen on an MRI scan can be a sign of various health conditions. Lymph nodes are small, bean-shaped structures that produce and store cells that fight infection and disease. They are part of the body's lymphatic system.  When lymph nodes become inflamed or enlarged, it's usually because they're fighting off an infection or disease. Inflammation or enlargement can be localized to one area or be widespread throughout the body.  Here are several potential causes of inflamed lymph nodes in the neck:   1. Infections: This is the most common cause. Infections such as strep throat, ear infections, dental infections, or skin infections can all cause lymph nodes in the neck to become inflamed. 2. Immune Response: Conditions like lupus or rheumatoid arthritis, where the immune system is overactive, can cause inflammation in lymph nodes. 3. Cancers: Certain types of cancer, such as lymphoma or leukemia, can cause lymph nodes to become inflamed. Additionally, cancers that spread (metastasize) from other parts of the body, like lung or breast cancer, may also involve the lymph nodes. 4. Other Medical Conditions: Less common conditions like sarcoidosis or Castleman disease can affect the lymph nodes. 5. Medications: Certain drugs, like antiseizure medications or preventive treatments for malaria, can cause lymph nodes to swell.   Remember, this is not an exhaustive list, and having inflamed lymph nodes doesn't necessarily mean you have a serious illness. However, if you're concerned about the results of your MRI, you should follow up with your healthcare provider to discuss the potential causes and appropriate next steps for diagnosis or treatment. They might recommend further tests, such as blood work, a biopsy of the lymph node, or additional imaging studies. | Comparable | 1 | 1 | Not applicable |
| 47. What type of tests should be conducted to determine the extent of metastasis in buccal mucosa carcinoma? | Carcinoma of the buccal mucosa is a type of oral cancer. If there's a suspicion of metastasis (spread of cancer from the primary site to other areas of the body), several diagnostic tests can be done to confirm this and determine the extent of the spread. They include:   1. Imaging Tests: Imaging studies can show whether cancer has spread to other parts of the body. This can include:    - Computed Tomography (CT) scan: A CT scan can provide detailed images of any part of the body, including the bones, muscles, fat, and organs.    - Magnetic Resonance Imaging (MRI): MRI uses a powerful magnetic field and radio waves to produce detailed images of the inside of the body.    - Positron Emission Tomography (PET) scan: PET scans can be useful in determining whether cancer has spread to the lymph nodes or other parts of the body.    - Chest X-ray: A simple chest X-ray can identify if the cancer has metastasized to the lungs.    - Bone Scan: If there are symptoms suggestive of bone involvement, a bone scan can be ordered to evaluate for metastasis to the bones. 2. Biopsies: A biopsy involves taking a small sample of tissue from a suspected metastatic site for examination under a microscope. The pathologist can often determine not only if cancer is present but also the type and grade of the cancer. 3. Lab tests: Certain blood tests can provide information about a person's overall health, the functioning of their organs, and whether cancer has spread. For example, liver function tests can suggest whether the cancer has spread to the liver.   Remember, the selection of tests depends on the individual's situation, including their overall health, the type and stage of cancer, and their symptoms. The doctor will use the results of these tests to help decide on the best course of treatment. | Comparable | 1 | 1 | Not applicable |
| 48. What is the purpose of a sentinel node biopsy in the case of head and neck cancer? | Sentinel lymph node biopsy (SLNB) is a surgical procedure used to determine whether certain types of cancer, such as head and neck cancer, have spread (metastasized) to the lymph nodes. The sentinel nodes are the first few lymph nodes into which a tumor drains and the ones that cancer cells are most likely to reach first. The sentinel node is therefore the first place that cancer is likely to spread.  The concept behind SLNB is that if the sentinel lymph node does not contain cancer, then it is highly likely that the cancer has not spread to any other area of the body. If the sentinel lymph node is found to contain cancer, then additional lymph nodes may need to be removed and examined.  In the context of head and neck cancer, particularly with oral and oropharyngeal squamous cell carcinomas, sentinel lymph node biopsy can be used as a minimally invasive procedure to accurately stage the regional lymph nodes. It provides valuable information and helps to guide the appropriate treatment strategy.  For instance, if a sentinel node biopsy shows that cancer has not spread to the lymph nodes, a patient might be able to avoid further invasive surgery or treatments like radiation to the neck. If the biopsy shows that cancer has spread to the lymph nodes, then additional treatment such as a neck dissection (surgery to remove lymph nodes in the neck) and/or radiation may be needed.  In essence, the role of SLNB in head and neck cancer is to accurately stage the disease and help make treatment decisions that are best for the patient. It provides a good balance between reducing the morbidity of unnecessary treatment and ensuring optimal oncologic control. | A sentinel node biopsy is a surgical procedure used to determine whether cancer has spread (metastasized) beyond a primary tumor into your lymphatic system. In head and neck cancer, the sentinel lymph node is the hypothetical first lymph node or group of nodes reached by metastasizing cancer cells from a tumor.  Here are some of the uses of sentinel node biopsy in head and neck cancer:   1. Staging: One of the primary uses of sentinel node biopsy is to stage cancer. Staging is a way of describing the size of a cancer and how far it has spread. This can help guide treatment decisions. 2. Reducing Morbidity: Traditional neck dissections to remove lymph nodes can result in significant morbidity, including shoulder dysfunction, nerve injury, and cosmetic defects. Sentinel node biopsy allows for the removal of fewer lymph nodes, potentially reducing these risks. 3. Predicting Prognosis: The presence or absence of cancer cells in the sentinel node can provide important prognostic information. If the sentinel node is free of cancer, it's a good sign that the cancer has not spread. 4. Guiding Further Treatment: If the sentinel node contains cancer, additional lymph nodes in the neck may be removed in a more extensive procedure, or additional treatments like radiation therapy or chemotherapy may be considered.   It's important to note that sentinel node biopsy is not suitable for all types of head and neck cancers. For example, it's not typically used in cancers that have already clearly spread to the lymph nodes, or in certain situations where the primary tumor is very large. The suitability of sentinel node biopsy will depend on individual patient factors, including the type, size, and location of the cancer, as well as the patient's overall health. | 2,2 | 2,1 | 2, 2 |
| 49. How sensitive is a sentinel node biopsy for detecting malignant melanoma with a 2-mm thickness on the cheek? | As of my last update in September 2021, I don't have the exact sensitivity percentage of a sentinel lymph node biopsy for a 2-mm thick malignant melanoma on the cheek. Sensitivity of this test varies based on several factors such as the location of the tumor, the thickness of the tumor, the individual's immune status, the method of biopsy, and the pathologist's experience, among other things. However, it's generally accepted that the thicker the melanoma, the higher the likelihood of detecting metastasis in the sentinel lymph node.  It's important to note that sentinel lymph node biopsy is typically considered for melanomas that are greater than 1mm in thickness, or for those between 0.75 and 1mm with other high-risk features.  I highly recommend reaching out to medical professionals or reading the latest research for the most accurate and up-to-date information on this topic. As with any medical issue, it's crucial to take individual patient factors into account when deciding on the most appropriate course of action. | As of my knowledge cut-off in September 2021, it was established that sentinel lymph node biopsy (SLNB) is a commonly used procedure to evaluate the extent of spread in malignant melanoma. Its sensitivity can depend on various factors including the specific patient, surgeon, and the specifics of the melanoma itself such as location and depth.  However, you asked about the sensitivity of SLNB in a specific scenario: malignant melanoma with 2mm thickness on the cheek. The sensitivity of SLNB varies widely in the literature, with reported values ranging from 80% to 96% for melanomas of varying depths.  It's important to note that the sensitivity can depend greatly on the thickness of the melanoma - with thinner melanomas having lower sensitivity (since there's less chance for the cancer to have spread to the lymph nodes), and thicker melanomas having higher sensitivity. For a melanoma of 2mm thickness, the sensitivity might be somewhere in the middle of the range.  For the most current and specific statistics, I recommend consulting the latest research or a medical professional who can provide the most up-to-date information and interpret it in the context of a specific patient's situation. Please note that this information is for educational purposes and any medical concerns should be addressed by a healthcare provider. | 2,1 | 1,1 | 2,1 |
| 50. Can head and neck cancer affect breathing? | Yes, head and neck cancers can potentially affect breathing, depending on the location and size of the tumor.   1. Laryngeal cancer: This affects the larynx or voice box, which is a crucial part of the airway. A large tumor in this area can obstruct the airflow, making it difficult to breathe. 2. Nasopharyngeal cancer: Cancer in the nasopharynx, the area at the upper part of the throat behind the nose, can also interfere with breathing, particularly if the tumor grows large enough to obstruct the nasal passages. 3. Oropharyngeal and oral cancer: These cancers affect the mouth and the middle part of the throat. They are less likely to cause breathing difficulties unless they grow very large or spread to other parts of the throat. 4. Hypopharyngeal cancer: This type of cancer affects the lower part of the throat, which can interfere with the airway and cause breathing problems. 5. Tracheal cancer: Although it's not typically classified as a head and neck cancer, it's worth noting that cancer of the trachea (or windpipe) can directly interfere with breathing.   It's important to mention that not all breathing problems are caused by cancer. Other conditions like asthma, COPD, infections, or even anxiety can cause similar symptoms. If you or someone else is experiencing difficulty breathing, it's important to seek medical attention right away. | Yes, head and neck cancer can affect breathing in a number of ways, depending on the specific type and location of the cancer. Here's how:   1. Obstruction: Tumors in the throat, larynx (voice box), or trachea (windpipe) can grow to a size that obstructs the airway, making breathing difficult. 2. Post-surgical changes: If surgical treatment is required, depending on the extent and location of the surgery, it can lead to changes in the airway that may impact breathing. In extreme cases, such as total laryngectomy (removal of the voice box), a permanent hole (stoma) may be created in the front of the neck for breathing. 3. Swelling or inflammation: Both cancer itself and treatments like radiation therapy can cause swelling or inflammation in tissues of the throat or neck, which can interfere with normal airflow. 4. Impaired swallowing: Head and neck cancers often interfere with the normal swallowing process. If food or drink enters the windpipe instead of the esophagus (a condition known as aspiration), it can lead to coughing, choking, and serious lung infections like pneumonia. 5. Lung metastasis: Though not as common, if the cancer spreads (metastasizes) to the lungs, it can cause breathing difficulties. 6. Lymph node involvement: If the cancer spreads to lymph nodes in the neck, they may become enlarged and cause discomfort or difficulty breathing.   It's important to note that these are potential scenarios, and not every person with head and neck cancer will experience these issues. Treatment plans are designed to control the cancer and maintain quality of life, including normal breathing, as much as possible. Individuals should always consult their healthcare providers for information tailored to their specific situation. | 1,2 | 1,1 | 1,2 |
| 51. What causes hearing loss in nasopharyngeal carcinoma? | Nasopharyngeal carcinoma is a type of cancer that occurs in the nasopharynx, the upper part of the throat behind the nose. Hearing loss in nasopharyngeal carcinoma can occur due to several reasons:   1. Tumor Growth: The nasopharynx is located near the Eustachian tubes, which help to equalize pressure in the middle ear. If a tumor grows large enough, it can block these tubes, leading to conditions such as serous otitis media, where fluid accumulates in the middle ear, leading to conductive hearing loss. 2. Radiation Therapy: Treatment for nasopharyngeal carcinoma often involves radiation therapy. Radiation can damage the structures of the ear, particularly the delicate inner ear structures responsible for hearing, such as the cochlea. This can cause sensorineural hearing loss. 3. Chemotherapy: Some types of chemotherapy drugs can be ototoxic, which means they can damage the ear, leading to hearing loss. This type of hearing loss can be temporary or permanent. 4. Nerve Damage: In rare cases, the tumor itself or surgical intervention may cause damage to the auditory nerve, leading to hearing loss.   It's also worth noting that the symptoms of nasopharyngeal carcinoma can vary greatly from person to person, and not all patients will experience hearing loss. Any suspected symptoms should be thoroughly evaluated by a healthcare provider. | Comparable | 1 | 1 | Not applicable |
| 52. What does it mean when a tongue cancer has a depth of invasion greater than 5 mm? | Depth of invasion (DOI) is a measure used in the staging of oral cancers, including tongue cancer, which quantifies how deeply the cancer has invaded into the tissues. It's typically measured from the basement membrane of the mucosa (the superficial layer) to the deepest point of tumor invasion.  When you hear "depth of invasion more than 5 mm," it means the cancer has infiltrated more than 5 millimeters from the surface of the tissue into the underlying structures.  This depth of invasion is an important factor in staging and prognosis of oral cavity cancers. Research has shown that the deeper the tumor invasion, the higher the risk of metastasis (spread of the cancer to other parts of the body), and consequently, a poorer prognosis.  Moreover, the depth of invasion is also important when considering the surgical approach and the need for additional therapies such as radiation or chemotherapy. In general, cancers with a greater DOI often require more aggressive treatment.  Remember, specific treatment decisions should always be made in consultation with a healthcare provider who has access to all pertinent patient information. It's essential to remember that individual patient characteristics can significantly impact the course of the disease and its treatment. | Comparable | 1 | 1 | Not applicable |
| 53. What does extracapsular spread of lymph nodes in the neck signify? | Extracapsular spread (ECS) of lymph nodes in the neck refers to the situation where cancerous cells have broken through the lymph node's outer membrane (or capsule) and started to infiltrate surrounding tissues. This typically happens in the context of head and neck cancers, such as squamous cell carcinoma, but can occur in other cancers that spread to neck lymph nodes as well.  The term "extracapsular" means "outside the capsule", denoting that the cancer cells are no longer contained within the lymph node.  ECS is an important prognostic factor in cancer. It is associated with a higher risk of recurrence and poorer survival rates compared to cases where cancer is confined to the lymph nodes. This is due to the increased likelihood of microscopic spread of disease, making it more challenging to completely remove or treat the cancer.  The presence of ECS can influence treatment decisions. For instance, it may prompt more aggressive treatments such as higher doses of radiation or the use of chemotherapy in addition to surgery or radiation.  If a patient has a diagnosis of ECS, their healthcare provider will likely discuss this in the context of their overall prognosis and treatment plan. It is always a good idea to ask questions and ensure you understand all aspects of your healthcare. | Comparable | 1 | 1 | Not applicable |
| 54. I have been diagnosed with a thyroglossal duct cyst. Is there a risk of cancer associated with this diagnosis? | A thyroglossal duct cyst is a generally benign (noncancerous) condition that occurs due to a leftover piece of tissue from embryonic development. They are the most common type of congenital neck abnormality and usually present as a midline neck lump, often moving up with swallowing or sticking out the tongue.  However, there is a small risk that a thyroglossal duct cyst can develop into a specific type of cancer known as thyroglossal duct cyst carcinoma. The reported incidence of carcinoma arising from a thyroglossal duct cyst is low, approximately 0.7% to 1.0%. Most of these cases are papillary thyroid carcinomas, a type of thyroid cancer that usually has a good prognosis.  Keep in mind that this is a rare occurrence and most thyroglossal duct cysts do not develop into cancer. It's important to follow your healthcare provider's advice regarding monitoring or treatment of a thyroglossal duct cyst. Surgical removal, known as Sistrunk procedure, is usually performed to prevent complications like infection or to rule out the presence of cancer. Always speak to your doctor or a specialist about your concerns, as they can give you personalized advice based on your health history and current condition. | Comparable | 1 | 1 | Not applicable |
| 55. What does perineural invasion mean in my oral cancer surgery pathology report? | Perineural invasion (PNI) is a term used to describe the situation where cancer cells are found along the nerves within the tissue that was removed during surgery. In the context of oral cancer, it's a pathologic feature that can potentially impact prognosis and treatment decisions.  It's called 'perineural' because it refers to the area around ('peri-') the nerves. 'Invasion' implies that the cancer cells have spread into these areas. Essentially, the cancer cells are 'invading' the nerves within the tissue. This can happen in many different types of cancer, not just oral cancer.  However, it's important to note that the presence of PNI does not necessarily change the overall stage of the cancer, but it might suggest a higher risk of local recurrence and possibly a worse prognosis. Some studies also suggest that PNI may be associated with an increased likelihood of metastasis, particularly in advanced stages of oral cancer.  Treatment for oral cancers with PNI typically involves surgery to remove the tumor, often followed by radiation therapy and sometimes chemotherapy to kill any remaining cancer cells and reduce the risk of the cancer coming back.  However, every case is unique. Please discuss these findings with your healthcare provider for more specific information pertaining to your condition. The implications of PNI can vary based on the overall context of the disease including the type of oral cancer, the stage, and other pathologic findings in the report. | Comparable | 1 | 1 | Not applicable |
| 56. What does lymphovascular invasion in my throat cancer surgery pathology report mean? | Lymphovascular invasion (LVI) is a term that refers to the presence of cancer cells in the blood vessels or lymphatic system. It is an important piece of information in a pathology report because it can influence the staging of the cancer, potential treatment decisions, and overall prognosis.  In the context of throat cancer, lymphovascular invasion suggests that the cancer has spread, or has the potential to spread, beyond the original site to other parts of the body through the bloodstream or lymphatic system. This might suggest a more aggressive form of the disease and could lead to different or additional treatment approaches, such as radiation therapy, chemotherapy, or targeted therapies.  However, the presence of LVI doesn't necessarily mean that cancer has already spread. It is simply a potential risk factor for future spread (metastasis). Therefore, doctors will usually recommend further tests to check for metastases.  Keep in mind that this information should be discussed with your healthcare provider to fully understand its implications in your specific case. Treatment options, risks, and prognosis vary depending on the type and stage of the cancer, your overall health, and many other factors. | Comparable | 1 | 1 | Not applicable |
| 57. I have a 3 mm Breslow depth malignant melanoma on my lip. What is the likelihood of sentinel lymph node positivity? | Sentinel lymph node (SLN) positivity rate can vary greatly depending on the specific case, but in general, it tends to increase as Breslow thickness increases.  As of my last update, studies showed that the rate of sentinel lymph node positivity in melanomas with Breslow thickness of 2.01-4.00 mm ranged from approximately 20% to 30%. The actual rate can vary depending on many factors, such as the age and overall health of the patient, the exact location of the melanoma, and other characteristics of the tumor itself.  However, since your melanoma is located on your lip, it falls into the category of mucosal melanomas, which are relatively rare and can have different behavior than cutaneous melanomas. This could potentially affect SLN positivity rates, but specific rates for mucosal melanomas weren't widely reported as of 2021.  Keep in mind that data and treatment approaches may have changed since my last update. It's crucial to discuss your case with your healthcare provider or oncologist for the most accurate and up-to-date information. | Comparable | 1 | 1 | Not applicable |
| 58. How is larynx cancer graded? | Laryngeal cancer, like many cancers, is typically graded and staged based on the severity of the disease and its spread. It is important to note that staging and grading systems may evolve over time as our understanding of the disease grows.  Grading generally refers to how the cancer cells look under a microscope and how much they resemble normal cells. The grades range from well-differentiated (grade 1), which means they look similar to normal cells, to poorly differentiated (grade 3), which means they look very different from normal cells. High grade or poorly differentiated cancers tend to grow and spread more rapidly.  Grading for laryngeal cancer may include:   - Grade 1 (G1): Well-differentiated cancer cells that look very much like normal larynx cells. - Grade 2 (G2): Moderately-differentiated cells that look somewhat like normal cells. - Grade 3 (G3): Poorly differentiated or undifferentiated cells that look very abnormal and are likely to grow and spread more aggressively.   Staging, on the other hand, refers to the extent of the cancer and is based on several factors including the size of the primary tumor, its location, and whether it has spread to nearby lymph nodes or distant sites. The American Joint Committee on Cancer (AJCC) TNM system is commonly used for this, which stands for Tumor size, Node involvement, and Metastasis:   - T stages (T1-T4): Indicate the size and extent of the primary tumor. T1 is small and localized, while T4 is large or has grown into surrounding tissues. - N stages (N0-N3): Indicate whether the cancer has spread to nearby (regional) lymph nodes. N0 means it hasn't spread to any lymph nodes, while N3 means it has spread to multiple or larger lymph nodes. - M stages (M0-M1): Indicate whether the cancer has metastasized (spread) to distant sites in the body. M0 means there is no distant spread, while M1 means that there is.   The combination of these stages is used to derive an overall stage (stage 0 to stage IV), which helps determine the prognosis and treatment plan.  Please note that all of these systems are subject to interpretation by the medical team involved and may not perfectly predict an individual's prognosis or response to treatment. Always discuss your personal situation with your doctor or medical team. They are best equipped to interpret and apply this information to your individual case. | Comparable | 1 | 1 | Not applicable |
| 59. Is sentinel node biopsy applicable in T3N1M0 tongue carcinoma? | In the management of oral cancer, including tongue cancer, the status of the cervical lymph nodes is a crucial factor that significantly influences both prognosis and treatment planning. Generally, the presence of neck node metastasis can reduce survival by 50%. Sentinel lymph node biopsy (SLNB) is a procedure that has been increasingly used for early-stage oral cancers (T1-T2) to avoid a more extensive and potentially unnecessary neck dissection in patients who have no clinical or radiographic evidence of neck disease.  However, in your specific question about a T3N1M0 tongue carcinoma, the cancer is already clinically advanced with confirmed lymph node involvement (N1 denotes metastasis in a single ipsilateral lymph node, not more than 3 cm in greatest dimension). Because of this, sentinel lymph node biopsy is typically not used, since there is already evidence of nodal disease.  In such cases, a more appropriate surgical intervention would be a neck dissection (either selective or modified radical, depending on the clinical scenario and institutional practices) to address the involved lymph node and any potential microscopic disease in other nodes.  Keep in mind that the treatment plan will also depend on other factors such as the patient's overall health status, comorbidities, and personal wishes. It's important to discuss all options and their risks and benefits with the treating physicians. | Comparable | 1 | 2 | 1 |
| 60. My lip resection specimen for lip cancer report indicates 0.5 cm surgical margins. Is this adequate for tumor control? | The adequacy of the surgical margin for tumor control in lip cancer, as with many other cancers, depends on several factors, including the type and stage of the cancer, the grade of the tumor, and other patient-specific factors.  The National Comprehensive Cancer Network (NCCN) Guidelines for Head and Neck Cancers, as of my last update in 2021, recommend a minimum margin of 5mm (or 0.5 cm) for oral cavity squamous cell carcinomas, which include lip cancers, if the tumor is well defined and the surgeon has a high degree of confidence in the completeness of the resection. The guidelines also note that a wider margin may be appropriate for advanced cancers, poorly defined tumors, or tumors with perineural invasion.  While a 0.5 cm margin is often considered adequate, every patient's situation is unique, and this is something that should be discussed with your treating oncologist. They can give you the most accurate assessment based on the specifics of your case. It's important to remember that adequate surgical margins are only one component of comprehensive cancer care, which may also include radiation therapy, chemotherapy, immunotherapy, or targeted therapy, depending on the specific characteristics of the cancer.  Please note that information might have been updated or changed after my last training data in September 2021, and it is always best to consult with your healthcare provider for the most recent and relevant advice. | Comparable | 1 | 1 | Not applicable |
| 61. What is the rate of occult neck metastasis in T3 maxillary sinus cancer? | As of my last update in September 2021, I don't have the specific data for the occult neck metastasis rate for T3 maxillary sinus cancer. The occult metastasis rate in head and neck cancers can vary depending on the site, tumor stage, histological type, and other factors.  In general, higher T stages (T3, T4) are associated with an increased risk of metastasis, but the rates can still be quite variable. For paranasal sinus cancers overall, occult neck metastasis has been reported in a range of approximately 10-20% of cases, though these rates can change based on the factors mentioned above.  Please consult with medical professionals or refer to the latest research or guidelines from reputable oncology associations for the most accurate and current data. It's also important to remember that statistics can provide a general idea but they may not perfectly apply to an individual case, as each patient is unique. | Comparable | 2 | 1 | 1 |
| 62. What is PET-CT? | PET-CT stands for Positron Emission Tomography - Computed Tomography. It's a type of nuclear imaging technology that can be used in medicine to visualize and measure various bodily functions.  PET (Positron Emission Tomography) and CT (Computed Tomography) are both standard imaging tools that physicians use to pinpoint disease states in the body. A PET scan demonstrates the biological function of the body before anatomical changes take place, while the CT scan provides information about the body’s anatomy such as size, shape and location.  By combining these two scanning technologies, a PET/CT scan enables physicians to more accurately diagnose and identify cancer, heart disease and brain disorders.  Here's a bit more detail about how each technology works:   1. PET works by using a scanning device (a machine with a large hole at its center) to detect photons that are emitted by a small amount of radioactive substance, or tracer, which is injected into the body. The tracer travels through the body, collecting in areas with high levels of chemical activity (which often correspond to areas of disease), and emits positrons. When these positrons interact with electrons in the body, they produce photons that the scanner can detect. By analyzing this photon data, a computer can produce 3D images of the body that represent the concentration of the tracer, and thus, the body's biochemical activity. 2. CT works by using a different kind of scanning device, which rotates around the body and sends out multiple beams of x-rays. By measuring the x-ray beams' angles and intensities after they pass through the body, a computer can construct a detailed 3D image of the body's structure.   When these two types of scans are performed at the same time (with the same machine), the resulting images provide detailed information about both the structure and function of tissues and organs in the body. This information can be used to diagnose and monitor a wide range of health conditions. | Comparable | 1 | 1 | Not applicable |
| 63. What is the sensitivity and specifity of a fine needle aspiration biopsy for parotid gland tumors? | As of my knowledge cutoff in September 2021, fine needle aspiration biopsy (FNAB) is a common and minimally invasive procedure used to evaluate parotid gland tumors. However, the sensitivity and specificity can vary based on several factors, including the person conducting the procedure, the pathologist interpreting the results, and the nature of the tumor itself.  Broadly, literature shows the sensitivity of FNAB for diagnosing parotid gland tumors varies from 65% to 98%, while specificity ranges from 72% to 100%. For instance, a systematic review and meta-analysis published in the journal "Otolaryngology–Head and Neck Surgery" in 2012 reported a pooled sensitivity of 87.1% and a specificity of 95.6%.  Nevertheless, these figures can vary across different studies and clinical contexts. FNAB is generally more accurate at ruling out malignancy when the result is benign, due to its high specificity. However, false negatives (i.e., failing to detect a malignant tumor) can occur, meaning that a benign FNAB result does not definitively rule out cancer.  It's also important to remember that FNAB is just one tool in the diagnostic arsenal. Clinical history, imaging studies, and the patient's overall health status also play a significant role in forming a comprehensive diagnostic picture.  Since the science of medicine continually evolves, there may be more recent data available beyond my knowledge cutoff. Therefore, for the most updated and accurate information, I recommend referring to recent medical literature or consulting with a healthcare professional. | Comparable | 1 | 1 | Not applicable |
| ***TREATMENT*** |  |  |  |  |  |
| 64.How is T3N1M0 hypopharyngeal cancer typically treated? | T3N1M0 refers to a specific stage of cancer according to the TNM staging system. In this case, the patient has a hypopharyngeal cancer that is:   - T3: The tumor is large and may have grown into tissues around the hypopharynx. - N1: Cancer has spread to one lymph node on the same side of the neck as the tumor, and the lymph node is no larger than 3 cm across. - M0: The disease has not spread to other parts of the body.   Treatment options for T3N1M0 hypopharyngeal cancer typically involve a combination of surgery, radiation therapy, and chemotherapy. The exact treatment plan will depend on the patient's overall health, the exact location and size of the tumor, and other factors.  Here is a general outline of possible treatments:   1. Surgery: A partial or total pharyngectomy (removal of part or all of the pharynx) might be performed. In some cases, a laryngectomy (removal of the voice box) may also be needed. Neck dissection to remove the lymph nodes might also be performed. 2. Radiation therapy: This might be used before surgery (neoadjuvant therapy) to shrink the tumor or after surgery (adjuvant therapy) to kill any remaining cancer cells. Radiation therapy can also be used as the primary treatment if surgery is not an option. 3. Chemotherapy: Chemotherapy drugs might be given concurrently with radiation therapy (chemoradiation) to enhance the effect of radiation. This can also be used before surgery to shrink the tumor or after to kill any remaining cells. 4. Targeted therapy: Drugs that specifically target cancer cells might also be used in combination with other treatments. As of my knowledge cutoff in September 2021, cetuximab (Erbitux) was the only targeted therapy approved for head and neck cancers, but more may have become available since then. 5. Immunotherapy: This uses the patient's immune system to fight the cancer. As of 2021, drugs like pembrolizumab (Keytruda) and nivolumab (Opdivo) were used in certain situations for advanced or recurrent head and neck cancers. 6. Rehabilitation: Because treatment for hypopharyngeal cancer can affect speech and swallowing, speech and swallowing therapists are an important part of the treatment team. 7. Follow-up care: This is necessary to monitor for cancer recurrence or spread, as well as to manage ongoing health issues due to the cancer and its treatment.   This is a general overview of treatment options, but the exact approach depends on many factors. It's important to discuss all treatment options, including goals and possible side effects, with the healthcare team to make the best choice. Each patient is unique and individual circumstances will dictate the optimal treatment plan. | Comparable | 1 | 1 | Not applicable |
| 65. I have a metastatic jaw tumor that has spread to my lungs and bones. What are the appropriate treatment options? | Treatment of metastatic cancer involves multiple modalities depending on the type of primary cancer, the extent of spread, your overall health status, and your personal preferences. Broadly, these can include:   1. **Surgery**: While surgery might be used to remove the primary tumor or metastases in certain situations, it is often not the primary treatment for metastatic cancer due to the widespread nature of the disease. 2. **Radiation Therapy**: This uses high-energy particles or waves, such as X-rays, gamma rays, electron beams, or protons, to destroy or damage cancer cells. Radiation might be used to treat both the primary site and areas of metastasis. 3. **Chemotherapy**: These are drugs that kill cancer cells throughout the body, making them useful for treating metastatic disease. 4. **Targeted Therapy**: These are drugs designed to target specific genes or proteins that allow cancer cells to grow and survive. 5. **Immunotherapy**: These treatments help your immune system fight cancer. Some types of immunotherapy help train your immune system to attack cancer cells specifically, while others enhance your immune system's natural ability to fight off cancer. 6. **Hormone Therapy**: If the cancer is hormone-sensitive, drugs to block the body's hormones might be used. 7. **Palliative Care**: This focuses on relieving symptoms and improving the quality of life. It can be used at any stage of illness, not just advanced cancers, and it can be used along with curative treatments. 8. **Clinical Trials**: In some cases, patients might opt to participate in clinical trials investigating new treatment approaches.   However, the appropriate treatment option depends on several factors including the type of cancer, the extent of spread, your overall health, your personal preference, and more. It's crucial to have a detailed conversation with your healthcare provider to understand what treatment is most suitable for you.  This information is subject to change and advancements in the field of oncology, so new therapies or strategies may have emerged since my last update in 2021. Please rely on your healthcare providers for the most accurate and up-to-date information. | Comparable | 2 | 2 | Not applicable |
| 66. How is T4N0M0 laryngeal cancer with extralaryngeal spread treated? | Laryngeal cancer treatment depends on the stage of the cancer, the person's overall health, and personal preferences. T4N0M0 laryngeal cancer means the cancer is very large or has grown into nearby structures (T4), there is no evidence of spread to nearby lymph nodes (N0), and there's no distant metastasis (M0).  The primary treatment options for T4 laryngeal cancer, especially with extralaryngeal spread, include surgery, radiation therapy, and chemotherapy, which can be used individually or combined, depending on the specific circumstances.   1. Surgery: Depending on the extent and location of the cancer, surgery may involve partial or total laryngectomy, where part or all of the larynx (voice box) is removed. Lymph nodes in the neck might also be removed (neck dissection) if they're at risk of containing cancer cells. For T4 laryngeal cancer, particularly with extralaryngeal spread, total laryngectomy is often performed. 2. Radiation therapy: This treatment uses high-energy beams, such as X-rays and protons, to kill cancer cells. It can be used before surgery to shrink a tumor, after surgery to kill any remaining cells, or as a primary treatment in cases where surgery isn't an option. 3. Chemotherapy: This involves using drugs to kill cancer cells. Chemotherapy may be given before surgery to shrink tumors (neoadjuvant chemotherapy), after surgery to kill remaining cells (adjuvant chemotherapy), or in combination with radiation therapy (chemoradiation) which can increase the effectiveness of the radiation therapy. 4. Chemoradiation: For some cases of advanced laryngeal cancer, concurrent chemotherapy and radiation therapy may be recommended. This approach can be an alternative to surgery and can sometimes preserve the larynx. 5. Targeted therapy and Immunotherapy: These newer types of cancer treatment may also be an option, especially in cases where other treatments are not effective. They include medications that specifically target cancer cells or boost the body's immune system to better fight off cancer. 6. Rehabilitation: After treatment, rehabilitation is often necessary. This may involve speech therapy, swallowing therapy, and physical therapy to address issues resulting from the cancer or its treatment.   The exact treatment plan should be personalized based on the individual's overall health, the exact location and size of the tumor, and their personal preferences. Therefore, it's important that any person with laryngeal cancer have a thorough discussion with their oncology team to understand the best treatment options for their particular situation. | The T4N0M0 classification in the TNM staging system for laryngeal cancer describes a cancer that has advanced locally (T4), but has not spread to nearby lymph nodes (N0) and there's no evidence of distant metastasis (M0). T4 indicates a tumor that has grown into nearby structures outside the larynx (extralaryngeal spread).  The treatment approach to such a condition may vary depending on the patient's overall health, age, personal preference, and the specific structures involved, but it often involves a combination of the following:   1. Surgery: Advanced local spread often necessitates more extensive surgery, such as a total laryngectomy, which involves removing the entire larynx. Some patients may also require additional surgery to remove affected surrounding tissues or structures. A tracheostomy, creating a hole in the front of the neck for breathing, is usually required after laryngectomy. 2. Radiation therapy: This could be used as an alternative to surgery in some cases, or in conjunction with surgery. Radiation can be postoperative, to destroy any cancer cells that might have been left behind, or it can be used as primary treatment in an attempt to preserve laryngeal function. 3. Chemotherapy: Often used in combination with radiation (chemoradiation) to enhance the effects of radiation. In some cases, induction chemotherapy is given before radiation to try to shrink the tumor. 4. Targeted therapy or immunotherapy: These newer treatment modalities have been showing promise in the treatment of head and neck cancers. These treatments are designed to target specific characteristics of cancer cells or to stimulate the body's immune system to fight the cancer. 5. Rehabilitation: After treatment, rehabilitation such as speech therapy is often necessary, especially if the larynx has been removed or if the patient has difficulty swallowing.   Every patient's situation is unique, so it is very important that all treatment decisions be made in consultation with a team of healthcare professionals who specialize in treating laryngeal cancer. Also, ongoing research and clinical trials may provide new, promising options for treatment. | 3,1 | 3,2 | 3,2 |
| 67. How is T2N2M0 tongue cancer treated? | The treatment for T2N2M0 tongue cancer would typically involve a combination of surgery, radiation therapy, and possibly chemotherapy, depending on various factors like the patient's overall health, the exact location of the tumor, the patient's preferences, and more.   1. Surgery: This is the primary treatment for most forms of oral cancer including tongue cancer. The goal is to remove the tumor and some surrounding healthy tissue. A neck dissection may also be performed to remove lymph nodes in the neck. 2. Radiation therapy: This uses high-energy beams, like X-rays and protons, to kill cancer cells. It can be delivered externally or, in certain cases, internally (brachytherapy). Radiation therapy is often used after surgery or in combination with chemotherapy for more advanced cancers. 3. Chemotherapy: This uses drugs to kill cancer cells. Chemotherapy might be used in conjunction with radiation therapy to enhance the cancer-killing effect, a process known as chemoradiation. 4. Targeted drug therapy: This treatment uses drugs that attack specific vulnerabilities in the cancer cells. They might be used in conjunction with chemotherapy or radiation therapy. 5. Immunotherapy: This is a treatment that uses the body's immune system to fight cancer. It's generally reserved for advanced or recurrent cancers that do not respond to other treatments.   Patients should have a detailed discussion with their healthcare team to understand the potential benefits and risks of each treatment option. The healthcare team will be able to offer the best advice tailored to the patient's individual circumstances.  After treatment, follow-up care to monitor for recurrence and manage any side effects or complications is important. Rehabilitation, such as speech and swallowing therapy, may be necessary depending on the extent of the surgery. Emotional support and palliative care to manage symptoms and improve quality of life are also important components of care. | Comparable | 1 | 1 | Not applicable |
| 68. How is metastatic squamous neck cancer with an occult primary treated? | Metastatic squamous neck cancer with an occult primary is a rare clinical condition where squamous cell carcinoma is found in the lymph nodes of the neck, but the place in the body where the cancer started (the primary site) can't be identified despite a thorough evaluation. This scenario accounts for about 2% to 4% of all head and neck cancers.  It's important to remember that specific treatments may vary depending on the individual patient's overall health, the stage and exact location of the cancer, and other factors. Your healthcare provider will discuss the best options for your particular case.  As of my knowledge cutoff in 2021, there are several treatment approaches for metastatic squamous neck cancer with occult primary:   1. Surgery: Surgical removal of the lymph nodes in the neck (neck dissection) is often the first line of treatment. If the primary cancer is found during surgery, it may also be removed if it's accessible and safe to do so. 2. Radiation Therapy: After surgery, radiation therapy is typically used to kill any remaining cancer cells and to treat the potential primary sites in the upper aerodigestive tract. This often includes the nasopharynx, oropharynx, hypopharynx, and larynx, as well as the area where the lymph nodes were removed. 3. Chemotherapy: This can be used in combination with radiation (chemoradiation) for more advanced stages or if the cancer is inoperable. In some cases, chemotherapy might be given before surgery (neoadjuvant chemotherapy) to shrink tumors, or after surgery (adjuvant chemotherapy) to kill any remaining cancer cells. 4. Immunotherapy: New treatments like immunotherapy drugs, which help the patient's own immune system fight the cancer, may also be an option. Some types of immunotherapy, like checkpoint inhibitors, have shown promise in treating head and neck cancers. 5. Targeted Therapy: These are drugs that specifically target cancer cells or the molecular pathways that allow the cancer cells to grow and divide, with the intent of causing less harm to normal cells compared to chemotherapy. 6. Clinical trials: Clinical trials may also be an option, where new and potentially more effective treatments are being studied.   As mentioned earlier, the treatment protocol for each patient will be individualized based on many factors. It's crucial to have detailed discussions with your healthcare provider or oncologist to understand the benefits, risks, and side effects of each treatment.  Please note that medical science is always advancing, and new treatments or changes in treatment strategies may have been developed after my training data was last updated in September 2021. Always consult with a healthcare provider for the most accurate and current information. | Comparable | 1 | 1 | Not applicable |
| 69. What is the first line treatment for T2N0M0 nasopharyngeal cancer? | As an AI with knowledge up to September 2021, the primary first-line treatment for T2N0M0 (stage II) nasopharyngeal cancer usually involves radiation therapy, and sometimes concurrent chemoradiation (chemotherapy and radiation therapy together). Nasopharyngeal cancers are particularly sensitive to both radiation and chemotherapy.  The decision on whether to use radiation alone or in combination with chemotherapy can depend on a number of factors including the patient's overall health, age, and preference. It's also based on factors like the exact location and size of the tumor.  In recent years, intensity-modulated radiation therapy (IMRT), a type of radiation therapy that uses computer-controlled x-rays to deliver precise radiation doses to a malignant tumor or specific areas within the tumor, has become a standard technique for treating nasopharyngeal cancer, as it can help to reduce the side effects associated with radiation treatment.  However, it's important to note that treatment recommendations could change over time as more research is conducted and newer treatments are developed. Always consult a healthcare professional or oncologist for up-to-date, personalized medical advice. | Comparable | 1 | 1 | Not applicable |
| 70. What advantages does endoscopic surgery have over open surgery for frontal sinus cancer? | Endoscopic surgery has several advantages over open surgery for frontal sinus cancer, although the optimal choice will always depend on the specifics of the case, including the type, location, and stage of the cancer. Below are some general advantages of endoscopic surgery:   1. Minimally Invasive: The primary advantage of endoscopic surgery is that it is less invasive. Surgeons use a thin tube called an endoscope, which is equipped with a light and camera to visualize and operate in the sinus cavities through the nose, without making large incisions. 2. Reduced Recovery Time: Because it's minimally invasive, patients typically experience less post-operative discomfort, have a quicker recovery time, and shorter hospital stays. 3. Less Scarring: As the surgery is done through the nasal cavity, it leaves no visible scars, as opposed to open surgery which may require incisions on the face or skull. 4. Reduced Risk of Complications: There are often fewer complications with endoscopic surgery, such as infection and bleeding, because of the smaller incision size. 5. Preservation of Structures: Endoscopic surgery often allows for better preservation of normal anatomy, which can result in better functional and aesthetic outcomes. 6. Better Visualization: The endoscope can provide better illumination and magnification of the surgical area, potentially allowing for a more precise surgery.   While these are general advantages, it's important to understand that each case of frontal sinus cancer is unique. Factors such as the extent of the tumor, whether the cancer has spread, and the patient's overall health will influence the best surgical approach. Furthermore, not all surgeons have equal experience or skill with endoscopic techniques, which is another important consideration. | Comparable | 1 | 1 | Not applicable |
| 71. What is the difference between proton beam radiotherapy and traditional radiotherapy? | Both proton beam radiotherapy and traditional (photon-based) radiotherapy are methods of delivering radiation treatment to patients, typically for the purpose of treating cancer. They differ in the type of radiation used, their precision, cost, and the damage they can potentially cause to healthy tissue surrounding a tumor.  Here are some of the differences:   1. Type of Radiation: Proton beam radiotherapy uses protons, while traditional radiotherapy uses photons. Protons are particles with a positive charge, and photons are particles of light. 2. Precision: Proton beam therapy has a very precise energy deposition pattern, often referred to as the Bragg Peak. This allows most of the proton's energy to be deposited at the tumor site, minimizing the amount of radiation delivered to the surrounding healthy tissues. Photons used in traditional radiotherapy, however, deposit their energy along the entire path they travel, resulting in more exposure to normal tissues both before and beyond the target. 3. Side Effects: Because of the differences in precision, proton beam therapy may result in fewer side effects than traditional radiotherapy. This is because less healthy tissue is exposed to radiation. However, this can vary depending on the specific case and the location and size of the tumor. 4. Cost and Availability: As of my knowledge cutoff in September 2021, proton beam therapy tends to be more expensive than traditional radiotherapy and is less widely available, as it requires specialized and costly equipment. 5. Tumor Type: Both types of therapy can be used for many different types of cancers, but because of its high precision, proton beam therapy is often used for tumors located near sensitive structures where it is crucial to limit radiation exposure. This includes tumors in the brain, spine, and eyes, among others. 6. Evidence and Research: There is extensive research and clinical evidence supporting the use of traditional radiotherapy. Proton beam therapy also has a strong research base, but because it is newer and less common, there may be less long-term data available for certain uses compared to traditional radiotherapy.   In sum, while proton therapy can provide certain advantages, especially in complex cases where the tumor is located near critical structures, the choice between proton and photon therapy will depend on various factors, including the specifics of a patient's cancer, the availability and cost of proton therapy, and the judgement of the treating physicians. | Comparable | 1 | 1 | Not applicable |
| 72. What are the survival rates associated with radiotherapy and surgery for T1N0M0 glottic carcinoma? | As an AI, I don't have real-time data access, and I can only provide information available up to my last update in September 2021. Up to that point, survival rates for early-stage (T1N0M0) glottic carcinoma treated with either radiotherapy or surgery were generally favorable, often exceeding 90%.  A 2017 meta-analysis published in the "Journal of Clinical Oncology" showed that overall survival at 5 years for early-stage glottic cancer treated with radiotherapy ranged from 78% to 94%, while laryngeal preservation rates were 85-100%. It also showed comparable survival rates for endoscopic surgery, with 5-year overall survival rates ranging from 86% to 100%, and laryngeal preservation rates from 94% to 100%.  However, individual outcomes can vary depending on factors like the exact location and size of the tumor, the patient's overall health, the specific treatment protocols used, and the expertise of the treatment team.  For the most up-to-date and personalized information, I would recommend reaching out to a healthcare professional or consulting the latest scientific literature. | Comparable | 1 | 1 | Not applicable |
| 73. What is transoral robotic surgery? | Transoral robotic surgery (TORS) is a type of minimally invasive procedure performed through the mouth using a sophisticated, computer-enhanced system that allows surgeons to visualize the treatment area and manipulate surgical instruments with precise control. The robot doesn't perform surgery, but rather the surgeon uses the robotic system as a tool to enhance their own skills and capabilities.  TORS was developed to remove benign and malignant tumors of the mouth and throat, especially those located in hard-to-reach areas. The use of robotic technology allows for a more precise operation, which can minimize damage to surrounding tissues, reduce post-operative pain, decrease the risk of complications, and often results in a quicker recovery time for the patient.  During the procedure, the surgeon sits at a console from which they can control the robotic system. The system typically includes a 3D camera for a magnified, high-definition view of the surgical site, and arms that can hold and manipulate surgical instruments with a high degree of precision and flexibility.  As of my knowledge cut-off in September 2021, the most commonly used system for TORS is the da Vinci Surgical System. This system has been used in a wide variety of surgical procedures, including urological, gynecological, colorectal, cardiothoracic and general surgical procedures, in addition to its applications in head and neck surgery. | Comparable | 1 | 1 | Not applicable |
| 74. What are the indications for radiotherapy after surgery for tongue cancer? | After surgical removal of tongue cancer, radiotherapy (often as part of a regimen called adjuvant therapy) may be recommended to kill any remaining cancer cells and reduce the risk of recurrence. Indications for adjuvant radiotherapy may include:   1. Positive surgical margins: If cancer cells are found at the edge of the tissue removed during surgery, it suggests that some cancer might have been left behind, and radiotherapy can be used to treat these cells. 2. High-grade tumors: Tumors that are more abnormal (high grade) are more likely to grow and spread quickly. Radiotherapy can be used to kill any potentially aggressive cancer cells remaining after surgery. 3. Advanced-stage disease: If the cancer was at a more advanced stage at the time of surgery (for example, it had spread to nearby lymph nodes or tissues), radiotherapy can be used to treat any residual disease. 4. Extracapsular spread or perineural invasion: If cancer has spread outside the lymph node capsule or along nerves, radiotherapy can help to treat these more extensive areas of disease. 5. Close surgical margins: Even if the surgical margins aren't positive, if they are close it might indicate a higher risk of remaining disease. 6. Multiple lymph nodes involved: If cancer has spread to multiple lymph nodes, it increases the risk of residual disease after surgery. 7. Lymphovascular invasion: This refers to cancer spreading into blood vessels or lymph vessels, which can increase the risk of distant spread. 8. Tumor thickness or depth of invasion: A thicker tumor or deeper invasion into the tongue tissue might indicate a higher risk of remaining disease or spread to other areas, which can be treated with radiotherapy.   These factors can help clinicians decide whether radiotherapy is likely to be beneficial after surgery for tongue cancer. However, every patient is different, and the potential benefits of radiotherapy must be balanced against potential side effects, such as damage to healthy tissues, dry mouth, difficulty swallowing, changes in taste, and mouth sores. Therefore, the decision will be personalized based on the patient's individual circumstances and preferences. | Radiotherapy, sometimes combined with chemotherapy (called chemoradiotherapy), is often used as a follow-up treatment to surgery for tongue cancer. The indications to use postoperative radiotherapy typically depend on the stage and the specific features of the tumor, as well as whether or not there is lymph node involvement. Here are some of the potential indications:   1. High grade tumors: High grade or poorly differentiated tumors have a higher risk of recurrence and might warrant postoperative radiotherapy. 2. Close or positive surgical margins: If cancer cells are found at the edge of the tissue removed during surgery (positive margins), or if the margins are very close, there's a higher risk that some cancer may have been left behind. In these cases, radiotherapy can be used to kill any remaining cells. 3. Extracapsular spread: If the cancer has spread outside of the lymph nodes (extracapsular spread), this is associated with a higher risk of recurrence. 4. Multiple lymph node involvement: If cancer is found in multiple lymph nodes, there's a greater chance that the cancer may come back, warranting additional treatment with radiation therapy. 5. Perineural or vascular invasion: If cancer cells are found in the nerves or blood vessels, this can increase the risk of recurrence and may indicate the need for postoperative radiotherapy. 6. Advanced stage disease (T3, T4): In later stages of tongue cancer, the tumor is larger and may have grown into nearby tissues or organs. In these cases, postoperative radiotherapy may be used to try to kill any remaining cancer cells. 7. Residual disease: If there's still evidence of disease following surgery, then radiotherapy may be used as a supplemental treatment.   It's important to note that while radiotherapy can help improve survival rates and decrease the chances of recurrence, it can also have side effects like dry mouth, difficulty swallowing, changes in taste, mouth sores, and fatigue. It's essential to discuss the risks and benefits with the treating oncologist to make the best treatment plan for each individual case. | 1,2 | 1,2 | Not applicable |
| 75. What are the advantages of transoral laser surgery compared to conventional open surgery? | Transoral laser surgery is a type of minimally invasive procedure performed using a laser through the mouth (transoral) to treat various conditions, most often those related to the throat or larynx. Here are several advantages of transoral laser surgery compared to conventional open surgery:   1. Less Invasive: Transoral laser surgery is less invasive than traditional open surgery, as it doesn't require large incisions. This can lead to less postoperative pain and discomfort. 2. Quicker Recovery: Patients typically experience a quicker recovery period and can often return to their daily routines faster than they would following open surgery. 3. Lower Risk of Infection: Due to the minimally invasive nature of transoral laser surgery, the risk of postoperative infection is generally lower. 4. Reduced Scarring: As there's no need for significant external incisions, the surgery results in minimal or no visible scarring. 5. Improved Precision: The use of lasers allows for higher precision in targeting and removing the affected tissues, which can be particularly beneficial when operating on sensitive areas like the throat or larynx. This can help to preserve the function of these areas. 6. Shorter Hospital Stay: Many transoral laser surgeries can be performed on an outpatient basis, meaning that the patient may not need to stay in the hospital overnight. 7. Reduced Blood Loss: Lasers cauterize as they cut, which can significantly reduce bleeding during surgery. 8. Preservation of Vocal Function: For surgeries involving the larynx or other parts of the vocal apparatus, transoral laser surgery can often preserve vocal function better than open surgery.   It's important to note that the advantages can vary based on the specific medical condition being treated and the individual patient's circumstances. As with any medical procedure, both transoral laser surgery and open surgery carry potential risks and complications, which should be discussed thoroughly with a healthcare provider. | Comparable | 1 | 1 | Not applicable |
| 76. How is photodynamic therapy used in treating head and neck cancer? | Photodynamic therapy (PDT) is a form of treatment that uses a drug, known as a photosensitizer, along with a particular type of light to kill cancer cells. This therapy is applied in the management of certain types of cancer, including head and neck cancer.  Here's how PDT is used in the treatment of head and neck cancers:   1. Prevention of Surgery or Radiotherapy: PDT can be used in early-stage cancers or pre-cancerous conditions, especially in areas that are hard to reach or where surgical intervention could cause significant disfigurement or functional loss. It is often considered for patients who are not good candidates for surgery or radiotherapy. 2. Palliative Treatment: For advanced stage cancers, where curative treatment might not be possible, PDT can be used to alleviate symptoms and improve quality of life. It can reduce the size of tumors, relieving symptoms such as pain, difficulty swallowing, or breathing problems. 3. Combination Treatment: PDT can be combined with other therapies such as chemotherapy, surgery, or radiation therapy. The rationale for this approach is that PDT may help to enhance the effectiveness of these other treatments.   The process typically involves the following steps:   - Administration of Photosensitizer: The photosensitizing agent is injected into the patient's bloodstream. The agent is absorbed by cells all over the body but stays in cancer cells longer than it does in normal cells. - Light Activation: A special type of light is then shone onto the tumor. This light activates the photosensitizer. - Cell Destruction: The activated photosensitizer converts oxygen into a form that can kill nearby cancer cells. It can also damage the blood vessels in the tumor, thereby preventing the cancer from receiving necessary nutrients.   One of the advantages of PDT is that it can precisely target cancer cells, minimizing damage to healthy tissues. However, one significant limitation is that the light needed to activate the photosensitizing agent can't penetrate deeply. Therefore, PDT is primarily used for tumors on or just under the skin or on the lining of internal organs or cavities.  Potential side effects include skin sensitivity to light, swelling, burning, and pain at the site of treatment. There may also be complications related to the photosensitizing agent, such as allergic reactions.  Despite its limitations, PDT continues to be an area of active research and its uses are being expanded as new technologies and photosensitizers become available. | Comparable | 1 | 1 | Not applicable |
| 77. What is the recommended treatment for T4N2M0 hypopharyngeal cancer with larynx invasion? | The treatment of T4N2M0 hypopharyngeal cancer with larynx invasion typically involves a multidisciplinary approach combining surgery, radiation therapy, and chemotherapy. The specific treatment plan may vary based on individual factors, such as the patient's overall health, age, and preferences, and should be determined by an oncology team.  Here is a general overview of the treatment options commonly used for this stage of hypopharyngeal cancer:   1. Surgery: Surgery plays a crucial role in the management of T4N2M0 hypopharyngeal cancer with larynx invasion. The primary goal is to remove the tumor while preserving as much normal tissue and function as possible. The surgical procedures may include total laryngectomy (removal of the larynx) and partial pharyngectomy (removal of a portion of the pharynx). Neck dissection may also be performed to remove lymph nodes in the neck. 2. Radiation therapy: Radiation therapy, either alone or in combination with surgery, is commonly used to treat hypopharyngeal cancer. It involves the use of high-energy X-rays or other types of radiation to destroy cancer cells and shrink tumors. In this case, both external beam radiation therapy (EBRT) and brachytherapy (internal radiation) may be used. EBRT targets the primary tumor site, regional lymph nodes, and potential areas of spread, while brachytherapy involves the placement of radioactive sources directly into the tumor site. 3. Chemotherapy: Chemotherapy drugs may be used in combination with radiation therapy (called chemoradiation) to enhance treatment effectiveness. Chemotherapy drugs can help kill cancer cells throughout the body and may be administered before or after surgery or along with radiation therapy. The specific chemotherapy drugs and regimens vary, but commonly used drugs include cisplatin, docetaxel, and fluorouracil.   It's important to note that treatment plans are individualized, and other factors such as the exact location and size of the tumor, the involvement of nearby structures, and the patient's overall health will also influence the treatment decisions. The treatment team, which may include oncologists, surgeons, radiation oncologists, and other specialists, will discuss and determine the most appropriate treatment approach for each patient. | Comparable | 1 | 1 | Not applicable |
| 78. How is cetuximab used in treating head and neck cancer? | Cetuximab is a type of targeted therapy known as a monoclonal antibody. This drug is designed to target and block the epidermal growth factor receptor (EGFR), a protein found on the surface of many types of cells, including cancer cells. When EGFR is activated, it triggers a series of chemical reactions inside the cell that promote cell growth and division, inhibit cell death, stimulate blood vessel growth, and enhance cell migration, all of which can contribute to tumor growth and metastasis.  Head and neck cancers often have high levels of EGFR, and blocking this receptor with cetuximab can help to control the growth and division of these cancer cells. In the context of head and neck cancer, cetuximab is used in the following ways:   1. **Locally or regionally advanced head and neck cancer:** Cetuximab is often used in combination with radiation therapy for the initial treatment of locally or regionally advanced head and neck squamous cell carcinoma (HNSCC). This is based on clinical trial results showing that cetuximab can enhance the effects of radiation therapy and improve patient outcomes. 2. **Recurrent or metastatic head and neck cancer:** Cetuximab can be used alone or in combination with chemotherapy for the treatment of recurrent or metastatic HNSCC. In this setting, cetuximab can help to control disease progression and extend patient survival.   Like all cancer treatments, cetuximab is associated with potential side effects, including skin problems (rash, itching, nail changes), infusion reactions, electrolyte imbalances, and others. It's important for patients receiving cetuximab to be closely monitored and for side effects to be managed appropriately.  As of my knowledge cutoff in September 2021, ongoing research is exploring the use of cetuximab in combination with other types of cancer treatments, such as immunotherapy, as well as its use in other types of cancer. For the most current information on this topic, I would recommend consulting recent medical literature or guidelines. | Comparable | 1 | 1 | Not applicable |
| 79. Is additional treatment necessary after surgery for T4N2M0 laryngeal carcinoma? | T4N2M0 laryngeal carcinoma is a relatively advanced form of laryngeal cancer, according to the TNM staging system used to classify the extent of cancer.  T4 refers to a tumor that has grown extensively into surrounding tissues, N2 indicates that the cancer has spread to multiple lymph nodes on one or both sides of the neck, and M0 denotes that there is no distant metastasis (spread to other organs).  The need for additional treatment after surgery often depends on several factors:   1. Margins of the surgical resection: If cancer cells are present at the edges of the removed tissue, it indicates that some cancer may have been left behind. This often necessitates additional treatment. 2. Extracapsular spread: If cancer has broken through the lymph node capsule, this is an adverse feature and may lead to consideration of post-operative radiation or chemo-radiation. 3. Positive nodes: With N2 disease, you have multiple positive nodes, so this would often result in consideration of radiation therapy to the neck post-operatively. 4. Pathological findings: Sometimes the pathology from the surgery may reveal adverse features, such as perineural invasion or vascular invasion. This may also lead to consideration of further treatment.   Typically, for advanced laryngeal cancer like T4N2M0, a multi-modal treatment approach is often recommended which could include a combination of surgery, radiation therapy, and chemotherapy.  Please note that each patient's situation is unique and the decision for additional treatment should be made after discussing the pros and cons with the treating physician and multidisciplinary team. | Comparable | 1 | 1 | Not applicable |
| 80. I have 2 cm low grade mucoepidermoid carcinoma in my parotid gland without any neck metastasis. What is the best treatment option? | Low-grade mucoepidermoid carcinoma of the parotid gland is typically treated with surgery. The type and extent of the surgery depend on the tumor's size and location within the gland. Surgical approaches can vary from a simple lumpectomy (removing just the tumor) to a total parotidectomy (removing the entire parotid gland).  Given the size of your tumor (2 cm) and the fact that it's low grade, it's possible that a more conservative surgical approach might be taken. However, this would ultimately be determined by your surgeon based on the tumor's exact location and the need to preserve the facial nerve, which is located near the parotid gland.  In many cases, low-grade mucoepidermoid carcinomas may not require radiation therapy, especially if the tumor is small and completely removed with surgery. However, if the tumor is high grade, incompletely removed, or there are other risk factors present, radiation therapy might be recommended after surgery.  Again, I strongly recommend discussing this with your healthcare provider. They'll be able to consider all the relevant factors and advise you on the most appropriate treatment approach for your individual situation. | Comparable | 1 | 1 | Not applicable |
| 81. Does the treatment for head and neck cancer affect swallowing? | Yes, treatments for head and neck cancer can often affect swallowing, a condition medically known as dysphagia. This is because these treatments might damage the structures involved in swallowing. Here are some of the main treatments for head and neck cancer that could lead to swallowing difficulties:   1. Surgery: Depending on the location and extent of the cancer, surgery may involve removing parts of the throat, larynx, or other structures involved in swallowing. This can affect your ability to swallow either temporarily or permanently. 2. Radiation therapy: This treatment can cause inflammation and scarring in the tissues of the throat, leading to swallowing problems. The severity and duration of these effects can vary. Some patients may experience only temporary dysphagia during or shortly after treatment, while others may have long-term or even permanent difficulties. 3. Chemotherapy: Some chemotherapy drugs can cause mouth sores and throat inflammation, leading to swallowing difficulties. However, these effects are typically temporary and improve once the treatment is completed. 4. Combined chemoradiation: This is a combination of chemotherapy and radiation therapy, often used for advanced head and neck cancers. It can have additive side effects from both treatments, potentially leading to more pronounced swallowing difficulties.   If you're dealing with head and neck cancer and are concerned about swallowing problems, it's crucial to discuss this with your healthcare team. Speech and swallowing therapists can often provide exercises and strategies to help manage these difficulties. Also, nutritionists can guide you on how to maintain your nutrition status if swallowing becomes difficult. | Comparable | 1 | 1 | Not applicable |
| 82. My neck dissection pathology report for larynx carcinoma showed incidental papillary thyroid cancer metastasis. Do i need additional treatment for this? | I'm an AI and can't provide medical advice, but I can tell you that incidental findings like papillary thyroid cancer metastasis in a pathology report often require further investigation and potential treatment. The decision to initiate additional treatment would largely depend on multiple factors such as:   1. The extent of the metastasis. 2. The overall health condition of the patient. 3. The patient's history and prior treatments. 4. The subtype of papillary thyroid carcinoma, as some are more aggressive than others.   It's also essential to understand that the incidental finding of metastatic papillary thyroid carcinoma might impact your overall prognosis and treatment plan for laryngeal carcinoma.  Typically, papillary thyroid carcinoma is treated with surgery to remove the thyroid (thyroidectomy) and often followed by radioactive iodine to treat any remaining cancer cells or to treat thyroid cancer that has spread to the lymph nodes or other parts of the body. But again, the need for these treatments will depend on your individual case.  I would highly recommend discussing your pathology results with your oncologist or endocrinologist to understand your options better. They will be able to provide the most suitable advice based on your full medical history and current health status. | Comparable | 1 | 1 | Not applicable |
| 83. Can treatment for head and neck cancer affect my speech? | Yes, treatment for head and neck cancer can certainly affect speech. The impact will depend on several factors, including the location and extent of the cancer, the specific treatment approach used, and the individual patient's condition.   1. Surgery: If the cancer is located in areas involved in speech production (like the throat, voice box, or tongue), surgical removal of the cancer could impact speech. The degree of impact can range from slight changes in voice quality to significant speech difficulties, depending on the extent of the surgery. 2. Radiation Therapy: Radiation therapy may cause inflammation and swelling in the treated areas, leading to temporary or permanent changes in voice quality and speech. Over time, it could also lead to fibrosis (a thickening and scarring of connective tissues), which may alter the flexibility and function of speech-related structures, further impacting speech. 3. Chemotherapy: While chemotherapy doesn't directly impact speech, it can cause mouth sores and inflammation in the mouth and throat, which could make speaking painful or difficult.   In many cases, patients undergoing treatment for head and neck cancer will work with a speech-language pathologist. These professionals can help patients adapt to changes in their speech or swallowing ability and can guide them through exercises to improve their function over time. In some cases, speech may improve or return to normal as the body heals from treatment. However, in other situations, changes to speech may be permanent, and adaptive strategies or devices may be necessary. | Comparable | 1 | 1 | Not applicable |
| 84. What is radioactive iodine therapy? | Radioactive iodine therapy is a treatment often used for thyroid cancer and some types of overactive thyroid conditions such as Graves' disease. This therapy uses radioactive isotopes, typically iodine-131, to target and destroy thyroid cells. The thyroid gland is unique in that it absorbs nearly all the iodine in the human body. This makes radioactive iodine an effective treatment for diseases of the thyroid.  The treatment involves the patient taking a capsule or liquid with enough radioactive iodine to damage or kill the thyroid cells. The radioactive iodine is absorbed by the thyroid gland where it emits radiation that, over a few weeks to a few months, destroys the gland.  For cancer, the goal is to kill the thyroid cells because they are malignant. If the entire thyroid is removed through surgery due to cancer, radioactive iodine may still be used to destroy any remaining thyroid tissue and to treat any thyroid cancer that has spread to the lymph nodes or other parts of the body.  In hyperthyroid conditions, the therapy aims to restore normal thyroid hormone levels by reducing the size of the thyroid or lowering its activity.  While it's generally considered safe, there can be side effects to radioactive iodine treatment. These can include dry mouth, neck tenderness, and temporary alterations in taste or smell. A decrease in tear production, which can cause dry eyes, is another potential side effect. It's also important to limit contact with others for a certain amount of time after receiving the treatment due to the body's radiation levels.  As with any medical treatment, the risks and benefits should be discussed with a healthcare provider. It is especially important for women who are pregnant or nursing, or those trying to conceive, to discuss this with their provider, as radioactive iodine can harm a fetus or nursing infant. | Comparable | 1 | 1 | Not applicable |
| 85. What is induction chemotherapy? | Induction chemotherapy, also known as first-line or primary chemotherapy, refers to the first series of chemotherapy treatments given to patients diagnosed with cancer. Its primary goal is to cause a remission, or decrease the size of the tumor, before subsequent treatments.  Induction chemotherapy can serve several purposes:   1. Shrinkage of the tumor to make surgery or radiotherapy more effective. 2. To test the sensitivity of the cancer to the chemotherapy drugs. 3. To reduce symptoms and improve quality of life. 4. To control the disease and prolong life in situations where cure isn't possible.   This treatment often involves a combination of chemotherapy drugs to increase effectiveness and is typically administered in cycles, with a period of treatment followed by a period of rest to allow the body to recover. The specific drugs, dosage, and treatment schedules vary depending on the type of cancer, its stage, and the overall health of the patient.  Remember, any treatment plan should be discussed in detail with your healthcare provider, who is best equipped to provide guidance based on your unique situation and medical history. | Comparable | 1 | 1 | Not applicable |
| 86. What is the treatment for stage 1 maxillary sinus cancer? | The treatment of stage 1 maxillary sinus cancer usually involves a combination of surgery and radiation therapy.   1. Surgery: The goal of surgery in stage 1 maxillary sinus cancer is to completely remove the tumor and any involved tissues while preserving function and appearance as much as possible. The specific surgical procedure may vary depending on the exact location and size of the tumor. In some cases, endoscopic surgery may be possible, which involves the use of a thin tube with a camera and light at the end (endoscope) to remove the tumor through the nose. In other cases, a more invasive procedure might be needed. 2. Radiation Therapy: Radiation therapy may be used after surgery to kill any remaining cancer cells, to reduce the risk of recurrence. It can also be used as the primary treatment in cases where surgery isn't an option due to the tumor's location or the patient's health.   Chemotherapy is typically reserved for more advanced cases or when the cancer has spread to other areas, but it could be considered in some situations. Each patient's treatment plan is individualized and based on many factors, including the exact location and size of the tumor, the patient's overall health, and the patient's preferences.  Immunotherapy and targeted therapy are newer types of cancer treatment that might be used in some cases, though as of my last training cut-off in September 2021, these are more commonly used in more advanced or recurrent cases of cancer.  It's crucial that any person diagnosed with maxillary sinus cancer consult with a multidisciplinary team of cancer specialists, including a head and neck surgeon, radiation oncologist, medical oncologist, and others to determine the best treatment plan for them. | Comparable | 1 | 1 | Not applicable |
| 87. What are the reconstruction options following a segmental mandibulectomy? | Segmental mandibulectomy is a procedure that involves the removal of a part of the mandible (lower jawbone), typically due to conditions like cancer. Reconstruction of the mandible after segmental mandibulectomy is crucial for maintaining oral functions and aesthetics. Here are the main types of reconstruction options:   1. Autografts: This is a procedure where bone and tissue are taken from another part of the patient's own body to reconstruct the jaw. The most common autografts come from the fibula (leg bone), scapula (shoulder blade), or iliac crest (hip bone). The blood vessels of these grafts are then anastomosed (connected) to the vessels in the neck to keep the graft alive. 2. Allografts: This is the use of bone and tissue from another human being to rebuild the mandible. Allografts are less common due to the risk of immune rejection. 3. Xenografts: These are grafts from other species, such as bovine bone grafts. Like allografts, they are also less commonly used due to potential issues with immune compatibility. 4. Synthetic materials: Various types of synthetic materials can be used to reconstruct the mandible. These include metals, ceramics, and polymers. Titanium plates are commonly used to maintain the shape and function of the jaw. 5. Tissue engineering and bone regeneration: This is a newer area of research, where scientists are trying to grow bone and soft tissue in the lab using stem cells. Although it's a promising area, as of my knowledge cutoff in 2021, it is not yet a commonly available treatment. 6. Vascularized Free Flap Reconstructions: This technique involves transferring a composite flap of bone, muscle, and skin from a distant site along with its blood supply for the reconstruction of complex defects. For mandible reconstruction, the fibula free flap is commonly used due to the length of bone available and the possibility of a two-team approach. 7. Nonvascularized Bone Grafts: These grafts are primarily used in smaller defects and involve bone grafts that are not connected to a blood supply. However, the lack of a vascular supply limits their use to smaller defects. 8. Distraction Osteogenesis: This method stimulates the body's natural ability to grow bone by gradually separating the existing bone with a distraction device. This technique is generally utilized in pediatric patients, or in patients where other traditional methods are not viable. 9. Prosthetic rehabilitation: After surgical reconstruction, dental implants and prostheses may be used to replace missing teeth and improve function and aesthetics.   The most appropriate reconstruction method can vary significantly depending on factors like the size and location of the mandibular defect, the patient's overall health status, the patient's personal preferences, and the surgical team's expertise. This decision should be made in collaboration with a team of specialists, including oral and maxillofacial surgeons, plastic surgeons, prosthodontists, and oncologists. | Comparable | 1 | 1 | Not applicable |
| 88. Should I remain at home after undergoing radioactive iodine therapy for thyroid cancer? | After receiving radioactive iodine (RAI) therapy, you will be radioactive for a period of time and you could potentially expose other people to radiation. To protect those around you, certain precautions are recommended.  Most guidelines suggest limiting close physical contact, especially with children and pregnant women, for a period after the treatment. This is because they are more sensitive to the effects of radiation. The length of time these precautions should be followed varies based on the dose of RAI you received, and can be anywhere from a few days to a week or more.  Staying at home can be a way to limit exposing other people to radiation, but you don't have to be completely isolated. You might just need to stay in a separate room and use separate household items. You'll also be instructed on how to handle bodily waste, as it can contain radiation.  Other general precautions may include:   1. Maintaining a certain distance from people for a specified period of time. 2. Sleeping in a separate bed from your partner. 3. Limiting time in public places and using public transport. 4. Washing your clothing and bed linens separately. 5. Using separate towels, washcloths, and utensils until they can be thoroughly cleaned.   Remember that these precautions are temporary, and you can return to your normal activities once the radiation in your body has fallen to safe levels.  However, these are just general suggestions. Each person's situation is unique and you should get instructions tailored to your case from your healthcare provider. | Comparable | 1 | 1 | Not applicable |
| 89. My pathology report following total laryngectomy and bilateral neck dissection shows that i have T4 larynx cancer without lymphatic spread to my neck. Is additional treatment necessary for my neck? | I'm an AI and not a doctor, but I can provide some general guidance on the topic based on what's typically done in such scenarios. However, you should consult your own healthcare provider for personalized advice.  A T4 cancer is a fairly advanced stage of cancer, signifying that the tumor has grown substantially. The fact that it hasn't spread to your lymph nodes (as indicated by the bilateral neck dissection) is generally a good sign in terms of prognosis.  Given that you've already undergone a total laryngectomy and bilateral neck dissection, the next step would typically involve adjuvant treatment based on the specific characteristics of your tumor and your overall health status. This could potentially include radiation therapy, chemotherapy, or both.  Radiation therapy may be used to kill any remaining cancer cells that might be present around the surgical site. Similarly, chemotherapy might be used to target any potential microscopic cancer cells that could be present elsewhere in the body.  Your doctor might also consider other factors such as the surgical margin status, the presence of perineural invasion or lymphovascular invasion, and the presence of extracapsular spread (even if your neck lymph nodes are negative), as these could influence the decision to recommend further treatment.  Again, you should discuss this with your own healthcare provider as they have the full context of your health status and the specifics of your pathology report. | Comparable | 1 | 1 | Not applicable |
| 90. What are the indications for neck dissection in clinically N0 paranasal sinus cancer? | A clinically N0 designation means there is no clinical or radiographic evidence of regional lymph node metastasis. In general, neck dissection is a surgical procedure for control of neck disease (metastatic lymph nodes) in various head and neck cancers.  Paranasal sinus cancers, in general, are less likely to metastasize to the cervical lymph nodes compared to other head and neck malignancies, particularly in the early stages, but the risk is not zero.  Here are some of the potential indications for considering a neck dissection in clinically N0 paranasal sinus cancer:   1. Histopathological features: Certain histological types of cancer, such as squamous cell carcinoma or undifferentiated carcinoma, tend to have a higher incidence of cervical metastases, and therefore may warrant consideration for neck dissection. 2. Tumor size and extent: Even in clinically N0 paranasal sinus cancer, if the primary tumor is large or invasive, there is a greater risk of occult (hidden) lymph node metastasis. 3. Perineural or lymphovascular invasion: These are indicators of a higher risk for metastatic spread and therefore might warrant a neck dissection. 4. High-risk locations: Some areas in the paranasal sinus are associated with higher risk of cervical lymph node metastasis, such as the maxillary sinus. 5. Positive surgical margins: If the cancer is not completely removed during surgery, there's a higher risk for metastatic spread. 6. Patient's overall health status: The patient's general health and ability to withstand surgery will also factor into the decision.   However, it's important to note that the decision to perform a neck dissection in clinically N0 paranasal sinus cancer should be made on a case-by-case basis, taking into account various factors including but not limited to those listed above. In many cases, close observation and follow-up, including regular imaging studies, may be appropriate. The decision should be made in collaboration with the patient and based on a thorough discussion of the potential benefits, risks, and alternatives.  Finally, any new research or clinical guidelines published after my knowledge cutoff in September 2021 could potentially alter these recommendations. | Neck dissection is a surgical procedure that's often used to treat cancer that has spread to the lymph nodes in the neck. In the context of paranasal sinus cancer, the disease often spreads directly to adjacent tissues and structures rather than via the lymphatic system. Hence, the routine use of neck dissection is generally not recommended unless there is clinical or radiologic evidence of regional metastasis.  However, some clinicians may recommend elective neck dissection in patients with advanced paranasal sinus cancer (stage T3 or T4) due to a higher likelihood of occult metastasis.  The specific indications of neck dissection in clinically N0 paranasal sinus cancer might include:   1. Advanced tumor stage: Higher T stage (T3 or T4) tumors have a greater propensity for metastasis. 2. High-risk histology: Certain histological types, like undifferentiated carcinoma, adenocarcinoma, or squamous cell carcinoma, may have a higher risk of lymph node metastasis. 3. Involvement of certain anatomical sites: Tumors involving the maxillary sinus, especially the posterior wall or the infratemporal fossa, are more likely to metastasize to the neck nodes. 4. Perineural or perivascular invasion: These features, if present in the primary tumor, can increase the risk of neck metastasis. 5. Suspicion of microscopic disease: Sometimes, despite imaging studies showing no overt neck disease (N0), there may be a clinical suspicion of microscopic disease, for instance, in large, high-grade tumors. 6. Planned radiation therapy to the neck: If postoperative radiation is planned to the neck, some clinicians may prefer to perform an elective neck dissection to lower the radiation dose and potentially decrease the radiation-related side effects.   Remember, the decision for neck dissection should always be made based on a careful discussion between the patient and the treating team, weighing the risks and benefits of the procedure in the context of the overall treatment plan and patient's preferences. The guidelines and recommendations can vary, and newer research or protocols might have emerged after my last update in September 2021. | 2,1 | 2,1 | Not applicable |
| ***RECOVERY, OPERATIVE RISKS, COMPLICATIONS and FOLLOW-UP*** |  |  |  |  |  |
| 91. How common is shoulder dysfunction after a neck dissection operation? | Shoulder dysfunction is a relatively common complication after neck dissection surgeries, which include procedures like radical neck dissection, modified radical neck dissection, and selective neck dissection. Neck dissection can potentially lead to issues with the spinal accessory nerve, which is important for shoulder function.  Studies conducted prior to my last update in 2021 reported varying rates of shoulder dysfunction post neck dissection, with some studies suggesting that as many as 50% or more patients could experience some level of shoulder dysfunction following surgery. Symptoms might include shoulder pain, limited range of motion, and weakness, which can impact quality of life.  It's crucial to approach these numbers with some caution. Factors such as how shoulder dysfunction is defined and measured, the length of the follow-up period, and the specifics of patient care post-surgery can significantly influence these rates.  Patients should discuss potential risks and complications with their healthcare provider before any surgical procedure. If shoulder dysfunction occurs after neck dissection, physical therapy may be recommended to improve shoulder mobility and strength. | Comparable | 1 | 1 | Not applicable |
| 92. How is shoulder dysfunction treated in patients with head and neck cancer? | Head and neck cancer patients often experience shoulder dysfunction as a result of the disease itself or as a side effect of surgical, radiation, or chemotherapy treatment. The shoulder dysfunction may be due to muscle weakness, stiffness, pain, or neuropathy, particularly when nerves such as the spinal accessory nerve or the brachial plexus are impacted. The treatment options for shoulder dysfunction in these patients include:   1. Physical therapy: This is the first line of treatment and often continues long-term. Physical therapy aims to improve shoulder function, reduce pain, and increase the range of motion. Specific exercises and stretches can help strengthen the shoulder and improve mobility. 2. Occupational therapy: This can help the patient adapt to daily activities that may be difficult due to shoulder dysfunction, using tools or techniques to make tasks easier. 3. Pain management: Non-steroidal anti-inflammatory drugs (NSAIDs), acetaminophen, or stronger painkillers can be used, as necessary. It's essential to manage pain effectively to enable participation in physical and occupational therapy. 4. Radiation therapy: Radiation fibrosis syndrome (RFS) can cause shoulder dysfunction. It's treated with a combination of physical therapy and medication, sometimes alongside therapeutic low-dose radiation. 5. Surgery: In some cases, surgery may be necessary to correct the dysfunction, especially if it's due to a tumor pressing on the nerves or if there are significant structural changes following treatment. 6. Neurostimulation techniques: Techniques like Transcutaneous Electrical Nerve Stimulation (TENS) may be useful in managing pain associated with neuropathy. 7. Alternative therapies: Therapies such as acupuncture, massage, and mindfulness-based stress reduction can also contribute to pain management and improve function. 8. Education: Patients and their caregivers need to be educated about the nature of the dysfunction, how to manage it, and the importance of sticking with the rehabilitation program. 9. Nutrition and general health: Encouraging a balanced diet and overall healthy lifestyle can aid in recovery and improve the general well-being of patients.   These approaches are often combined and personalized to fit the needs of each patient. The overall goal of treatment is to improve the patient's quality of life by reducing pain and improving shoulder function. Coordination among a multidisciplinary team of physicians, nurses, physical therapists, occupational therapists, and others is crucial for effective treatment. | Comparable | 1 | 1 | Not applicable |
| 93. How common is cognitive impairment among patients with head and neck cancer?? | Cancer and cancer treatments can impact cognitive function through various mechanisms. For instance, chemotherapy is associated with a phenomenon sometimes referred to as "chemo brain," which can lead to cognitive impairment. Radiation therapy, particularly when administered to the brain, can also have cognitive effects.  Furthermore, head and neck cancers may sometimes directly affect brain function if they spread to the brain or if they impact structures critical to cognitive function, such as cranial nerves.  Lastly, psychological factors like depression and anxiety, which are common among cancer patients, can also impact cognitive function.  However, it's essential to keep in mind that research on this topic is ongoing, and our understanding of the connection between different types of cancer (including head and neck cancer) and cognitive impairment is evolving.  For the most current information, I would recommend consulting recent research studies or speaking with healthcare professionals who specialize in this field. | Comparable | 2 | 2 | Not applicable |
| 94. What are the sign and symptoms of hypothyroidism after radiation therapy for head and neck cancer? | Radiation therapy to the head and neck area, particularly when it involves the thyroid gland or nearby structures, can increase the risk of developing hypothyroidism. Hypothyroidism is a condition where the thyroid gland does not produce enough thyroid hormones, leading to a slowdown in many of the body's functions. The symptoms are often subtle and can be mistaken for normal aging.  If you've had radiation therapy to your head and neck and are concerned about hypothyroidism, here are some symptoms to look out for:   1. Fatigue and weakness: People with hypothyroidism often report feeling tired, sluggish, or weak. 2. Depression: Hypothyroidism is often associated with mood changes, including depression. 3. Sensitivity to cold: People with this condition often feel unusually cold. 4. Unexplained weight gain or difficulty losing weight: Despite maintaining the same diet and exercise habits, you might find yourself gaining weight or having difficulty losing it. 5. Dry skin and hair: Your skin may become dry and rough, and your hair can become coarse, dry, brittle, or thin. 6. Hair loss: This is especially noticeable on the scalp, where it can be quite dramatic. 7. Slow heart rate (bradycardia): A slower than normal heart rate is another potential sign. 8. Constipation: Problems with digestion are common in hypothyroidism. 9. Memory problems or having trouble thinking clearly: Some people with hypothyroidism complain of brain fog or difficulty concentrating. 10. Menstrual irregularities: Women can experience changes in their menstrual cycle, including heavier periods, more frequent periods, or infertility. 11. Joint or muscle pain: Some people with hypothyroidism experience general aches and pains in the muscles and joints. 12. Hoarseness or changes in voice: This can be a symptom of thyroid problems, especially in those who have had radiation therapy to the head and neck.   These symptoms can also be caused by a variety of other conditions, and having these symptoms does not necessarily mean that you have hypothyroidism. If you're experiencing any of these symptoms, especially after receiving radiation therapy to your head or neck, it's important to consult with your healthcare provider for a proper diagnosis and treatment. A simple blood test can help confirm whether you have hypothyroidism. | Comparable | 1 | 1 | Not applicable |
| 95. What oral complications and dental decay can occur in survivors of head and neck cancer? | Head and neck cancer and its treatments can have significant impacts on oral health and can lead to a variety of complications. Some of the common oral complications that may be experienced by head and neck cancer survivors include:   1. Xerostomia or Dry Mouth: This is the most common oral complication for head and neck cancer survivors. Many treatments, particularly radiation therapy to the head and neck, can damage the salivary glands, leading to a decrease in saliva production. This can cause difficulties in chewing and swallowing, taste alterations, and an increased risk for dental decay and oral infections. 2. Radiation Caries or Dental Decay: Radiation caries is a rapidly progressing and severe form of tooth decay that can occur following radiation therapy. The decreased saliva production resulting from damaged salivary glands creates an environment conducive to the proliferation of cavity-causing bacteria. 3. Trismus or "Lockjaw": Trismus is a condition characterized by reduced ability to open the mouth due to muscle or joint stiffness, often resulting from radiation therapy or surgical treatment. This can limit oral function and make dental care challenging. 4. Osteoradionecrosis: This is a condition where the bone tissue dies due to radiation, causing severe damage. The lower jaw, which has less blood supply compared to other bones, is particularly susceptible. It can lead to pain, infection, fracture, and difficulty in healing. 5. Mucositis: This is inflammation and ulceration of the mucous membranes in the mouth, which can occur during chemotherapy or radiation therapy. It can cause pain, difficulties with eating, and increase the risk of infection. 6. Taste Changes: Many patients experience changes in taste, especially during chemotherapy or radiation treatment. For some, this change can be permanent. 7. Oral Infections: Decreased saliva production, damage to the mucosal lining of the mouth, and a weakened immune system due to cancer treatments can lead to an increased risk of oral infections like thrush and periodontal disease.   To manage and prevent these complications, head and neck cancer survivors are recommended to maintain good oral hygiene, regularly visit their dentist, and stay hydrated to alleviate dry mouth symptoms. Fluoride treatments can help prevent radiation caries, while physical therapy exercises can be useful for managing trismus. For severe complications like osteoradionecrosis, surgical intervention might be needed. | Comparable | 1 | 1 | Not applicable |
| 96. How common is radiation fibrosis among patients with head and neck cancer? | Radiation fibrosis is a common long-term side effect of radiation therapy for cancer, including head and neck cancer. Radiation fibrosis syndrome (RFS) may include symptoms such as tissue fibrosis (hardening), pain, decreased range of motion, skin changes, and even dysfunction of specific organs.  The actual incidence can vary, depending on the population being studied, the radiation technique used, the specific area of the body irradiated, and the follow-up time. It also depends on the dose of radiation received and the individual's biological response to radiation.  As of my knowledge cutoff in September 2021, reported rates of radiation fibrosis in head and neck cancer patients may range broadly, from 5 to 50%. This wide range is due to the reasons mentioned above. Also, not all cases of radiation fibrosis are symptomatic or severe, and some might only be detected through radiological examination or biopsy, making incidence rates difficult to quantify accurately.  However, with advancements in radiation therapy techniques, like intensity-modulated radiation therapy (IMRT) and volumetric modulated arc therapy (VMAT), the risk of radiation-induced side effects has been significantly reduced. These techniques allow more precise targeting of the tumor, sparing surrounding healthy tissue from unnecessary radiation exposure.  If you are seeking more current data or data specific to a particular type of head and neck cancer, you should consult recent scientific literature or a medical professional. | As of my knowledge cut-off in September 2021, research suggested that a significant proportion of head and neck cancer patients who undergo radiation therapy may develop radiation fibrosis. The incidence is highly variable, depending largely on the exact area treated, the radiation dose, the specific technique used, and the patient's individual response.  Radiation fibrosis refers to the scarring or hardening of tissues that occurs after radiation therapy. This can lead to various complications, depending on the location and extent of the fibrosis. In the head and neck region, for example, fibrosis can lead to issues with swallowing, voice changes, and lymphedema, among other problems.  For a more accurate and up-to-date estimate of the prevalence of radiation fibrosis in head and neck cancer patients, I would recommend consulting recent peer-reviewed literature or reaching out to a healthcare professional who specializes in oncology. They can provide the most current and accurate information based on the latest research and clinical guidelines. | 1,2 | 1,1 | 1,1 |
| 97. How is dry mouth treated in patients with head and neck cancer? | Dry mouth, also known as xerostomia, is a common side effect in head and neck cancer patients, particularly for those undergoing radiation therapy. Radiation can damage the salivary glands, leading to reduced saliva production, which can cause dry mouth.  Here are some strategies to manage dry mouth in these patients:   1. Medication: Certain medications, such as pilocarpine and cevimeline, may help stimulate saliva production. 2. Artificial Saliva: Over-the-counter artificial saliva products can also help to alleviate dry mouth symptoms. They are available in various forms including sprays, gels, and rinses. 3. Good Oral Hygiene: Regular and thorough oral care is essential. This involves brushing with a soft-bristled toothbrush, flossing daily, and regular dental checkups. 4. Hydration: Regularly sipping water or sucking on ice chips can help keep the mouth moist. 5. Diet Changes: Avoiding irritants such as alcohol, caffeine, and tobacco, and spicy or acidic foods, which can worsen dry mouth. Foods and drinks that stimulate saliva production, such as sugar-free gum or candies, can be helpful. 6. Humidifier: Using a humidifier in the bedroom can help to moisten the air and potentially relieve the symptom of dry mouth, especially during sleep. 7. Special Mouthwashes and Toothpastes: Using specially formulated mouthwashes and toothpastes designed for dry mouth may help. These are often free of alcohol and other ingredients that may exacerbate dry mouth. 8. Salivary Stimulators: Certain devices, such as salivary stimulators, can help increase saliva production. These devices use mild electrostimulation to promote saliva production.   In some severe cases, if these approaches are not sufficient, there are surgical options. For example, a procedure known as salivary duct rerouting can be used to redirect the flow of saliva from the remaining salivary glands towards the mouth.  The right strategy or combination of strategies for each patient will depend on the specific details of their health status, the nature of their cancer, the treatment they're receiving, and their overall needs and preferences. As always, any treatment decisions should be made in consultation with their healthcare providers. | Comparable | 1 | 1 | Not applicable |
| 98. How common is osteoradionecrosis among patients with head and neck cancer? | As of my last update in September 2021, osteoradionecrosis (ORN) of the jaw, a complication often associated with radiation therapy in the treatment of head and neck cancer, occurred in an estimated 2% to 22% of patients who had received radiation therapy, depending on the source of the information and the exact population studied. The wide range of reported incidence reflects the different definitions and diagnostic criteria of ORN, the varied radiation techniques used, the different types of cancer, and the different follow-up times and patient populations.  ORN is most commonly associated with radiation therapy for oral or oropharyngeal cancers. It's less common with modern radiation techniques such as intensity-modulated radiation therapy (IMRT) compared to older forms of radiation therapy.  Please consult a healthcare professional or current literature to get the most accurate and up-to-date information, as my knowledge was last updated in 2021 and advancements in medicine are ongoing. | Comparable | 1 | 1 | Not applicable |
| 99. How common are body image concerns among patients with head and neck cancer? | Head and neck cancers often involve surgery, radiation, and chemotherapy, which can result in visible physical changes such as scars, facial asymmetry, or changes to the neck or throat. These changes, along with potential speech and swallowing difficulties, can lead to significant body image concerns and can impact a patient's quality of life.  Moreover, these body image concerns can lead to psychological issues such as depression, anxiety, and social isolation, and may also impact the patient's interpersonal relationships. It's important that these psychosocial aspects are addressed along with the medical treatment, and many cancer treatment centers have support services in place, including psychology and social work, to help patients cope with these challenges.  To get the most current and accurate information, it would be best to look for recent studies or reviews in the medical literature that specifically address body image concerns among head and neck cancer patients. This is an active area of research and the understanding of these issues continues to evolve. | There is a considerable amount of research showing that body image concerns are common among patients who are diagnosed with various forms of cancer, including head and neck cancer.  Head and neck cancer, in particular, can have significant physical and functional impacts on patients, given the location of the disease. The treatments, which often include surgery, radiation, and chemotherapy, can cause disfigurement, changes in speech, difficulty swallowing, and other physical changes that can greatly affect a person's appearance and daily functioning.  These physical changes can often lead to psychological distress and body image concerns. Patients may feel self-conscious about their appearance, experience social anxiety, or avoid social interactions entirely. This can negatively affect their quality of life and overall wellbeing.  In a study published in the Journal of Clinical Oncology in 2012, it was found that body image disturbance was present in about 50% of head and neck cancer patients in their sample. Although this percentage may vary depending on the specifics of the population and method of assessment, it illustrates the potential magnitude of the problem.  Additionally, factors such as age, gender, and the specific type and location of cancer can affect the likelihood of body image concerns. For instance, younger patients and women tend to report higher levels of body image concern.  It's important to note that not all individuals with head and neck cancer will experience significant body image concerns, and psychological resilience varies greatly. Also, there are resources available to help manage these concerns, including counseling, support groups, and potentially reconstructive surgery.  Please consult your healthcare provider or a licensed mental health professional for personalized advice if you or someone you know is dealing with body image concerns related to cancer or its treatment. | 2,1 | 2,1 | Not applicable |
| 100. What are the signs/symptoms of sexual dysfunction in patients with head and neck cancer? | Sexual dysfunction in head and neck cancer patients can be a result of the psychological and physical stressors associated with diagnosis and treatment.  Here are some signs and symptoms that could indicate sexual dysfunction in these patients:   1. Physical Changes: Cancer treatments can cause various physical changes that could lead to sexual dysfunction. These changes can include fatigue, loss of desire, pain during sexual activity, or impotence. Some treatments may also lead to premature menopause in women, which can cause vaginal dryness and discomfort during intercourse. 2. Psychological Changes: The psychological stress of a cancer diagnosis and treatment can also contribute to sexual dysfunction. Anxiety, depression, and low self-esteem can all negatively affect a person's sexual desire and performance. 3. Changes in Body Image: Treatments for head and neck cancer can lead to visible changes in a person's appearance, like surgical scars, weight loss, or changes in the shape or structure of the face and neck. These changes can affect a person's body image and confidence, which can lead to sexual dysfunction. 4. Changes in Relationship Dynamics: The stress of a cancer diagnosis and treatment can also impact relationship dynamics, which can in turn affect sexual function. For example, a partner may become more of a caregiver, which can alter the dynamics of the sexual relationship. 5. Medication Side Effects: Many medications used in the treatment of cancer can have side effects that can lead to sexual dysfunction. For instance, some medications can cause fatigue, decrease sexual desire, or cause erectile dysfunction. 6. Neurological Changes: Some treatments for head and neck cancer can cause damage to the nerves, which can lead to sexual dysfunction. This can include issues like erectile dysfunction in men or a decrease in sexual pleasure in both men and women.   If you or someone else is experiencing these symptoms, it's important to speak with a healthcare provider. They can provide advice on managing these symptoms and can refer to specialists such as psychosexual therapists, counselors, or physiotherapists who can provide further help. | Comparable | 1 | 1 | Not applicable |
| 101. What are neurosensory disorders in patients with head and neck cancer? | Head and neck cancers, including cancers of the oral cavity, pharynx, larynx, and salivary glands, are a diverse group of cancers that can result in a wide array of symptoms and complications. Neurosensory disorders are one such potential complication, with various presentations depending on the location of the cancer, the treatment modalities used, and individual patient characteristics.  Here are some of the neurosensory disorders that may be observed in head and neck cancer patients:   1. Neuropathic Pain: This can occur due to the infiltration of cancer into peripheral nerves or from the damage caused by treatments such as surgery, radiation, or chemotherapy. 2. Dysphagia: Difficulty in swallowing can occur due to impairment in neural control of muscles involved in swallowing or damage to these muscles due to surgery or radiation. 3. Trigeminal Neuralgia: This involves severe facial pain due to the dysfunction of the trigeminal nerve, which may occur if a cancer invades the base of the skull or due to surgical or radiation damage. 4. Dysphonia: Changes or loss in voice can occur if the laryngeal nerves are affected either by the cancer itself or due to surgical or radiation damage. 5. Facial Paralysis: This can occur if the facial nerve is damaged or compressed by a tumor or due to surgical or radiation damage. 6. Hearing Loss and Tinnitus: These can occur if the cancer or treatment affects the inner ear or auditory nerve. 7. Dysgeusia: Altered taste sensation can occur due to damage to the taste buds from chemotherapy or radiation or due to dysfunction of the glossopharyngeal nerve or chorda tympani, which are involved in taste sensation. 8. Xerostomia: This is dry mouth caused by damage to the salivary glands from radiation therapy, resulting in reduced salivary production.   Management of these neurosensory disorders can be complex and may involve a multidisciplinary team including otolaryngologists, oncologists, speech and swallowing therapists, audiologists, and pain management specialists. | Comparable | 1 | 1 | Not applicable |
| 102. What are the survival rates for oral cavity and oropharyngeal cancer? | The survival rates for oral cavity and oropharyngeal cancer can vary depending on several factors, including the stage of cancer at diagnosis, the specific location within the oral cavity or oropharynx, the individual's overall health, and the treatment received. Survival rates are typically expressed as the percentage of people who survive for a certain period of time after diagnosis. It's important to note that these statistics are general estimates and may not reflect an individual's specific prognosis.  Here are the estimated 5-year survival rates for oral cavity and oropharyngeal cancer based on the American Cancer Society's data:   1. Oral Cavity Cancer:    - Localized (cancer is confined to the primary site): The 5-year survival rate is around 83%.    - Regional (cancer has spread to nearby lymph nodes or tissues): The 5-year survival rate is about 64%.    - Distant (cancer has spread to distant organs or lymph nodes): The 5-year survival rate is approximately 39%. 2. Oropharyngeal Cancer:    - Localized: The 5-year survival rate is around 69%.    - Regional: The 5-year survival rate is about 51%.    - Distant: The 5-year survival rate is approximately 39%.   It's worth mentioning that advancements in treatment and improvements in early detection can have a positive impact on survival rates. Additionally, individual cases may vary, and it's important to consult with a healthcare professional for personalized information and prognosis based on specific circumstances. | As of my last update, the 5-year survival rates for oral cavity and oropharyngeal cancers were dependent on the stage at diagnosis:   1. Localized (no sign that the cancer has spread outside of the organ it started in): About 84% 2. Regional (cancer has spread to nearby structures or lymph nodes): About 66% 3. Distant (cancer has spread to distant parts of the body): About 39%   These are estimates, and actual survival rates can vary depending on a number of factors including a patient's overall health, response to treatment, and more.  For the most recent data, I recommend checking resources such as the American Cancer Society, or seeking information from a medical professi | 1,2 | 1,2 | Not applicable |
| 103. Will surgery on my larynx affect my ability to speak? | As an AI developed by OpenAI, I can tell you that the outcome of larynx surgery can vary widely depending on the specific type of surgery, the reason for the surgery, and the individual patient's health status. Here are some general insights:   1. Voice Quality Changes: Many types of larynx surgeries can lead to changes in voice quality. This is particularly true for surgeries involving the vocal cords themselves. The voice may become hoarse, weak, or breathy. In some cases, these changes may be temporary as the larynx heals; in other cases, they may be more permanent. 2. Speech Therapy: After surgery, many patients may benefit from working with a speech-language pathologist. These professionals can help with exercises and techniques to improve voice quality and control after surgery. 3. Significant Impacts: In more severe cases, such as laryngectomy (removal of the larynx) often done to treat laryngeal cancer, the patient will lose their natural voice. These individuals will need to learn new ways of speaking, such as using an electrolarynx, esophageal speech, or a surgically constructed voice box.   Remember, it's critical that you discuss with your healthcare provider or surgeon about your particular case. They can provide you with the most accurate and personalized information based on your health status and the type of surgery you are having. This is general information and may not perfectly apply to your case. | Comparable | 1 | 2 | 2 |
| 104. Can treatment for oral cancer affect speech or swallowing? | Yes, both speech and swallowing can potentially be affected by oral cancer itself, as well as by its treatments. The extent to which these functions are affected depends on the location and size of the tumor, the specific treatment approach, and the individual patient's health and resilience.   1. Speech: The oral cavity plays a crucial role in speech. Structures such as the tongue, cheeks, and palate are all involved in the articulation of sounds. Oral cancer, particularly if it is in these areas, can cause difficulty speaking. Surgical treatment of oral cancer might also affect speech, particularly if it involves the removal of part of the tongue or other structures involved in articulation. Speech therapy is often needed after surgery to help patients regain their speaking abilities as much as possible. 2. Swallowing: Oral cancer can also affect swallowing, particularly if the cancer is located in areas involved in the swallowing process, such as the tongue or throat. Treatment such as surgery, radiation therapy, or chemotherapy can also cause difficulties with swallowing. Surgery might lead to structural changes that affect swallowing, while radiation and chemotherapy can cause side effects like mouth sores or dry mouth that make swallowing painful or difficult. Speech and swallowing therapists often work with patients to help manage these side effects and improve their swallowing function.   These potential effects on speech and swallowing are important considerations when planning treatment for oral cancer. The treatment team, which often includes doctors, speech-language pathologists, dietitians, and other health care providers, works together to find the most effective treatment plan that minimizes these effects as much as possible and supports the patient in managing any changes in speech or swallowing. | Comparable | 1 | 1 | Not applicable |
| 105. If my cancer has been completely eliminated, do i still need to come for follow-ups? | While it's great news that your cancer has been completely eliminated, it's still highly recommended that you continue regular follow-up appointments with your healthcare provider. There are several reasons for this:   1. Monitoring for recurrence: Even after successful treatment, there's a chance that cancer cells could remain in the body and cause the cancer to return, or recur. Regular follow-up appointments allow your doctors to check for signs of recurrence. 2. Secondary cancers: Sometimes, treatment for one type of cancer can increase the risk of developing a different type of cancer in the future. Your doctors will want to keep an eye out for signs of these secondary cancers. 3. Long-term side effects of treatment: Cancer treatments can sometimes have long-term or late-onset side effects. Regular check-ups can help catch and manage these side effects early. 4. General health maintenance: It's important for cancer survivors to take an active role in their overall health. Regular follow-ups can help you stay on top of any other health issues that may arise. 5. Emotional support: Living through a cancer diagnosis and treatment can have significant psychological impacts. Your doctors can provide resources and referrals to help manage any anxiety, depression, or other emotional issues you may be experiencing.   So, in summary, regular follow-up visits are a critical part of post-cancer care. Of course, the frequency and nature of these visits will vary depending on your specific circumstances, such as the type and stage of cancer you had, the treatments you received, your overall health, and other factors. You should discuss with your doctor what kind of follow-up plan is best for you. | Comparable | 1 | 1 | Not applicable |
| 106. What are the side effects of chemotherapy for head and neck cancer? | Chemotherapy is an aggressive form of treatment that can have a variety of side effects. The side effects vary based on the type of chemotherapy, the overall health of the patient, and the stage of the cancer. Common side effects of chemotherapy for head and neck cancer may include:   1. Mouth and Throat Problems: Patients might experience mouth sores, difficulty swallowing, dry mouth, changes in taste or loss of taste, and voice changes. These side effects can lead to malnutrition and weight loss. 2. Nausea and Vomiting: This is one of the most common side effects. It can usually be managed with medication. 3. Hair Loss: Hair loss is common with certain chemotherapy drugs. This can occur not just on the scalp, but also on other parts of the body. 4. Fatigue: Patients often feel extremely tired during chemotherapy. 5. Low Blood Cell Counts: Chemotherapy can cause a decrease in the number of blood cells, leading to anemia (low red blood cell count), neutropenia (low white blood cell count), and thrombocytopenia (low platelet count). This can increase the risk of infections, fatigue, and bleeding. 6. Neuropathy: Some chemotherapy drugs can cause nerve damage, leading to symptoms like numbness, tingling, or even pain in the hands and feet. 7. Skin and Nail Changes: Skin may become dry, itchy, or rashy, and nails may become darkened or cracked. 8. Hearing Loss: Certain chemotherapy drugs may cause hearing loss or tinnitus (ringing in the ears). 9. Changes in Appetite and Weight: Patients may experience a loss of appetite or changes in taste, which may contribute to weight loss. 10. Mental and Emotional Effects: Patients may experience mood changes, anxiety, or depression. 11. Fertility Issues: Chemotherapy can affect fertility in men and women.   While these side effects can be challenging, there are many ways to manage them, and many of them go away after treatment is completed. If you or someone you know is undergoing chemotherapy, it's crucial to communicate with the healthcare team about any side effects that are being experienced. They can provide strategies or medications to help manage these side effects. | Comparable | 1 | 1 | Not applicable |
| 107. What are the side effects of immunotherapy for head and neck cancer? | Immunotherapy for head and neck cancer, like for any cancer, involves stimulating the body's immune system to fight the disease. It is generally better tolerated than traditional treatments like chemotherapy and radiation. However, it does have potential side effects that may vary based on the type of immunotherapy used.   1. Infection Risk: Since immunotherapy stimulates the immune system, there can be a risk of getting infections more easily. 2. Allergic Reactions: These might include skin rash, itchiness, fever, chills, nausea, or breathing problems. 3. Flu-Like Symptoms: Fatigue, fever, chills, weakness, nausea, vomiting, dizziness, muscle or joint aches can occur. 4. Organ Inflammation: Organs like the liver, lungs, heart, and intestines can be inflamed, leading to symptoms like yellowing of the skin (jaundice), shortness of breath, diarrhea, or abdominal pain. 5. Autoimmune Reactions: In some cases, the immune system may start attacking healthy cells, causing a range of symptoms, such as skin problems, hormonal imbalances, and issues with various organs. 6. Endocrine Changes: Immunotherapy can sometimes cause changes in the body's endocrine system, leading to issues like thyroid disorders or adrenal insufficiency. 7. Neurological problems: Some patients may experience headaches, confusion, changes in mood or behavior, seizure, or problems with balance or coordination. 8. Pneumonitis: This is an inflammation of the lungs that can cause cough, chest pain, or shortness | Comparable | 1 | 1 | Not applicable |
| 108. What are the side effects of targeted drug therapy for head and neck cancer? | Targeted drug therapy works differently from standard chemotherapy. It targets specific genes, proteins, or the tissue environment that contribute to cancer growth and survival. While targeted therapies may have fewer side effects than traditional chemotherapy, they can still cause side effects, including:   1. Skin problems: This is one of the most common side effects. It can include dry skin, rash, or other changes in skin color. Some people might experience hair color changes, too. 2. High blood pressure: Some targeted therapies can cause high blood pressure (hypertension). Regular monitoring of blood pressure is essential during treatment. 3. Bleeding, poor wound healing: These therapies can affect blood clotting and wound healing, which could lead to bleeding. 4. Fatigue: Feeling very tired is a common side effect of many cancer treatments, including targeted therapy. 5. Gastrointestinal issues: These may include diarrhea, nausea, mouth sores, loss of appetite, or other digestive problems. 6. Problems with liver function: Some targeted drugs can cause changes in liver function, which can be detected through blood tests. 7. Heart problems: Some targeted therapies might affect the heart, which is why the heart function should be tested before starting such therapy. 8. Flu-like symptoms: Some patients may experience symptoms such as fever, chills, headache, muscle and joint aches, or shortness of breath.   These are potential side effects, but not all patients will experience them. The side effects depend on the specific drug used, the dosage, the overall health of the patient, and how the patient's body reacts to the therapy. Always consult with your doctor about potential side effects and management strategies before starting any new treatment. | Comparable | 1 | 1 | Not applicable |
| 109. What are the side effects of radiotherapy for head and neck cancer? | Radiotherapy, or radiation therapy, is a common treatment for head and neck cancer. However, it can cause several side effects due to the sensitivity of the areas being treated. Not everyone experiences the same side effects, and the severity can vary widely from person to person. Here are some common side effects:   1. Mouth and Throat Changes: Radiotherapy can cause sores in the mouth or throat, dry mouth, difficulty swallowing, changes in taste or loss of taste, and speech difficulties. These problems can make it difficult to eat and drink, leading to weight loss and malnutrition. 2. Skin Problems: Skin in the treatment area may become red, dry, or itchy, similar to a sunburn. This condition is known as radiation dermatitis. 3. Fatigue: Many people experience fatigue during radiotherapy, which may continue for some time after treatment is completed. 4. Hair Loss: Hair loss may occur in the area being treated. 5. Dental Problems: Radiation can cause dental issues such as tooth decay, especially when the radiation is directed towards the mouth or throat. 6. Hearing Loss: This is a potential side effect if the treatment area includes the ear. 7. Neck Stiffness: Radiotherapy can lead to stiffness or discomfort in the neck due to damage to the soft tissues. 8. Lymphedema: Some patients develop lymphedema, or swelling caused by a blockage in the lymphatic system, in the neck or face. 9. Long-term effects: Over time, radiotherapy can lead to problems such as fibrosis (the thickening or scarring of tissue), damage to the salivary glands (which can lead to dry mouth), thyroid changes, or secondary cancers.   Please note that most side effects are manageable with the help of your medical team. For example, medications can help manage mouth sores and dry mouth, and dietitians can provide guidance on maintaining nutrition | Comparable | 1 | 1 | Not applicable |
| 110. What methods of voice restoration are available after total laryngectomy? | Total laryngectomy, a surgical procedure that involves the removal of the entire larynx or voice box, can leave a person without the natural ability to speak. However, several methods can be used to restore speech after this procedure. These methods generally fall into three categories: esophageal speech, electrolarynx, and tracheoesophageal puncture.   1. Esophageal Speech: This is a method of speech that is accomplished by swallowing air, trapping it in the esophagus, and then releasing it to create sound. This air can then be modulated into speech by the articulatory movements of the lips, tongue, and palate. It often requires significant training and practice to become proficient and the voice may be quieter and less fluent than natural speech. 2. Electrolarynx: This is a battery-operated device that produces sound which is then formed into speech by the movements of the lips, tongue, and palate. The user places the device against the skin of the neck or cheek, where it produces vibrations that are then modulated into speech. While the electrolarynx allows for immediate voice restoration after surgery, the resulting voice often has a mechanical quality. 3. Tracheoesophageal Puncture (TEP): This is a surgical procedure that creates a small hole, or fistula, between the trachea and the esophagus. A small one-way valve is inserted into this hole. When the tracheostoma (the opening at the base of the neck from the trachea to the outside) is covered, air from the lungs is directed into the esophagus and out through the mouth, allowing for speech. With this method, the pitch and volume of the voice can be more easily controlled, resulting in a voice that is often more natural-sounding than with other methods. 4. Voice Prosthetics: Voice prostheses can be used in combination with the TEP procedure. These devices are typically small, one-way valves that prevent food and liquid from entering the trachea but allow air to pass from the trachea into the esophagus.   Speech and language therapists often work closely with individuals who have undergone total laryngectomies to determine the most suitable method of voice restoration and to provide training and support. The choice of method often depends on the person's individual circumstances, including their overall health, the nature of their cancer and its treatment, and their personal preferences. | Comparable | 1 | 1 | Not applicable |
| 111. How can i gain weight after my treatment for head and neck cancer ends? | After head and neck cancer treatments such as surgery, radiation, or chemotherapy, it's common for patients to have difficulties with eating and maintaining their weight. This may be due to side effects such as pain, loss of appetite, dry mouth, taste changes, or difficulty swallowing. Here are some general strategies to help you gain weight and improve your nutrition after treatment:   1. Consult a Dietitian: A registered dietitian can provide personalized dietary guidance based on your specific needs and preferences. They can suggest specific foods and meal plans, as well as strategies for managing any ongoing side effects from your treatment. 2. Eat Protein-Rich Foods: Include more protein in your diet. This is especially crucial after cancer treatment as protein helps to repair cells and tissues. Foods like lean meat, fish, poultry, eggs, dairy products, legumes, and nuts are good sources of protein. 3. Eat Small, Frequent Meals: Instead of three large meals a day, aim for five or six smaller meals. This can be helpful if you're dealing with appetite loss or if you feel full quickly. It's easier on the stomach and ensures you have a steady intake of calories throughout the day. 4. Nutrient-Dense Foods: Go for foods high in calories and nutrients. You can enhance your meals by adding ingredients like oils, butter, nuts, seeds, and avocado. 5. Stay Hydrated: Dry mouth and throat are common side effects of head and neck cancer treatments. Sip on water throughout the day, and consider other sources of fluids like smoothies, milk, or soup. 6. Use Nutritional Supplements: If you're struggling to eat enough food, your dietitian might recommend a nutritional supplement. These can be in the form of shakes, powders, or bars. 7. Practice Good Oral Hygiene: If mouth sores or a dry mouth make it difficult to eat, maintaining good oral hygiene can help. Rinse your mouth before meals, and brush your teeth regularly with a soft toothbrush. 8. Consider Physical Activity: Depending on your recovery status and doctor's advice, light physical activity such as walking can help stimulate appetite and improve your overall health. 9. Mind Your Medications: Some medications might cause side effects that can interfere with your eating or digestion. If this is the case, discuss these effects with your healthcare team. 10. Support System: It's important to have a strong support system. Your loved ones can help you shop for groceries, prepare meals, and provide emotional support.   It's very important to remember that everyone's journey is different. What works for one person might not work for another. Regular check-ups with your healthcare team will allow them to monitor your weight and nutrition status and adjust your care plan as necessary. Please talk to your healthcare provider before making any significant changes to your diet or lifestyle. | Comparable | 1 | 1 | Not applicable |
| 112. How long does it typically take for swelling to resolve after radiotherapy for head and neck cancer? | The swelling or edema after head and neck radiotherapy can take some time to resolve, and it can vary greatly between individuals. Factors that influence the length of this process include the individual's overall health, the specific location and intensity of the radiotherapy, and their body's response to the treatment.  Generally speaking, mild swelling can begin to reduce within a few weeks to a couple of months after the completion of radiotherapy. However, more significant swelling, especially if it's associated with fibrosis (a form of scar tissue), can take several months or longer to resolve. In some cases, a degree of swelling may persist, and additional interventions (like physiotherapy, medication, or even further surgery) might be necessary.  It's important to note that each patient's experience with radiotherapy and its aftereffects is unique, and what holds true for one person might not hold true for another. Therefore, patients should regularly consult with their healthcare provider to understand what to expect in their specific case and to monitor their recovery progress. Any significant or lasting changes in swelling should be promptly discussed with their healthcare provider.  Keep in mind that while radiotherapy is an effective treatment for many head and neck cancers, it does come with potential side effects, including swelling, pain, changes in skin appearance, dry mouth, difficulty swallowing, and changes in taste, among others. Many of these can be managed with the appropriate supportive care, and they typically improve over time. | Comparable | 2 | 2 | Not applicable |
| 113. What are the late side effects of cobalt radiation for nasopharyngeal cancer? | Cobalt radiation, often used in radiation therapy for nasopharyngeal cancer, is generally effective in targeting and killing cancer cells. However, it can cause several late side effects, which are issues that may persist or begin months to years after the completion of treatment. These side effects are due to damage to healthy tissue in the radiation field.  Here are some potential late side effects:   1. Dry mouth (xerostomia): Cobalt radiation can damage the salivary glands, which may lead to chronic dry mouth. This can affect taste, swallowing, and oral health. 2. Dental problems: Radiation can weaken the jawbone and damage teeth. It can also lead to a higher risk of cavities and gum disease due to dry mouth. 3. Difficulty swallowing (dysphagia): Over time, the radiation can cause the throat muscles to stiffen and scar, making swallowing difficult. This can affect nutrition and hydration. 4. Hearing loss: Radiation can damage the structures of the ear, leading to hearing loss or tinnitus (ringing in the ears). 5. Speech problems: If the radiation affects the muscles involved in speech, it could lead to voice changes or speech difficulties. 6. Nasal congestion: Chronic nasal congestion or nosebleeds can occur if the radiation damages the nasal passages. 7. Radiation fibrosis syndrome: This is a late effect that includes multiple symptoms like fibrosis (hardening or scarring) of muscles, skin, and internal organs; joint stiffness; difficulty swallowing; and loss of muscle strength. 8. Cognitive problems: Though less common, some people may experience memory problems, difficulty concentrating, or other cognitive changes. 9. Radiation-induced malignancies: A very long-term risk of radiation therapy is the potential for a secondary cancer to develop, though this risk is generally low.   Managing these side effects often involves a multidisciplinary approach with various specialists. It's also important to note that not everyone will experience all of these side effects; it varies based on individual factors such as overall health, the exact location of the radiation, the dosage, and the treatment duration. Always discuss the risks and benefits of treatment options with your healthcare provider. | Comparable | 1 | 1 | Not applicable |
| 114. What is the survival expectancy for stage 1 glottic larynx carcinoma? | As of my last update in September 2021, the 5-year relative survival rate for localized (including stage 1) laryngeal cancer, which would include glottic larynx carcinoma, is around 77%. However, survival rates can vary based on numerous factors, such as the patient's overall health, age, response to treatment, and others. Also, medical advancements after my last update may have influenced survival rates. It's essential to consult with a healthcare professional for the most accurate and up-to-date information. | Comparable | 3 | 3 | Not applicable |
| 115. How common is lymphedema after treatment for head and neck cancer? | Lymphedema, a condition of localized fluid retention and tissue swelling, is a possible complication following treatment for head and neck cancer, especially after surgery or radiation therapy that impacts the lymph nodes.  While I can't provide specific prevalence rates after 2021, at the time of my last training cut-off in September 2021, the prevalence of lymphedema following head and neck cancer treatment was estimated to be between 12% and 54%, depending on the study and the population examined. The wide range of estimates is due to variations in factors like treatment modalities, study design, methods of diagnosing lymphedema, and follow-up duration.  Given the lack of standardized diagnostic criteria and underdiagnosis, the actual prevalence could be higher. It's also important to note that lymphedema can develop months or even years after treatment, so long-term follow-up is necessary for a thorough understanding of this complication.  Remember, individual risk can vary significantly depending on the specifics of the cancer and treatment. Therefore, it's crucial to discuss the risk of lymphedema with the healthcare provider in charge of the treatment plan. | Comparable | 2 | 1 | 1 |
| 116. Will the scar on my neck gradually disappear following the neck dissection operation? | The healing and fading of scars can vary from person to person and depend on various factors, such as the individual's genetics, the type and extent of the surgery, and the care taken during the healing process.  After a neck dissection operation, it's common to have a scar in the neck area. Initially, the scar may appear red, raised, or slightly prominent. Over time, scars generally go through a maturation process and may become less noticeable. They can gradually fade and flatten, although they might not completely disappear. The extent of scar improvement varies for each person.  To help minimize the appearance of the scar and aid in the healing process, you can follow your healthcare provider's instructions, such as keeping the incision site clean, avoiding excessive sun exposure, and using scar management techniques like silicone sheets or gels, which may help with scar healing.  It's always best to consult with your healthcare provider or surgeon for personalized advice regarding your specific situation and the expected healing process of your scar after a neck dissection operation. They can provide you with more accurate information based on your medical history and the details of your surgery. | Comparable | 1 | 1 | Not applicable |
| 117. How long does it typically take to resume oral feeding after a total laryngectomy? | The length of time it takes for a person to resume oral feeding after a total laryngectomy can vary greatly, depending on various factors such as the individual's health condition, the extent of the surgery, and the success of post-operative care including rehabilitation.  Generally speaking, oral intake is not allowed immediately after surgery. A feeding tube is usually placed during surgery to provide nutrition while the surgical site heals. This is often a temporary arrangement, but it can become permanent if swallowing function is not sufficiently recovered.  Patients typically start by swallowing small amounts of clear liquids once their doctor has determined that it's safe to do so. This may happen several days to a week or more after surgery. From there, they will slowly progress to more solid foods as tolerated.  Swallowing rehabilitation is an important part of recovery following a total laryngectomy. This typically involves working with a speech-language pathologist, who can provide exercises and strategies to help improve swallowing function and reduce the risk of complications such as aspiration.  Overall, the process of resuming oral feeding can take from several weeks to several months, and in some cases, might not be fully possible. It's important to discuss this with the surgical team and to follow their advice regarding diet and swallowing therapy. | Comparable | 1 | 1 | Not applicable |
| 118. What is the risk of hearing loss following radiotherapy? | Radiation therapy, also known as radiotherapy, can potentially cause hearing loss. This is particularly true if the radiation treatment is directed near the ear, such as for tumors in the brain, neck, or head. The extent of hearing loss can vary, and it may occur immediately after treatment or develop gradually over several months or even years.  Radiotherapy can cause damage to various structures in the ear, leading to hearing loss:   1. Inner ear: The cochlea, located in the inner ear, is responsible for transmitting sound signals to the brain. Radiation can damage the cochlea, leading to sensorineural hearing loss. 2. Middle ear: Radiation can cause inflammation and accumulation of fluid in the middle ear, a condition called otitis media. This can result in conductive hearing loss. 3. External ear: Radiation can lead to skin inflammation and narrowing of the ear canal, which may also contribute to hearing loss. 4. Auditory nerves: High doses of radiation can damage the nerves that transmit auditory signals from the ear to the brain, leading to neural hearing loss.   The risk of hearing loss from radiotherapy can be influenced by several factors including the total dose of radiation, the treatment technique, the volume of the ear exposed to radiation, and individual patient characteristics. Concurrent chemotherapy, especially with certain ototoxic drugs, can also increase the risk of hearing loss.  It's also important to note that the onset of hearing loss may be gradual and may not become apparent until several years after the treatment. Therefore, regular hearing assessments are often recommended for patients who have received radiotherapy in the head or neck area.  Please discuss any concerns you might have about potential side effects of radiotherapy, including hearing loss, with your healthcare provider. They can provide more specific information based on the details of the treatment plan and your personal health history. | Radiation therapy, also known as radiotherapy, is a type of cancer treatment that uses high doses of radiation to kill cancer cells and shrink tumors. However, like many treatments, radiotherapy does have potential side effects. These can vary widely, depending on the location of the treatment, the type of radiation used, the dosage, the patient's overall health, and other factors.  When radiotherapy is used to treat tumors in areas around the ear, such as head and neck cancers, there is a risk of hearing loss. This is because the radiation can damage the structures within the ear that are responsible for hearing. The cochlea, the auditory nerve, and other related structures can all be impacted by radiation. In some cases, this damage may be temporary, but in others, it could be permanent.  The actual risk of hearing loss varies from person to person and depends on many factors, such as:   1. The location of the cancer: If the tumor is close to the ear, the risk is higher. 2. The radiation dose: Higher doses of radiation pose a higher risk of hearing loss. 3. The type of radiation therapy: Some types of radiotherapy may pose a higher risk of hearing loss than others. 4. Individual factors: The person's age, overall health, and pre-existing hearing issues can also affect the risk.   According to some studies, the prevalence of radiation-induced sensorineural hearing loss is quite high, especially in patients treated for head and neck cancers. For instance, a study published in "The Laryngoscope" in 2018 found that almost 50% of patients experienced some degree of hearing loss after radiation therapy for head and neck cancer.  However, it's important to understand that these risks must be weighed against the potential benefits of treating the cancer. In many cases, the benefit of controlling or eliminating the cancer may outweigh the risk of potential side effects like hearing loss.  Finally, strategies such as "radiation shielding" (protecting certain areas from radiation) or the use of certain drugs might help to mitigate this risk, but these decisions should be made in close consultation with the treating physician.  As of my knowledge cutoff in September 2021, the science behind preventing and treating radiation-induced hearing loss is still evolving. It's possible that there have been advancements in this area since then. Always consult with your healthcare provider for the most accurate and up-to-date information. | 1,2 | 1,1 | 1,1 |
| 119. I experience sweating in my face during eating after parotidectomy. What causes this? | What you're experiencing is called Frey's syndrome, also known as gustatory sweating. This condition often occurs as a side effect after surgery involving the parotid glands, such as a parotidectomy. The parotid glands are a pair of salivary glands located just in front of your ears, and they are responsible for producing saliva to aid in the digestion of food.  During a parotidectomy, nerve fibers can sometimes be severed, which can then mistakenly reattach themselves to sweat glands instead of the salivary glands during the healing process. The end result is that certain triggers, like eating, that would normally stimulate saliva production, can instead stimulate sweat production. This can result in sweating and flushing in the areas around your cheeks and ears.  While Frey's syndrome isn't generally a serious condition, it can sometimes be uncomfortable or embarrassing. Treatments are available, and can include topical medications, Botox injections, or in more severe cases, surgical intervention. It's recommended that you speak to your doctor if you're experiencing symptoms of Frey's syndrome, they will be able to provide you with more specific advice and treatment options based on your individual circumstances. | Comparable | 1 | 1 | Not applicable |
| 120. What are the intraoperative risks associated with neck dissection? | Neck dissection is a significant surgical procedure used primarily to treat cancers of the head and neck, including those originating in the oral cavity, throat, larynx, and thyroid. Like any surgical procedure, neck dissection carries potential risks, and the intraoperative risks, or those that can occur during the surgery itself, include:   1. Bleeding: As with any surgical procedure, there's a risk of significant blood loss. The neck is a region with many blood vessels, and careful surgical technique is required to minimize this risk. 2. Injury to the major blood vessels: The neck contains major arteries and veins, such as the carotid artery and the jugular vein. Injury to these blood vessels can lead to serious complications, including stroke. 3. Nerve injury: The neck houses numerous important nerves such as the spinal accessory nerve, vagus nerve, hypoglossal nerve, and the cervical plexus, which can potentially be injured during surgery. This can lead to complications like shoulder dysfunction, difficulty swallowing, changes in voice, or even paralysis of certain muscles. 4. Infection: Although antibiotics are typically given before the surgery to reduce the risk, there is always a potential for wound infection, which can complicate recovery. 5. Anesthesia risks: These include adverse reactions to anesthesia, aspiration pneumonia, and in rare cases, death. Prior to surgery, the anesthesiologist will usually perform an evaluation to assess these risks. 6. Inadvertent injury to other structures: This can include injury to the parathyroid glands (which can affect calcium regulation in the body) or the trachea or esophagus. 7. Chyle leak: This is a rare complication where lymphatic fluid leaks from damaged lymphatic channels. This condition may require further intervention or prolonged hospital stay.   These risks are typically minimized by careful preoperative planning, use of skilled and experienced surgical teams, and appropriate intraoperative techniques and monitoring. The specific risks can also vary depending on the exact type and extent of the neck dissection, the nature of the underlying disease being treated, and the patient's overall health. | Comparable | 1 | 1 | Not applicable |
| 121. Is there a risk of hoarseness after thyroidectomy? | Yes, there is a risk of hoarseness after thyroidectomy, which is the surgical removal of all or part of the thyroid gland.  The thyroid gland is located in the neck, very close to the laryngeal nerves which control the vocal cords. Therefore, during thyroidectomy, these nerves can potentially be injured, leading to voice changes such as hoarseness, voice fatigue, or even voice loss.  The risk of permanent voice change after thyroidectomy is relatively low (1-2%), but temporary voice changes are more common.  The superior laryngeal nerve, if injured, can cause a change in the pitch of the voice and difficulty in hitting high notes for singers. The recurrent laryngeal nerve, on the other hand, if damaged, can cause a breathy, weak voice or even voice loss if both sides are affected.  There are techniques and technologies, such as nerve monitoring, that surgeons use to help minimize the risk of nerve damage. Also, the risk varies depending on the reason for the surgery, the person's overall health, the skill and experience of the surgeon, and the specific surgical procedure used.  After surgery, if a patient experiences voice changes, they may be referred to a speech-language pathologist for voice therapy. In some cases, additional treatments or surgeries may be needed to improve the voice.  It's always important to discuss these risks with your surgeon before the procedure to understand the potential outcomes and how they can be managed. | Comparable | 1 | 1 | Not applicable |
| 122. I had a parotidectomy operation for cancer and I feel severe pain in front of my ear during the first few bites of food. What could be the cause? | The symptoms you're experiencing may be due to a condition known as Frey's syndrome. This condition occurs when the parotid gland, which is responsible for producing saliva, is damaged, typically during a parotidectomy. This can lead to abnormal connections between the nerve fibers that were originally serving the salivary glands and the sweat glands in the skin.  Frey's syndrome is characterized by sweating, flushing, and warmth over the area of the face where the surgery occurred, most commonly during eating, thinking about food, or smelling food. The first bite phenomenon, characterized by pain in the parotid region during the first few bites of a meal, is related but distinct from Frey's syndrome.  First bite syndrome is another potential cause of your symptoms. This condition typically develops after surgery in the upper neck or parotid gland and is characterized by severe pain in the area of the surgery when the individual first begins to eat. The pain usually lessens with each subsequent bite.  These are just possibilities, and it's important that you consult with your surgeon or another healthcare provider. Pain could be due to various other reasons such as infection, nerve damage, or other post-surgical complications. It's crucial to get a proper examination and diagnosis for your condition. Please seek medical attention as soon as possible. | I'm an AI and while I can provide information based on my training, I'm not a doctor and cannot diagnose or provide treatment advice for specific medical conditions. However, I can share that the symptom you're describing is often associated with a condition called Frey's syndrome.  Frey's syndrome, also known as auriculotemporal syndrome, is a condition that can occur after a parotidectomy (the surgical removal of the parotid gland). Symptoms typically include flushing and sweating on the side of the face where the surgery occurred, often triggered by eating, thinking about food, or smelling food. The syndrome happens because some of the nerves that used to go to the parotid gland (which produces saliva) may regrow and connect to sweat glands in the skin. So when you start eating and the body thinks it's time to produce saliva, it may instead stimulate those sweat glands and also cause flushing or pain.  In some cases, the severe pain you're experiencing could also be due to issues with the healing process or nerve damage from the surgery, such as in cases of trigeminal neuralgia or post-surgical neuralgia.  It's extremely important that you consult with your doctor or a healthcare professional about these symptoms, as they're the ones who can provide an accurate diagnosis and treatment plan for your specific situation. Please don't delay seeking medical advice based on information you read here. | 3,4 | 3,3 | 3,3 |
| 123. What are the postoperative risks of transoral robotic surgery? | Transoral Robotic Surgery (TORS) is a minimally invasive procedure that uses a surgical robot to remove benign or malignant lesions in the mouth and throat. Like all surgeries, TORS carries some risks. However, it's important to note that every patient is unique, and these risks can vary depending on the individual's overall health, the nature of the tumor, and the specifics of the surgery.  Here are some potential postoperative risks associated with TORS:   1. Bleeding: As with any surgical procedure, there's a risk of postoperative bleeding. In rare cases, severe bleeding may require additional interventions or surgery. 2. Infection: Infections can occur after any surgery. Antibiotics are often administered to prevent this. 3. Difficulty swallowing (dysphagia): This can occur if the surgery affects the muscles used for swallowing. It's usually temporary but can be long-lasting in some cases. 4. Speech difficulties: Depending on the exact area operated on, some patients may experience temporary or permanent changes to their speech. 5. Aspiration: Aspiration, or inhaling food or liquid into the airways, can occur if the surgery affects the muscles used for swallowing. This can potentially lead to pneumonia. 6. Changes in taste or loss of taste: This is usually temporary but can be permanent in some cases. 7. Nerve injury: There's a small risk of nerve damage, which could affect sensation and function in the mouth and throat. 8. Need for tracheostomy or gastrostomy: In some cases, a temporary or permanent tracheostomy (an opening created at the front of the neck so a tube can be inserted into the windpipe to aid breathing) or gastrostomy (a feeding tube) may be required after surgery. 9. Psychological impact: Some patients may struggle with the psychological impact of a cancer diagnosis and treatment, which can include issues such as depression and anxiety. 10. Impact on quality of life: This can be influenced by many factors, including physical changes after surgery, the need for additional treatments, and the stress and anxiety of dealing with a serious illness.   These risks need to be weighed against the potential benefits of the surgery. It's crucial to have a thorough discussion with your healthcare team to understand these risks and benefits and make the best decision for you. | Comparable | 1 | 1 | Not applicable |
| 124. What are the options for reconstruction after lip surgery? | After lip surgery, particularly if it's for the removal of a lip cancer or a trauma injury, the ultimate goal is to preserve or restore both function (such as speech, eating, and drinking) and aesthetic appearance. The following are some of the reconstruction options:   1. Local Flaps: The use of local flaps involves moving adjacent healthy tissue into the area where tissue has been lost. These flaps are designed to match the color and texture of the lips. The rotation or advancement flaps are commonly used techniques. 2. Regional Flaps: When more tissue is needed for larger defects, regional flaps may be used. This involves taking tissue from a nearby area (such as the cheek or neck) and rotating it into the defect. 3. Free Flaps: This procedure uses tissue from a distant site of the body (like the forearm, thigh or lower leg). This tissue, along with its blood vessels, is transferred to the lip and the blood vessels are connected using microsurgery. This is typically done for very large defects. 4. Lip Advancement: In cases of small defects, it may be possible to simply advance the remaining lip tissue to cover the area where tissue has been removed. This is often referred to as primary closure. 5. Full or Partial Thickness Skin Grafts: Skin grafts can be used when the defect is superficial or not too deep. These grafts are taken from another part of the body. 6. Abbe or Estlander Flap: These are types of local flap procedures typically used when the defect involves the border of the lip. The Abbe flap is a full-thickness flap that is taken from the lower lip to reconstruct the upper lip, or vice versa. The Estlander flap is similar but typically used when the corner of the mouth is involved. 7. Lip Implants: For cosmetic purposes, lip implants can be used to restore volume and shape. This may be done after healing has occurred, depending on the circumstances. 8. Composite Grafts: These are grafts taken from other parts of the body, which contain multiple tissue types (skin and cartilage or skin and mucosa for example). These are typically used for smaller defects. 9. Nasolabial Flaps: These are flaps of skin and underlying tissue that are rotated into the defect from the nasolabial fold (the crease running from the side of the nose to the corner of the mouth).   Choosing the best option depends on a variety of factors, including the size and location of the defect, the patient's overall health, and the surgeon's experience. Your healthcare provider will be able to provide advice tailored to your specific circumstances. Please note that all these procedures carry risks, including infection, scarring, and changes in sensation. | Comparable | 1 | 1 | Not applicable |
| 125. I have been treated with chemoradiotherapy for nasopharynx carcinoma and have a recurrent tumor in my neck. What is the best treatment option? | I'm sorry to hear about your diagnosis. As an AI language model, I can provide some general information based on my training data up until September 2021. But, keep in mind that the decision should be made based on consultation with your healthcare provider, who has access to your detailed medical history and current status.  There are several treatment options available for recurrent nasopharyngeal carcinoma. The choice depends on several factors, including the site of recurrence, your general health, previous treatments, and your personal preferences.   1. Retreatment with Radiation: If your previous treatment with radiation was at a low dose, your doctor might consider a second course of radiation therapy. 2. Surgery: In some cases, surgery may be possible to remove the recurrent tumor, especially if it is localized to one area such as the neck. This could involve a neck dissection, which is a surgery to remove lymph nodes and surrounding tissue. 3. Chemotherapy: This may be used alone or in combination with other treatments, especially if the cancer has spread or if the patient is not a candidate for radiation or surgery. Several types of chemotherapy drugs can be used for nasopharyngeal carcinoma. 4. Immunotherapy: As of my training cut-off in 2021, immunotherapy has shown promise in treating recurrent nasopharyngeal carcinoma. It involves the use of drugs that help your immune system fight cancer. 5. Targeted Therapy: This involves the use of drugs or other substances that attack specific cancer cells with less harm to normal cells. Some targeted therapies focus on the functions inside cancer cells that allow them to live and grow. 6. Participation in Clinical Trials: Patients with recurrent nasopharyngeal carcinoma might also consider participating in clinical trials, which test new treatments that aren't yet available to the general public.   In 2021, the advancements in cancer treatments were significant, and it's likely more progress has been made since then. Thus, please consult with your healthcare provider to know the best options available to you. They'll be up-to-date on the latest research and treatment protocols. | Comparable | 1 | 1 | Not applicable |
| 126. What is the survival expectancy for stage 2 nasal cavity carcinoma? | Nasal cavity carcinoma is relatively rare, and survival rates often aren't broken down by stage. Instead, survival statistics are typically given for all stages of nasal and paranasal sinus cancers combined. According to the American Cancer Society, the 5-year relative survival rate for these cancers when combined is roughly 60% to 65%.  For more localized, early-stage cancers (like stage 2), the 5-year survival rate is typically higher. However, because this type of cancer is relatively rare and not as extensively studied as some other cancers, specific survival rates for each stage are less commonly available.  For the most accurate, up-to-date information, you should consult a medical professional or reliable medical resources, such as the website of the American Cancer Society or the National Cancer Institute. | Comparable | 2 | 2 | Not applicable |
| 127. Is it possible to speak with a tracheotomy cannula in place? | Yes, it is often possible for a person with a tracheotomy to speak, but it might be challenging and require adjustments. The main function of the larynx is to allow for speech by vibrating the vocal cords when air passes through them.  A tracheotomy is a surgical procedure that creates a direct airway through an incision in the trachea (the windpipe). It bypasses the mouth and larynx, which is where sound is generated. When a tracheotomy tube is in place, it may prevent air from passing over the vocal cords, which can affect the ability to speak.  However, there are special valves (called speaking valves or one-way valves like the Passy-Muir valve) that can be used to allow the individual to talk. These valves are placed on the tracheotomy tube and allow air to pass through the tube and over the vocal cords when the person exhales, enabling speech. The valve closes when the person inhales to make sure air goes through the tracheotomy tube and into the lungs.  Speech therapy may be required to help a person with a tracheotomy improve their speech. It can often be a slow process, but it is definitely possible with the right equipment, guidance, and patience.  It's also important to note that the ability to speak can depend on the individual's specific condition, the size and type of the tracheotomy tube, and other factors, so not everyone with a tracheotomy may be able to speak. As always, specific questions about an individual's ability to speak after a tracheotomy should be directed to a healthcare provider. | Yes, it is possible for a person with a tracheotomy to speak, but it may be challenging and require some adaptation.  A tracheotomy is a surgical procedure that involves making an incision in the front of the neck and creating an opening (a stoma) into the trachea (windpipe). A tube, known as a tracheostomy tube or cannula, is then inserted into the opening to maintain a clear airway for breathing.  Speech is produced when air passes from the lungs, through the vocal cords, and out of the mouth. However, a tracheostomy bypasses the vocal cords, meaning that air expelled from the lungs goes directly out of the tracheostomy tube, and doesn't pass over the vocal cords as it would normally. This makes it challenging for a person with a tracheostomy to speak.  However, there are ways to facilitate speech in someone with a tracheostomy:   1. Speaking Valve: A speaking valve can be attached to the tracheostomy tube. This is a one-way valve that lets air in when the person breathes in, but closes when they breathe out, forcing air to pass over the vocal cords and out through the mouth, facilitating speech. 2. Cuffless or Deflated Cuff Tracheostomy Tube: Tracheostomy tubes can be with or without a cuff. The cuff is a small balloon at the end of the tracheostomy tube that can be inflated to prevent air from escaping around the tube. For speech, a cuffless tube or a tube with a deflated cuff allows some air to flow past the tube, over the vocal cords, and out through the mouth. 3. Fenestrated Tracheostomy Tube: A fenestrated tracheostomy tube has small holes on the side of the tube which can allow air to flow over the vocal cords when the cuff is deflated. 4. Swallowing and Speech Therapy: Some patients may need therapy to learn how to manage their breath and coordinate speech and swallowing.   It's also important to note that the ability to speak after a tracheotomy can depend on several factors, including the reason for the tracheotomy, the patient's overall health, and whether there has been any damage to the vocal cords or nerves that control them. The healthcare team involved in the care of the person with the tracheotomy will be able to provide advice and support regarding communication options. | 2,1 | 2,1 | Not applicable |
| 128. How long does it take for taste buds to recover after treatments for head and neck cancer? | The length of time it takes for taste buds to recover after treatments like radiation therapy or chemotherapy for head and neck cancer can vary greatly depending on a variety of factors. Some of these factors include the type and intensity of the treatment, the specific location of the cancer, the individual's overall health, and their personal rate of recovery.  Radiation therapy often leads to a condition known as dysgeusia, a distortion of the sense of taste. This is usually a temporary side effect, but it can last for weeks or even months after treatment has ended. In some cases, a complete recovery of taste may take a year or longer.  Chemotherapy can also affect the sense of taste, typically causing foods to taste bland or metallic. This is usually temporary, with taste returning to normal within a few weeks to a few months after the end of treatment.  It's important to note that some people may not recover their sense of taste exactly as it was before treatment. Some people may experience long-term changes to their sense of taste. For example, they may find that certain foods they once enjoyed no longer taste good to them.  If you're experiencing prolonged or severe issues with taste following head and neck cancer treatments, you should consult with your healthcare provider. They may be able to recommend strategies to help you manage this side effect, such as dietary changes or medications. | Comparable | 1 | 1 | Not applicable |
| 129. How long after radiation therapy will I get my voice back? | Radiation therapy can cause a variety of side effects, including voice changes or voice loss. These side effects can be temporary or permanent depending on the individual case and the severity of the radiation treatment.  In cases of temporary voice loss or changes, patients may start to see improvements in their voice within a few weeks to a few months after the end of radiation treatment. For some, complete recovery can take 6 to 12 months, or even longer. There's also a chance that the voice may not return to how it was before treatment, especially if the radiation therapy is combined with surgery or chemotherapy.  In some cases, the voice changes might be permanent, especially if the vocal cords or surrounding nerves have been severely damaged by the radiation.  Recovery time can vary greatly among patients, as it depends on the individual's general health, the specific location of the cancer, the exact type and dose of radiation therapy used, and the degree of supportive care (such as speech therapy) available.  If you or someone you know is experiencing these symptoms, it's important to discuss them with your doctor or a specialist in order to get personalized medical advice. | Comparable | 1 | 1 | Not applicable |
| 130. What rehabilitation or support options are available for patients with head and neck cancers? | Head and neck cancers can significantly impact a person's physical, emotional, and social well-being. There are several types of rehabilitation and support options available for patients, which can be tailored to individual needs.   1. Physical and Occupational Therapy: These therapies can help manage physical challenges that come from head and neck cancers, such as difficulties with mobility, swallowing, speech, and performing daily activities. 2. Speech and Language Therapy: As head and neck cancers often affect speech and swallowing, speech and language therapists can work with patients to regain these abilities. They can provide exercises and techniques to improve swallowing and speech functions. 3. Nutrition Counseling: Given that eating and drinking can be difficult for people with head and neck cancers, a dietitian or nutritionist can provide guidance on managing nutritional needs, including recommending certain foods, liquid diets, or enteral feeding if necessary. 4. Psychological Counseling: The emotional toll of head and neck cancers can be significant. Psychological services, including individual, group, or family counseling, can provide emotional support and strategies for coping with the disease. 5. Social Services: Social workers can assist with various practical matters, such as coordinating care, helping with financial issues, and connecting patients with community resources. 6. Support Groups: These groups provide a community of individuals who are going through or have gone through similar experiences. They can offer a platform for sharing experiences, gaining advice, and receiving emotional support. 7. Palliative Care: This is specialized medical care for people with serious illnesses, focused on providing relief from the symptoms and stress of the disease. The goal is to improve quality of life for both the patient and the family. 8. Rehabilitation Medicine: Rehabilitation physicians, or physiatrists, can provide a comprehensive rehabilitation program that addresses the various physical and functional challenges resulting from the cancer or its treatment. 9. Dental Rehabilitation: Since head and neck cancers often involve surgery or radiation that can impact oral health, a dentist specializing in rehabilitation can be involved in the care team. | Comparable | 1 | 1 | Not applicable |
| ***CANCER PREVENTION*** |  |  |  |  |  |
| 131. How can I contract HPV? | Human papillomavirus (HPV) is the most common sexually transmitted infection (STI). HPV is so common that nearly all sexually active men and women get it at some point in their lives. There are many different types of HPV. Some types can cause health problems including genital warts and cancers.  HPV is transmitted through intimate skin-to-skin contact. This is usually sexual activity, including vaginal, anal, and oral sex, as well as close skin-to-skin contact during sexual activity. It can be passed on even when an infected person has no signs or symptoms.  You can also become infected with HPV by sharing sex toys that haven't been cleaned properly or covered with a new condom each time they're used. Although it's also possible to get HPV from kissing or close non-sexual contact, this is thought to be less common.  Furthermore, it's important to note that you can get HPV even if you only have one sexual partner. The virus can also take years to show symptoms, so it can be difficult to determine when or from whom you contracted it.  The HPV vaccine is a highly effective way to prevent many types of HPV infections, including those that cause most cases of genital warts and cervical cancer. Regular screening for cervical cancer and practicing safe sex can also help reduce your risk of HPV infection | Comparable | 1 | 1 | Not applicable |
| 132. How prevalent is tobacco use among survivors of head and neck cancer? | Cancer survivors, including those of head and neck cancers, are often advised to stop smoking in order to reduce the risk of cancer recurrence and improve their overall health. However, despite the known risks, some continue to smoke.  Research published up until my last training data (September 2021) has indicated that a significant proportion of head and neck cancer survivors continue to use tobacco even after diagnosis and treatment. The prevalence of continued smoking can vary widely depending on the study, but it's been reported in some research to be anywhere from 15-60%.  It's worth noting that continued tobacco use after a head and neck cancer diagnosis can lead to worse overall and cancer-specific survival, increased treatment-related toxicity, and a higher risk of developing a second primary cancer. | Comparable | 1 | 2 | 1 |
| 133. What evidence suggests that drinking alcohol can cause cancer? | There's significant evidence to suggest that alcohol consumption can increase the risk of certain types of cancer. This connection is backed by numerous observational and epidemiological studies. Here's an overview:   1. Biochemical evidence: Ethanol in alcohol is a toxic chemical that can damage cells, leading to cancer. When you drink alcohol, your body converts ethanol into a harmful chemical called acetaldehyde, which can damage DNA and proteins, leading to cellular mutation and cancer. 2. Epidemiological studies: Many large-scale epidemiological studies have found that alcohol increases the risk of certain types of cancer. This includes cancers of the mouth, throat, esophagus, larynx, liver, colon, rectum, and breast. The risk is especially high in people who use tobacco along with alcohol. 3. Dose-response relationship: There seems to be a dose-response relationship between alcohol and cancer. This means that the more alcohol a person drinks over time, the higher their risk of developing an alcohol-associated cancer. 4. Synergistic effect with smoking: Alcohol can also increase the risk of cancer by enhancing the effects of other carcinogens, like those in tobacco smoke. For instance, alcohol can help carcinogens from tobacco to penetrate the cells lining the mouth and throat more easily, increasing the risk of these types of cancer. 5. Hormone regulation: Alcohol can affect hormone levels in the body, particularly estrogen levels, which may explain why alcohol consumption has been linked with an increased risk of breast cancer in women. 6. Nutritional effects: Chronic heavy drinking can lead to deficiencies in vitamins and nutrients that protect against cancer, like folate, potentially increasing cancer risk.   It's important to note that while alcohol consumption does increase the risk of certain cancers, it does not mean that everyone who drinks alcohol will get cancer. The overall risk depends on many factors, including the amount of alcohol consumed, the duration of consumption, genetic factors, and other lifestyle factors. Moderation is generally advised for those who choose to consume alcohol. Always consult with a healthcare professional for personalized advice. | Comparable | 1 | 1 | Not applicable |
| 134. What can i do to prevent head and neck cancer? | Head and neck cancers can include a variety of different types such as cancers of the mouth, throat, nasal cavity, salivary glands, and neck. While you can't completely guarantee prevention, you can significantly lower your risk of developing these types of cancers by following these steps:   1. Avoid tobacco: Both smoking and smokeless tobacco significantly increase your risk of head and neck cancer. If you don't use tobacco, don't start. If you are currently a smoker, try to quit. There are many resources available to help you quit, including nicotine replacement therapy and support programs. 2. Limit alcohol consumption: Excessive alcohol consumption is another significant risk factor for head and neck cancers. If you choose to drink alcohol, do so in moderation. The American Cancer Society recommends no more than one drink a day for women and two for men. 3. Protect yourself from HPV: Some types of head and neck cancers are linked to the human papillomavirus (HPV). To protect yourself, consider getting vaccinated if you're in the appropriate age group. The HPV vaccine is most effective if given in the early teenage years, but it can still be beneficial in older age groups. In addition, use safe sexual practices, such as using condoms, which can reduce your risk of HPV infection. 4. Protect yourself from the sun: Some types of head and neck cancers, particularly those affecting the lips and skin, are related to sun exposure. Limit your time in the sun, especially during peak sunlight hours. Wear a wide-brimmed hat and use a broad-spectrum sunscreen with an SPF of at least 30. 5. Maintain a healthy diet: Consuming a diet that's rich in fruits and vegetables may help protect you from head and neck cancer. These foods are high in antioxidants, which can help protect cells from damage. 6. Regular exercise: Physical activity may help to prevent certain types of cancer, including head and neck cancer. 7. Regular check-ups: Regular dental check-ups can help detect oral cancers early. Your dentist might be able to identify suspicious-looking areas that might need further investigation. 8. Avoid exposure to certain hazardous substances: Regular or heavy exposure to certain substances, like asbestos or certain synthetic fibers, can increase your risk of certain types of head and neck cancers. Always use protective equipment and follow safety procedures if you work with such materials.   Remember, these steps can help reduce the risk, but they do not eliminate it completely. Always consult with healthcare professionals if you have concerns about specific symptoms or risks. It's also important to note that people who've never smoked or consumed alcohol can still develop head and neck cancer, and people who've had head and neck cancer can develop other types of cancer. | Comparable | 1 | 1 | Not applicable |
| 135. Does the daily intake of vitamin or mineral supplements decrease the risk of head and neck cancer? | As of my knowledge cutoff in September 2021, there is no solid scientific evidence to suggest that taking daily vitamin or mineral supplements directly decreases the risk of head and neck cancer. The effect of vitamins and minerals on cancer risk is a complex area of research with mixed findings.  The relationship between nutrition and cancer is multifaceted, and it can depend on many factors, including the specific type of nutrient, the dose, the timing of exposure, and individual differences in genetics and metabolism.  For example, while some studies suggest that a diet rich in fruits and vegetables—which are high in vitamins, minerals, and other phytochemicals—may lower the risk of certain types of cancer, it's not clear whether these benefits can be achieved through supplements. In fact, some studies suggest that certain supplements might increase cancer risk under specific circumstances.  Before starting any new supplement regimen, it's important to consult with a healthcare provider to discuss potential benefits, risks, and interactions with other medications or health conditions. A balanced diet, regular exercise, avoiding tobacco and alcohol, and other lifestyle factors are all important for cancer prevention.  It's crucial to note that research in this area is ongoing, and our understanding may have evolved after my last training data in September 2021. Therefore, for the most recent scientific information, please refer to the latest studies or consult with a healthcare professional. | Comparable | 1 | 1 | Not applicable |
| 136. Can an HPV vaccination decrease the risk of head and neck cancer? | Yes, vaccination against the human papillomavirus (HPV) can decrease the risk of developing several types of cancer, including those in the head and neck. This is because some strains of HPV (particularly HPV16 and HPV18) are known to cause cancers in these regions.  Oral HPV infection is a risk factor for oropharyngeal cancer, a type of head and neck cancer. Oropharyngeal cancers are often found in the throat, tonsils, and back of the tongue. It has been found that vaccination against HPV can lower the rates of these types of oral infections, which can subsequently reduce the risk of developing oropharyngeal cancers.  It's important to note that while the HPV vaccine can substantially lower the risk, it cannot eliminate it completely. Other factors, such as tobacco and alcohol use, can also contribute to the development of head and neck cancers. Therefore, it's still important to maintain regular health check-ups and engage in preventive health practices.  Lastly, it's also worth mentioning that the HPV vaccine is most effective when administered before an individual becomes sexually active and potentially exposed to the virus, which is why it is typically recommended for adolescents. However, adults who have not been previously vaccinated can also benefit from the vaccine. As of my knowledge cutoff in September 2021, the CDC recommends the HPV vaccine for everyone through the age of 26, and for some people aged 27 through 45 if they have not been previously vaccinated. Always consult with your healthcare provider for the most relevant and current recommendations. | Comparable | 1 | 1 | Not applicable |
| 137. Does an EBV infection increase the risk of head and neck cancer? | Epstein-Barr virus (EBV), a member of the herpesvirus family, is a common human pathogen that infects over 90% of the world's population. Infections are usually asymptomatic or cause a self-limiting illness such as infectious mononucleosis (also known as "mono" or the "kissing disease"). However, in some cases, EBV infection can lead to serious conditions including certain types of cancer.  Regarding head and neck cancers, EBV has been strongly associated with nasopharyngeal carcinoma (NPC), a type of cancer that starts in the nasopharynx, the upper part of the throat behind the nose and near the base of the skull. Studies have demonstrated that virtually all cases of undifferentiated NPC are associated with EBV.  While the connection between EBV and other types of head and neck cancers, like oral or laryngeal cancers, is not as strong, some research suggests a potential link. It's important to note that while EBV can contribute to the development of these cancers, it's typically one factor among many. Other common risk factors for head and neck cancers include tobacco and alcohol use, human papillomavirus (HPV) infection, and certain dietary factors.  In summary, while there is a well-established link between EBV infection and nasopharyngeal carcinoma, more research is needed to fully understand the role of EBV in other types of head and neck cancer. As always, anyone concerned about their risk of developing head and neck cancer should speak with their healthcare provider. | Comparable | 1 | 1 | Not applicable |
| 138. Should i avoid excessive sunlight exposure to prevent lip cancer? | Yes, you should. Both the skin and the lips are vulnerable to damage from the sun's ultraviolet (UV) radiation, which can increase the risk of developing skin cancer, including lip cancer. While lip cancer isn't as common as some other forms of skin cancer, it can still occur and is often linked to excessive sun exposure.  To protect yourself:   1. Limit your sun exposure: Try to avoid the sun when its rays are strongest, typically between 10 a.m. and 4 p.m. 2. Use lip balm with sun protection: There are lip balms available that offer SPF protection. Use one that is SPF 30 or higher, and reapply it every 2 hours and after eating or drinking. 3. Wear protective clothing: Broad-brimmed hats can help protect your face, including your lips, from the sun. 4. Never use tanning beds: Tanning beds emit UV radiation which can increase the risk of skin cancer. 5. Get regular check-ups: Regularly examine your lips for any changes such as the development of rough spots, sores that don't heal, or discolorations. See a doctor if you notice anything unusual.   Remember, the best way to prevent lip cancer is through a combination of sun protection measures, not just one. | Comparable | 1 | 1 | Not applicable |
| 139. Are there any herbal products that can help prevent head and neck cancer? | As of my last update in September 2021, there is no definitive scientific evidence to prove that any particular herb or herbal product can prevent head and neck cancer.  Cancer is a complex disease with multiple types and subtypes, each with its own set of risk factors. Several factors including genetic predisposition, lifestyle habits (like smoking and alcohol consumption), and environmental factors play a role in the development of head and neck cancer.  That being said, a healthy diet rich in a variety of fruits, vegetables, and whole grains, which include many naturally occurring bioactive compounds, can contribute to overall good health and may help in cancer prevention. Certain herbs and foods are rich in antioxidants and have anti-inflammatory properties which are generally associated with cancer prevention.  For example:   1. Turmeric: Curcumin, the active compound in turmeric, has been shown to have anti-inflammatory and antioxidant properties that may help prevent cancer. 2. Green Tea: Catechins in green tea may help prevent cancer by inhibiting the growth of cancer cells and reducing inflammation. 3. Cruciferous Vegetables: Broccoli, cabbage, and other cruciferous vegetables contain compounds like sulforaphane and indoles which have been studied for their potential cancer preventive properties. 4. Garlic: Some studies suggest that garlic consumption may have a protective effect against certain types of cancer, including possibly stomach and colorectal cancers.   However, it's important to note that these findings are generally based on population studies, animal models, or laboratory experiments, and the direct impact on humans is less clear. More clinical trials and studies are needed to conclusively understand their role in cancer prevention.  Moreover, these foods should not be seen as replacements for proven preventive measures like quitting smoking, limiting alcohol intake, protecting against HPV (a known cause of some head and neck cancers), and maintaining a healthy weight.  If you are interested in using herbs or other supplements for cancer prevention, it's essential to discuss it with your healthcare provider first. They can provide guidance on safe and effective use, and help ensure it doesn't interfere with any other medications or treatments you may be undergoing. Always seek advice from professionals when it comes to health matters. | Comparable | 1 | 1 | Not applicable |
| 140. I have been smoking for 25 years. Should i regularly get screened for a potential head and neck cancer? | Tobacco use, including smoking, is a significant risk factor for various types of cancer, including those of the head and neck, such as oral, laryngeal, and pharyngeal cancers. If you have a long history of smoking, discussing screening options with your healthcare provider would be a prudent step.  However, as of my knowledge cutoff in 2021, there is no universally agreed-upon regular screening procedure for head and neck cancers in asymptomatic individuals, like there is for some other cancers (such as mammography for breast cancer or colonoscopy for colorectal cancer). Screening recommendations often depend on specific risk factors, including age, family history, and lifestyle factors such as tobacco and alcohol use.  Nevertheless, it's important to have regular health check-ups, and if you have any symptoms suggestive of head and neck cancer, such as persistent sore throat, hoarseness, difficulty swallowing, ear pain, or unexplained weight loss, you should seek medical attention promptly.  The most important thing you can do for your health if you are a smoker is to quit smoking. Quitting smoking at any age can reduce the risk of developing cancer and other serious health conditions. It's never too late to quit. | Comparable | 1 | 1 | Not applicable |
| ***OTHER*** |  |  |  |  |  |
| 141. How common is HPV? | Human papillomavirus (HPV) is very common worldwide. As of my knowledge cutoff in September 2021, it's estimated that nearly all sexually active men and women will get at least one type of HPV at some point in their lives.  There are many different types of HPV. Some can cause health problems like genital warts and cancers. But there are vaccines that can stop these health problems from happening.  The prevalence can vary by region, age, and population, but it's generally widespread. A significant proportion of HPV infections are asymptomatic and resolve spontaneously, but persistent infection can lead to various types of cancer, including cervical, anal, penile, and oropharyngeal cancers.  In the United States, according to the Centers for Disease Control and Prevention (CDC), there are about 14 million new HPV infections each year, with about half of those in 15- to 24-year-olds. | Comparable | 1 | 1 | Not applicable |
| 142. Is it true that HPV-related head and neck cancers have better prognosis than those that are HPV-negative? | Yes, it is true that head and neck cancers associated with human papillomavirus (HPV) typically have a better prognosis than those that are not associated with the virus. HPV-positive oropharyngeal cancers, for example, respond better to treatment and have a higher overall survival rate than HPV-negative cancers of the same region.  This difference in prognosis is likely due to several factors. HPV-related tumors often have distinct molecular and genetic profiles compared to HPV-negative tumors, and these differences can make them more responsive to treatments such as radiation and chemotherapy. HPV-positive cancers are also more often diagnosed in younger patients, who are generally healthier and better able to withstand aggressive treatment.  However, while the prognosis for HPV-related head and neck cancers is generally better than for those that are HPV-negative, it's important to note that these are still serious conditions that require prompt diagnosis and treatment. As of my knowledge cutoff in September 2021, the exact reasons for this difference in prognosis are still being studied, and more research is needed to further improve outcomes for all patients with head and neck cancers. | Comparable | 1 | 1 | Not applicable |
| 143. What are the latest developments in laryngeal and hypopharyngeal cancer research and treatment? | I can share some of the potential advancements in laryngeal and hypopharyngeal cancer research and treatment that were being studied and developed up until 2021:   1. Immunotherapy: Immune checkpoint inhibitors, such as Pembrolizumab and Nivolumab, have been shown to be effective in treating several types of cancer, including head and neck cancers. These treatments work by boosting the patient's immune system and enabling it to better fight cancer cells. 2. Targeted therapy: Targeted therapies are drugs or other substances that can block the growth and spread of cancer by interfering with specific molecules that are involved in the growth, progression, and spread of cancer. 3. Gene therapy: Scientists have been studying the potential of modifying a person's genes to treat or prevent cancer. 4. Improved radiation therapy techniques: Such as Intensity-Modulated Radiation Therapy (IMRT) which uses computer-controlled x-ray accelerators to deliver precise radiation doses to a malignant tumor or specific areas within the tumor. 5. Improvement in robotic surgery: Techniques like Transoral Robotic Surgery (TORS) which offers the potential for surgeons to remove tumors through the mouth, avoiding the need for large, disfiguring incisions. 6. Better understanding of tumor biology: Researchers continue to learn more about the changes in cells that cause cancer, which may lead to new ways to help predict which cancers will respond best to certain treatments. 7. Biomarker Testing: Testing for biomarkers such as HPV status, PD-L1 expression, and more can help guide treatment decisions and prognosis. 8. Novel chemotherapy and biochemotherapy regimens: Improved combinations of chemotherapy drugs and new ways of giving these drugs are being explored to improve their effectiveness and reduce side effects. | Comparable | 1 | 1 | Not applicable |
| 144. Can I transmit an oral HPV infection to others? | Yes, oral HPV (human papillomavirus) can be transmitted to others. HPV is primarily spread through direct skin-to-skin contact, including oral sex and deep kissing, particularly when there are cuts or sores in the mouth or throat.  Oral HPV infection can potentially lead to oral and oropharyngeal (the middle part of the throat) cancers. It's important to note that while HPV infection is common, not everyone who gets an HPV infection will develop cancer. Other factors, such as one's immune response and lifestyle choices (like smoking or drinking alcohol), can influence the risk of developing cancer following HPV infection.  The HPV vaccine is a powerful tool in preventing the types of HPV most associated with cancer, including oral and oropharyngeal cancers. Regular dental checkups that include an oral cancer screening can also help detect signs of disease early. As always, be sure to consult with a healthcare provider for personal medical advice. | Comparable | 1 | 1 | Not applicable |
| 145. If I have HPV-related oropharyngeal cancer, is my spouse/partner also at risk of getting oropharyngeal cancer? | While it's true that the human papillomavirus (HPV) can be transmitted between partners, having HPV does not guarantee that either person will develop cancer. HPV is a common virus, and many people who have it never develop any health problems as a result.  HPV-related oropharyngeal cancer is associated with the transmission of HPV through oral sex, but not everyone who gets HPV will develop this kind of cancer. In fact, the body's immune system clears most HPV infections naturally within two years.  The risk of developing HPV-related oropharyngeal cancer depends on a variety of factors, including the person's immune response, genetic factors, the specific strain of HPV (not all strains are high-risk for cancer), and lifestyle factors such as smoking and heavy alcohol use.  HPV vaccines are available that can protect against the most dangerous types of the virus, and routine vaccination is recommended for both boys and girls.  If you have HPV or HPV-related oropharyngeal cancer, it's important to talk to your healthcare provider about the potential risks to your partner. They may recommend measures such as vaccination, regular health check-ups, or other precautions to help protect your partner's health.  Remember, while I strive to provide accurate and current information, it's essential to consult with healthcare professionals for medical advice. | Comparable | 1 | 1 | Not applicable |
| 146. Can i swim after undergoing a total laryngectomy? | After a total laryngectomy, your larynx, which contains the vocal cords, is completely removed. This surgery is typically performed as a treatment for laryngeal cancer. The surgical procedure also involves creating a separate pathway for breathing, known as a tracheostomy or stoma. The stoma is a hole in your neck that connects directly to your trachea, allowing you to breathe.  Since the stoma is open to the environment, you need to be careful to avoid getting water in it, which could lead to a potentially serious situation like choking or lung infection. This doesn't mean you can't participate in water-based activities, but it does mean you need to take extra precautions.  You should not submerge your head under water after a laryngectomy without protecting your stoma. Specialized devices, such as laryngectomy swim collars, can be worn to seal the stoma and protect it from water ingress while you are swimming. These devices should be fitted and used under the guidance of your healthcare provider.  Remember, it's vital to consult your doctor or a speech and language therapist before trying to swim after a laryngectomy. They can provide personalized advice based on your health status and recovery progress. And, as with any exercise after major surgery, it is recommended to start slowly and gradually increase your activity level as your strength and endurance improve.  Safety should be your top priority. Even with protective devices, always ensure you have a companion who knows about your condition and can assist in case of an emergency. | Comparable | 1 | 1 | Not applicable |
| 147. Will I retain my sense of smell after a total laryngectomy? | Total laryngectomy is a surgical procedure that involves the removal of the larynx, commonly known as the voice box, due to conditions like cancer. After a total laryngectomy, the airway is separated from the mouth, nose and throat in a procedure called a tracheostomy. The tracheostoma (stoma) is a hole in the front of the neck that connects the trachea directly to the outside, bypassing the upper airways, including the nose and throat.  Smell and taste are closely linked. When you breathe in through your nose, airborne molecules associated with different scents are picked up by olfactory sensors in your nose. When the air doesn't pass through the nose and upper airway, as happens after a laryngectomy, it may impact your ability to smell and taste.  However, this doesn't mean that you lose the ability to smell and taste completely after a laryngectomy. Some people find that they can still detect some smells and tastes, although it may be reduced or different than before. This happens because some air can still pass through the nose and mouth during activities like swallowing or coughing.  Additionally, some people might learn to use techniques such as "polite yawning," which involves a gentle, controlled yawning action that can help to pull air through the nose and past the olfactory sensors, enhancing the sense of smell.  Overall, the impact on these senses can vary widely between different people. It would be a good idea to discuss this with your doctor or a speech and language therapist, who can provide you with strategies to help maintain these senses as much as possible after a laryngectomy. | Comparable | 1 | 1 | Not applicable |
| 148. Will I retain my sense of taste after a total laryngectomy? | A total laryngectomy, which involves the surgical removal of the larynx or voice box, can affect swallowing and speaking but should not directly impact the sense of taste.  However, because the procedure often involves changes to the anatomy of the throat, there might be indirect effects on taste, primarily due to changes in how you smell and swallow. Smell and taste are closely linked, so if your ability to smell is affected by the surgery, it could change your perception of taste.  Additionally, some people might experience temporary changes in taste due to medications, chemotherapy, radiation treatment, or other factors related to the laryngectomy. But these changes usually aren't permanent.  It's important to discuss all your concerns, including potential changes to your senses of taste and smell, with your healthcare team before surgery. They can provide information tailored to your specific situation and help you understand what to expect during the recovery process. | Comparable | 1 | 1 | Not applicable |
| 149. Can one tonsil be larger than the other for reasons other than cancer? | Yes, one tonsil can indeed be larger than the other for reasons other than cancer. While asymmetrical tonsils can sometimes indicate a medical issue, such as tonsillar cancer, this is relatively rare, particularly in children.  Some common non-cancerous reasons for one tonsil being larger than the other include:   1. Tonsillitis: This is an infection of the tonsils, typically caused by either a viral or bacterial infection. One tonsil may become infected before the other, causing it to swell and appear larger. 2. Tonsil stones (tonsilloliths): These are hard deposits that form in the tonsils. They can cause one tonsil to appear larger than the other. 3. Abscess: A peritonsillar abscess is a bacterial infection that typically develops in the space around one tonsil. This can cause one tonsil to appear larger. 4. Chronic inflammation: Persistent inflammation from conditions like allergies or acid reflux can cause one tonsil to become larger. 5. Congenital asymmetry: It's also possible that one tonsil is naturally larger than the other. Some people are born with asymmetrical tonsils and may never experience any related problems. 6. Cysts or benign growths: Sometimes, noncancerous growths may cause one tonsil to appear larger than the other.   Any persistent asymmetry, pain, or other symptoms associated with the tonsils should be evaluated by a healthcare professional to rule out serious conditions. | Comparable | 1 | 1 | Not applicable |
| 150. Could prolonged use of cocaine potentially cause mucoepidermoid carcinoma? | As of my knowledge cut-off in September 2021, there's no definitive evidence that prolonged use of cocaine directly causes mucoepidermoid carcinoma, which is a type of cancer that arises from the salivary glands.  Cocaine use has many negative health impacts, and it can cause a range of problems in various parts of the body, including the respiratory and cardiovascular systems. Cocaine can also increase the risk of other types of cancers, particularly those associated with the respiratory system, due to the carcinogenic byproducts that may be inhaled when smoking cocaine.  Mucoepidermoid carcinoma, however, is typically not linked with drug use. Known risk factors for this type of cancer include radiation exposure, genetic factors, and possibly prior history of benign salivary gland tumors, but not drug use.  However, it's important to note that our understanding of cancer etiology is constantly evolving, and there may be more recent research or developments on this topic that are not covered in my current knowledge. You should consult with a healthcare professional or conduct a literature search for the most up-to-date information. | Comparable | 1 | 2 | 1 |
| 151. Will I experience dry mouth after a parotidectomy? | Parotidectomy is a surgical procedure to remove the parotid gland, which is one of the major salivary glands. After parotidectomy, some people might experience changes in their saliva production, which could potentially lead to dry mouth, also known as xerostomia. However, the impact on saliva production can vary depending on whether one or both parotid glands are removed, and the extent of the gland removed during surgery.  It's important to remember that the human body has other salivary glands (submandibular and sublingual glands) that continue to produce saliva even after a parotidectomy. This is why many patients do not experience a significant decrease in overall salivation.  But keep in mind, other factors can contribute to dry mouth after surgery as well, such as certain medications, dehydration, or changes in the body's ability to regulate fluid balance while recovering from surgery.  If you're having a parotidectomy and are concerned about potential side effects, it's best to discuss these concerns with your surgeon or healthcare provider. They can provide information based on your specific medical situation and guide you on what to expect during recovery. They might also provide suggestions for managing potential side effects like dry mouth. | Comparable | 1 | 2 | 2 |
| 152. Is a tracheostoma permanent after a total laryngectomy? | A total laryngectomy is a surgical procedure that involves removing the entire larynx, or voice box, usually due to conditions like advanced laryngeal cancer. A part of the procedure includes creating a stoma (opening) in the neck, known as a tracheostoma, which allows the patient to breathe.  Since the larynx, which connects the trachea (windpipe) to the mouth and nasal cavity, is entirely removed during a total laryngectomy, the tracheostoma is usually permanent. This is because the person can no longer breathe through their nose or mouth; they breathe through the stoma instead.  However, technologies and surgical techniques are always advancing, and the specifics can depend on the exact nature of the individual patient's condition and the specific methods used by the surgeon.  It's always important to have detailed discussions with healthcare providers to fully understand the implications of any surgical procedure, its benefits and risks, and how it will affect the patient's life in the short term and long term. | Comparable | 1 | 1 | Not applicable |
| 153. Does having a tracheostoma increase the risk of infection? | Yes, having a tracheostomy (the surgical procedure that results in a tracheostoma, or an opening in the neck leading to the trachea) can increase the risk of certain infections, particularly respiratory infections. The reason for this is because the tracheostoma provides a direct pathway for bacteria and viruses to enter the lower respiratory tract, bypassing many of the body's normal defense mechanisms in the upper airway.  Common infections associated with tracheostomy include:   1. Tracheitis: This is an infection of the trachea that can be caused by bacteria, viruses, or other microorganisms. 2. Tracheobronchitis: This is an infection of the trachea and bronchial tubes. It's typically caused by bacteria, and it's common in people who have a tracheostomy. 3. Pneumonia: This is an infection of the lungs that can be caused by a variety of microorganisms, including bacteria, viruses, and fungi. People with a tracheostomy are at an increased risk of developing pneumonia, particularly ventilator-associated pneumonia if they are on a ventilator. 4. Stoma infection: The skin around the stoma can become infected, leading to symptoms like redness, swelling, and pus.   Proper care and cleaning of the tracheostoma and the tube, as well as regular medical follow-ups, can help reduce the risk of these infections. Health professionals will usually provide instructions and training on how to care for a tracheostomy to minimize these risks. | Comparable | 1 | 1 | Not applicable |
| 154. Is there a possibility of a larynx transplant after a total laryngectomy? | As of my last update in September 2021, larynx transplantation is a technically complex and rare surgical procedure. The first successful larynx transplant occurred in 1998, with other successful cases reported since then. These procedures have typically been performed in individuals with severe laryngeal dysfunction but not usually after total laryngectomy.  There are several challenges to performing larynx transplantation after a total laryngectomy, especially if the laryngectomy was done to treat laryngeal cancer:   1. Availability of Donors: Larynx transplantation requires a suitable donor larynx, which can be difficult to obtain. 2. Immunosuppression: As with all organ transplants, the recipient of a larynx transplant would need to take immunosuppressive drugs to prevent rejection of the new organ. These drugs can have serious side effects and increase the risk of infections and certain types of cancer. 3. Recurrence of Cancer: If the original laryngectomy was performed to treat laryngeal cancer, there may be a concern about the cancer returning. Immunosuppressive drugs could potentially increase the risk of a cancer recurrence. 4. Technical Challenges: The larynx is a complex organ involved in breathing, swallowing, and voice production. Reconstructing these functions with a transplanted larynx can be technically challenging.   As of 2021, the primary focus for most patients who have undergone total laryngectomy is rehabilitation, which can include learning esophageal speech, using an electrolarynx, or having a tracheoesophageal puncture for a voice prosthesis.  However, medical technologies are always advancing, so it is possible that by 2023 or in the future, larynx transplantation after total laryngectomy could become a more feasible procedure. You should consult with a medical professional or research recent literature to get the most up-to-date information. | Comparable | 1 | 1 | Not applicable |
